# Variability of Reproduction Pathways in the Central-European Populations of Hawthorns with Emphasis on Triploids

**DOI:** 10.3390/plants11243497

**Published:** 2022-12-13

**Authors:** Vladislav Kolarčik, Valéria Kocová, Vlastimil Mikoláš, Lenka Mártonfiová, Nikola Hajdučeková, Pavol Mártonfi

**Affiliations:** 1 Institute of Biology and Ecology, Faculty of Science, Pavol Jozef Šafárik University, Mánesova 23, SK-041 54 Košice, Slovakia; 2 Botanical Garden, Pavol Jozef Šafárik University, Mánesova 23, SK-043 52 Košice, Slovakia; 3 Hanojská 4, SK-040 13 Košice, Slovakia; 4 Buzulucká 5, SK-040 22 Košice, Slovakia

**Keywords:** B_III_ hybrids, Central Europe, *Crataegus*, fertilization, flow cytometry, parthenogenesis, polyploidy, pseudogamy

## Abstract

The role of apomeiosis, parthenogenesis, and pseudogamy in the asexual reproduction of some plant groups has not been fully elucidated in relation to species diversification. Quantitative analyses of seed origin may help in gaining better understanding of intercytotypic interactions. Asexual reproduction associated with polyploidy and frequent hybridization plays a crucial role in the evolutionary history of the genus *Crataegus* in North America. In Europe, the genus represents a taxonomically complex and very difficult species group not often studied using a modern biosystematic approach. We investigated the reproduction pathways in mixed-cytotype populations of selected taxa of *Crataegus* in eastern Slovakia, Central Europe. The investigated accessions were characterized by seed production data and the ploidy level of mature plants as well as the embryo and endosperm tissues of their seeds determined via flow cytometry. Diploid and polyploid hawthorns reproduce successfully; they also produce high numbers of seeds. An exception is represented by an almost sterile triploid. Diploids reproduce sexually. Polyploids shift to asexual reproduction, but pseudogamy seems to be essential for regular seed development. In rare cases, fertilization of unreduced gametes occurs, which offers opportunity for the establishment of new polyploid cytotypes between diploid sexuals and polyploid asexuals. Opposite to sexual diploids, triploids are obligate, and tetraploids almost obligate apomicts. Apomixis is considered to help stabilize individual weakly differentiated polyploid microspecies. Pseudogamy is a common feature and usually leads to unbalanced maternal to paternal contribution in the endosperm of triploid accessions. Parental contribution to endosperm gene dosage is somehow relaxed in triploids. Our *Crataegus* plant system resembles reproduction in the diploids and polyploids of North American hawthorns. Our data provide support for the hypothesis that polyploidization, shifts in reproduction modes, and hybridization shape the genus diversity also in Central Europe.

## 1. Introduction

Polyploidization and hybridization are two rapid speciation processes [1,2] that play an eminent role in the evolution of angiosperms [3,4,5,6,7]. Both may lead to shifts in the variety of anatomical, biochemical and molecular plant traits, as well as changes in life-history traits and reproduction systems [5,8,9]. For instance, the sexual reproduction system is disrupted after polyploidization or hybridization, e.g., meiosis may be irregular, which results in reduced fertility or even sterility, but asexual reproduction may evolve and stabilize novel polyploid or hybrid lineage through selection [10] to maintain reproductive function. Newly evolved asexual reproduction system in plants may significantly influence their subsequent evolutionary fate [11], e.g., self-compatible asexuals may reproduce without a mating partner, which is an advantage in the colonization of new regions [12,13]. Along with predominant sexuality, the presence of clonality, apomixis or hemisexuality, all found in diverse forms, was proven in many polyploid angiosperms.

The study of diverse asexual reproduction systems that have evolved in angiosperms is gaining increasing attention from the plant reproduction scientific community (e.g., [14]). A brief survey of plant asexual reproduction research in the last decades (e.g., [15,16,17,18,19,20,21,22,23]) indicated that the studies seem to focus mainly on: (i) the description of various forms of asexuality, their evolution, spatial distribution of asexual biotypes, and frequency of occurrence in natural populations; (ii) the study of the coexistence of sexual and asexual forms and their interaction (e.g., gene flow presence vs. absence, ecological competitiveness); (iii) the unraveling of the genetic background of both types and how this is reflected in their fitness and evolutionary success; and (iv) the elucidation of the relationships between polyploidy and asexuality evolution. Reproductive system variation and the associated phenomena, e.g., apomeiosis, parthenogenesis vs. fertilization, pseudogamy, polyspermy, and endosperm gene-dosage unbalance, are rarely studied quantitatively, but this approach may be beneficial to better understand the relationships of apomictic components and inter-cytotype interactions and to better design further studies on gene flow, population genetics, and “omics” data in asexual plants [24,25].

A notable example of a genus with common asexuality is *Crataegus* L., which belongs to the family Rosaceae (~100 genera, ~3000 species [26]), subfamily Amygdaloideae Arn., tribe Maleae Small (~26 genera [26]) and subtribe Malinae Reveal (~950 species [27,28,29]; for nomenclature see [30]). Other genera of Malinae, e.g., *Amelanchier* Medik., *Cotoneaster* Medik., and *Sorbus* L. behave reproductively similar as *Crataegus* [31,32,33,34]. *Crataegus* is a monophyletic, well-defined genus [35] with recent occurrence in Eurasia, North Africa, North and Central America, and northern parts of South America and contains ca. 140–1200 species depending on the species concept [35,36]. The members of *Crataegus* are shrubs and small trees that usually occupy ruderal sites and are used as the main component of hedgerows [35,37,38]. They are insect-pollinated and produce fruits—polypyrenous drupes [35]. The genus has been well known for its tremendous morphological variation and unresolved systematic and evolutionary relationships between species and species groups. Studies performed in the last decades in North America demonstrated that polyploidization and frequent hybridization, coupled with the occurrence of apomixis in polyploids, play an important role in the increase in hawthorn cytotypic and morphological diversity [39,40,41,42,43,44]. Whereas the reproductive system of the genus has been continuously characterized in North America and has been helpful in understanding the biological diversification of polyploid taxa, this approach has been scarcely applied to study European hawthorns (e.g., cytoembryology [45,46]; reproduction systems [47]; but see many studies published on chorology [48,49]; morphology [48]; ecology [50]; seedling performance [51]; phenology [52]; palynology [53]; genetic diversity [54,55,56]; secondary metabolites [57,58,59]).

Reproduction systems differ between diploids and polyploids of *Crataegus*. Studies performed so far have revealed 2x, 3x, 4x, and rarely 5x and 6x hawthorns; euploidy seems to be the rule, but aneuploids cannot be excluded [39]. Allopolyploids are quite common, and autopolyploids also exist (e.g., *C. suksdorfii* (Sarg.) Kruschke with 2x, 3x, and 4x [60]). Some series in the genus contain only polyploid species (e.g., *Pruinosae* (Sarg.) Rehder and *Tenuifoliae* (Sarg.) Rehder [39]). The reproduction features of hawthorns include both sexuality common in diploids and asexuality prevalent in polyploids. There is a strong association between polyploidy and asexuality observed in many plant groups, including *Crataegus* [31,40,61,62,63]. Asexuality in hawthorns is characterized by the presence of gametophytic apomixis, formation of new individuals—clones of mother plant. It involves three components: (i) apospory (rarely diplospory), which is a rise in unreduced female gametophyte, embryo sac, from an unreduced somatic cell within the nuclear tissue in the ovary (or from unreduced megaspore in diplospory); (ii) parthenogenesis, fertilization bypassing the development of an embryo from the unreduced egg cell in the embryo sac; and (iii) pseudogamy, fertilization of the central cell of the embryo sac with sperm cell, which results in functional nutritive tissue for the embryo (endosperm) [40,45,46,64,65]. Diploids of *Crataegus* are sexual, meiosis is regular, and egg cell is normally fertilized [40,47]. In the polyploids of *Crataegus*, apospory prevails and is almost always connected with pseudogamy [40,66]. Both sperm cells from pollen grain can contribute to the endosperm origin of *Crataegus*, or rarely one sperm cell can be utilized to fertilize the unreduced egg cell, resulting in an increase in the offspring ploidy level [40] in so-called B_III_ hybrids. Their presence increases the cytotypic diversity within populations. Hybrids of a diploid sexual species can develop into near-sterile triploids with some apomictic traits [66,67]. These can be the source of apomictic triploid species, which can be possibly established by selection [10]. In general, triploid apomictic species of *Crataegus* or other genera and their reproduction systems are rarely studied (but see, for instance [34,40,47,62,68,69]). With regard to *Crataegus*, Talent and Dickinson [40,62] have suggested that they are probably pollen-sterile and can accept pollen from co-occurring diploid or tetraploid species, which is necessary for the origin of pseudogamic endosperm and induction of parthenogenetic development of egg cell into embryo. However, Vašková and Kolarčik [47] have demonstrated that some triploids are able to produce reduced pollen, probably highly unbalanced aneuploid ~1.5x, which is functional in terms of the pseudogamic origin of endosperm. The variation in reproduction systems of *Crataegus* in North America includes also obligate and facultative apomicts. Some species are capable of both sexual and asexual reproduction. While *C. crus-galli* L. and *C. macracantha* Lodd. ex Loudon are almost obligate apomicts, tetraploid species *C. submollis* Sarg. is predominantly sexual, i.e., the seeds show much higher rates of meiotic reduction than in other polyploid *Crataegus* [62]. The variation in reproduction modes could be seen as a pivotal component to the origin of microevolutionary complexity present in sexual–asexual, diploid–polyploid plant systems [69]. Quantitative analysis of reproduction systems is now possible thanks to rapid seed sample preparation and precise analysis of DNA content via flow cytometry (FCM).

The flow cytometric seed screen (FCSS) has significantly influenced our knowledge of reproduction systems in asexual plants. It is an easy and inexpensive method and has become routine and very important in genetic research to determine the ploidy level of plants [70] and estimate their seed origin [71] via comparative measurement of their maternal tissue, embryo, and endosperm DNA content. Due to the presence of abundant and persistent endosperms [72], FCSS was successfully applied in the study of reproduction systems in many genera of Rosaceae [31,32,33,34,73,74,75,76], including the genus *Crataegus* [39,40,47,60,62].

Here we document the reproduction features under natural conditions of the diploid–polyploid plant system of the genus *Crataegus* in Central Europe with a special focus on triploid plants, for which we have only fragmented information from the studies in the region of North America. Specifically, we aim: (i) to assess the reproduction success of various diploid, triploid, and tetraploid taxa within selected mixed-cytotype populations in eastern Slovakia; (ii) to determine the frequency of meiosis and apomeiosis in diploids and polyploids; (iii) to reveal the role of pseudogamy in apomicticaly reproducing taxa; (iv) to reconstruct the ploidy level of sperm cells participating in fertilization of central cell (pseudogamy); (v) to estimate variation in the reproduction mode of the sampled taxa at the individual level, e.g., variation in the frequency of meiosis, pseudogamy, and fertilization of egg cell (possibly including the origin of B_III_ hybrids in polyploids); and (vi) to estimate possibly the participation of various pollen cytotypes in the fertilization of diploid and polyploid hawthorns.

## 2. Materials and Methods

### 2.1. Plant Material

**Figure 1:**
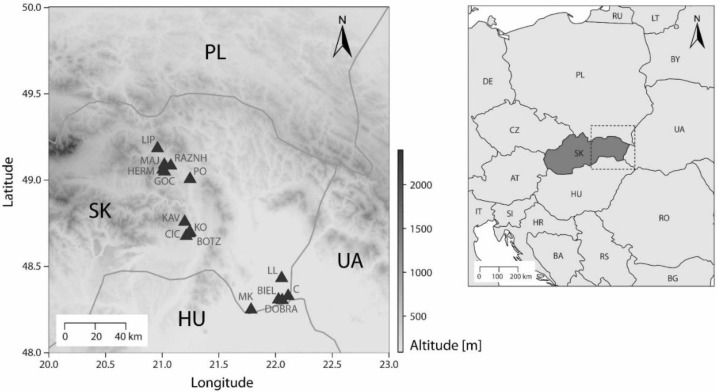
Location of the 15 investigated populations in eastern Slovakia, Central Europe. The population codes are as presented in Table 1 and Supplementary Material Note S1.

Four species, *C. monogyna* Jacq. (2x), *C.* × *kyrtostyla* Fingerh. s. Holub (2x and 3x, = *C. monogyna* × *C. subsphaerica*, for simplicity hereafter referred to as *C. kyrtostyla*), *C. rhipidophylla* Gand. (3x), *C. subsphaerica* Gand. s.l. (3x, = *C. monogyna* × *C. rhipidophylla*), and apparent multiple hybrids (3x and 4x) of *C. kyrtostyla*, *C. subsphaerica*, and *C. laevigata* (Poir.) DC. were included in our sampling (Table 1 and Table 2, we refer to [35,77] for the morphological variation in leaves and fruits). In total, 48 mature fruiting hawthorns originating from 15 populations ([78], Figure 1, Supplementary Material Note S1) were studied. Mature plants were selected to cover (i) various morphologically diverse taxa, especially triploids, and (ii) those accessible individuals that produced a considerable number of fruits easy to collect. The rate of seed production of 14 diploid and 20 triploid mature representatives (mother plants) and the reproduction characteristics of 19 diploid, 20 triploid, and 8 tetraploid individuals were investigated. All the fruiting trees were sampled in autumn during the period of 2010–2020; all mother plants were open-pollinated to prevent unintentional deviation from the natural situation caused by artificial treatment. Voucher specimens of the 48 mother plants are deposited in the herbarium of Botanical Garden of Pavol Jozef Šafárik University in Košice (KO) or in the personal herbarium of the third author (V.M.).

### 2.2. Evaluation of Reproduction Success through Seed Formation

The number of pyrenes per fruit and number of seeds per fruit were scored to assess reproduction success of plants. In total, 319 (11–33 per plant) and 2083 (32–239 per plant) fruits for diploids and triploids, respectively, were evaluated. Unfortunately, tetraploids were not precisely assessed, but both fruits and seeds were numerous.

### 2.3. Genome Size Quantification of Mature Plants

Mature plants (48 trees in total) that were studied for the reproduction characteristics were subjected to the FCM genome size determination to infer their ploidy level. The plants were analyzed each individually; we also prepared a “bulked” sample consisting of three simultaneously processed individuals to speed up the process. This triplet was analyzed with internal reference standard (IRS) to first determine if the individuals had the same ploidy level. If so, the analysis had been conducted until a significant number of nuclei was collected. If the “bulked” sample contained plants of at least two different ploidy levels, then each plant was analyzed again separately with IRS. *Pisum sativum* cv. Ctirad (2C = 9.09 pg; [79]) or *Solanum pseudocapsicum* (2C = 2.59 pg; [80]) were used as IRS. Sample preparation was performed according to Loureiro et al. [81] in line with modifications of Vašková and Kolarčik [47]. In the present study, cell nuclei were isolated from approximately 0.5-cm^2^ piece of exocarp isolated from mature fruits of a *Crataegus* plant (mother tree). FCM samples were measured using the Partec CyFlow ML (Partec GmbH, Münster, Germany) flow cytometer (Institute of Biological and Ecological Sciences, Pavol Jozef Šafárik University in Košice, Slovakia) equipped with a 532-nm (150 mW) green laser. One to five replicates per sample were made, each with at least 1000 measured nuclei each of sample and standard. Measurements were performed using FloMax ver. 2.70 (Partec GmbH, Münster, Germany) or FlowJo ver. 10.1 (FlowJo LLC, Ashland, Wilmington, DE, USA). Regarding the quality of histograms (CV below 5% and symmetric peaks of both the sample and standard), we decided to use further only the best representative measurement of each mother plant. DNA content (2C value) was determined using the following formula [82]: DNA amount of sample = DNA amount of internal standard × [(G_0_/G_1_ peak mean of sample)/(G_0_/G_1_ peak mean of internal standard)] (G_0_/G_1_ refers to the population of nuclei in the G_0_ or G_1_ phases of the cell cycle). The ploidy level was than inferred based on the obtained DNA amount data (2C values) interpreted with the help of the DNA amount ranges for the cytotypes provided by Talent and Dickinson [39]: 1.37–1.67 pg for diploids, 2.05–2.51 pg for triploids, 2.74–3.34 pg for tetraploids, and 3.42–4.18 pg for pentaploids.

The cytotypic composition of the studied populations was determined in a previous study of ploidy level [78], which will be published in detail elsewhere.

### 2.4. Flow Cytometric Seed Screen, Sample Preparations and Measurements

To reconstruct the reproduction pathways of the investigated mother plants, we studied their seed families by comparing the embryo and endosperm ploidy level using the FCSS [71]. To estimate variation in the reproduction systems of the sampled taxa at the individual level, e.g., frequency of meiosis, pseudogamy, and B_III_ hybrids (embryo with increased ploidy level compared with the mother plant), our sampling covered many fruits (and seeds) per plant of few mother individuals rather than few fruits per plant of many mother individuals. This approach resulted in the collection of 258, 1202 and 114 seeds of 19 diploid, 20 triploid, and 8 tetraploid mother trees, respectively. The diploid mother trees were represented by 5–20 seeds per tree. Eighteen triploid mother trees were represented by 18–138 seeds per tree, and in the remaining two trees, only 5 seeds were analyzed due to the low sample size for the FCSS (in the plant coded 1447/11) or evident lack of developed seeds (in the plant coded kyrtoA), which probably stems from the seed sterility of this accession. Eight tetraploids were sampled for 5–27 seeds per plant.

Our pilot experiments showed that FCM measurement of a “bulked” sample (consisting of at least 2 but usually ≥5 simultaneously analyzed seeds) of diploid mother plants commonly resulted in the consistent measurement of embryo and endosperm DNA content among the samples; therefore, we here combined 5 seeds into the “bulked” sample. In all cases, a part of isolated embryos together with the reference standard and separately a part of embryos with a part of endosperms were used. If we found mixed-cytotype composition of the “bulked” sample (the presence of several ploidy levels among embryos or endosperms), each seed was then analyzed separately using the rest of the available tissues. Pilot trials of the FCSS analysis of “bulked” samples of polyploid mother plants resulted in the detection of several peaks in FCM histograms, which were difficult to interpret. These experiments showed that the reproduction modes of most of polyploid *Crataegus* have mixed characteristics, often a combination of parthenogenesis, fertilization of unreduced gametes (origin of B_III_ hybrids), and pseudogamy probably involving sperm cells of a different ploidy level. Therefore, the “bulked” sample preparation does not allow precise identification of the reproduction mode of particular seeds, so we rather approached the problem using the time-consuming but precise method of individual FCM measurement of each seed individually according to the practice of most recent studies [24,33,34,40,73,74,83].

The samples for FCSS were prepared similarly to the ploidy level screening of mature plants (see above). The seeds were exempted from pyrene mechanicaly using vise device (Supplementary Material Figure S1). Usually, a half of a seed (half of an embryo, half of a seed coat with an endosperm layer attached [40]) was co-chopped with the reference standard (*Pisum sativum* cv. Ctirad, 2C = 9.09 pg [79]) leaf tissue of approximately 0.5 cm^2^ using a razor blade in a Petri dish containing 1 mL of general purpose buffer (GPB) [81]. The suspension was then filtered through a 42-μm nylon filter and stained, incubated, and measured as described by Vašková a Kolarčik [47] with minor modifications. At least 1300 particles were collected for embryo G_0_/G_1_ peak in each measurement. The measurements where the coefficient of variation (CV) of embryo or standard peak was above 6%, or above the more relaxed criterium of 9% in the case of endosperm, were discarded from further data interpretation and analyses.

The resulting FCM histograms of the most analyzed seeds included peaks identifiable with the populations of nuclei derived from the embryo, endosperm, and IRS; however, in several cases, when only two peaks were recorded, separate analyses of embryo + IRS and embryo + endosperm were conducted to verify whether some additional peaks were not overimposed by the G_2_ peak of the IRS. This verification was performed also in the cases when primary measurement showed no endosperm peak. In such analyses, the measurements revealed the endosperm peak observed in the position of the IRS peak of the combined analysis of embryo + endosperm + IRS, but in a few cases, the endosperm was not detected. Moreover, if primary analyses revealed multiple embryos or endosperms in a single seed, confirmatory analyses based on the remaining half of the seed material were performed. The ploidy level of the embryo and endosperm and sperm cells which fertilized egg or central cell was inferred based on a series of calculations motivated by the study by Dobeš et al. [74] and the reproduction pathways were then interpreted according to Matzk et al. [71,84] (see also Supplementary Material Note S2). We were precise as much as possible as recent studies demonstrated that reduced unbalanced and aneuploid pollen was formed in triploid plants of *Crataegus* or *Sorbus* [34,47].

### 2.5. Flow Cytometric Seed Screen Data Analyses

FCSS data statistical analyses were conducted using the R ver. 3.5.3 environment [85]. Our analyses revealed negligible variation in reproduction pathways (variation in particular embryo and endosperm ploidy level) among diploids. A small sample size for tetraploids does not allow for a meaningful within-cytotypic comparison (Table 2). Diploids and tetraploids were therefore excluded from the statistical analyses.

The relationships among triploid mother plants based on the reproduction pathways were assessed via detrended correspondence analysis (DCA) and hierarchical clustering (HC). DCA was conducted on matrix, mother trees × seed categories, of the percentage proportion of particular seed categories which were found relatively abundant in the dataset (≥4 cases). DCA was conducted in vegan package (ver. 2.5-4 [86]) using the *decorana* function with default settings (e.g., datamatrix was not column-weighted). HC was performed in conjunction with heatmap visualization of proportional data. Prior to HC, the columns of the datamatrix were transformed so that the values were expressed as a proportion of the maximum value in the column (in vegan), and dissimilarity structure was extracted based on the Bray–Curtis dissimilarity index (in vegan). The dissimilarity structure was subjected to HC, which was done using the ward agglomeration method. The transformed datamatrix was displayed as a heatmap in the corrplot package (ver. 0.92 [87]).

## 3. Results

### 3.1. Evaluation of Seed Production in Diploids and Polyploids

**Figure 2:**
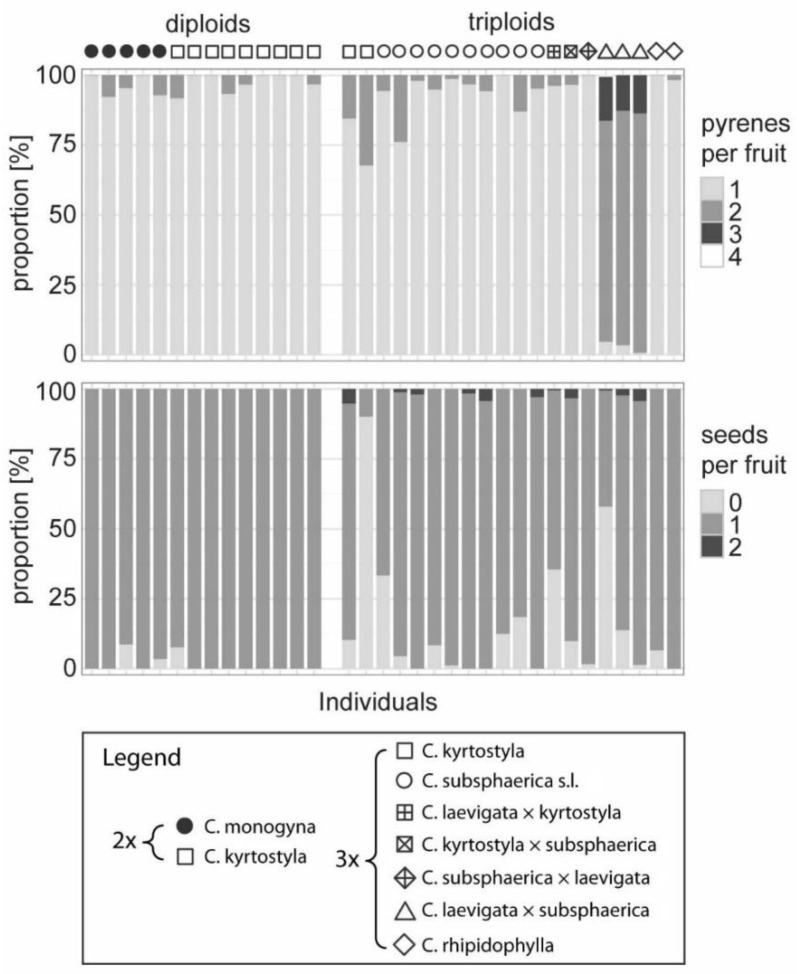
Fruit characteristics of diploid and triploid individuals. Number of pyrenes (**upper** panel) and well developed and healthy seeds (**lower** panel) per fruit expressed as percentage data for 14 diploid and 20 triploid individuals.

Seeds were frequently present in fruits from plants of all cytotypes, diploids, triploids, and tetraploids. The fruits of the studied accessions often contained only one seed (64.02–100.00% in all accessions) and rarely even two (0.42–5.17% in 11 accessions) despite some fruits containing two (or even three or four) pyrenes (Figure 2). In 14 accessions, 1.25–35.56% of fruits produced empty pyrenes without any well-developed seed. Two exceptions showed different patterns with a high proportion of fruits with empty pyrenes (>50%), namely, Vyhon4 and kyrtoA (in later even 90.08%). Although the pyrenes and the seeds were not counted precisely in the fruits of tetraploids, both were numerous.

### 3.2. Flow Cytometric Seed Screen of Diploid Mother Plants

**Figure 3:**
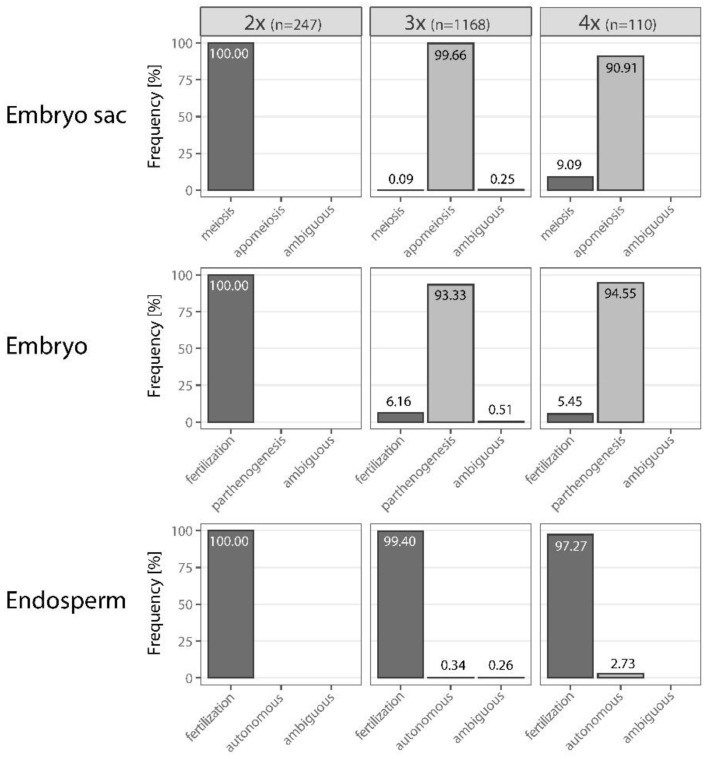
Survey of reproduction systems in diploid (2x), triploid (3x), and tetraploid (4x) hawthorns. The frequency of origins for the embryo sac, embryo, and endosperm is given in percentage.

The FCSS of the diploid mother trees was successful, for 247 seeds (95.74%) ploidy level of both embryo and endosperm were determined and further interpretation of their origin was possible. Together, 242 2x and five 3x embryos as well as 240 3x, five 4x and two 6x endosperms were found. The inferred seed categories are presented in Table 3 (see also Supplementary Material Table S1). All the seeds originated from reduced embryo sac, and the egg and central cells were always fertilized (Figure 3). The majority of seeds, with 2x_emb_/3x_end_, were interpreted as sexually derived seeds that originated from a reduced embryo sac with 1x egg cell and a binucleate (2x) central cell and double-fertilized with two reduced 1x sperm cells. In the remaining cases, the seeds were also sexually derived, but fertilization by 2x sperm cells (3x_emb_/4x_end_) or endopolyploidization of newly formed endosperm (2x_emb_/6x_end_) occurred (Table 3). We cannot exclude the possibility that seeds with 2x_emb_/6x_end_ combination are of asexual origin, but this explanation seems to be less probable than endosperm endopolyploidization (see below).

### 3.3. Flow Cytometric Seed Screen of Polyploid Mother Plants

**Figure 4:**
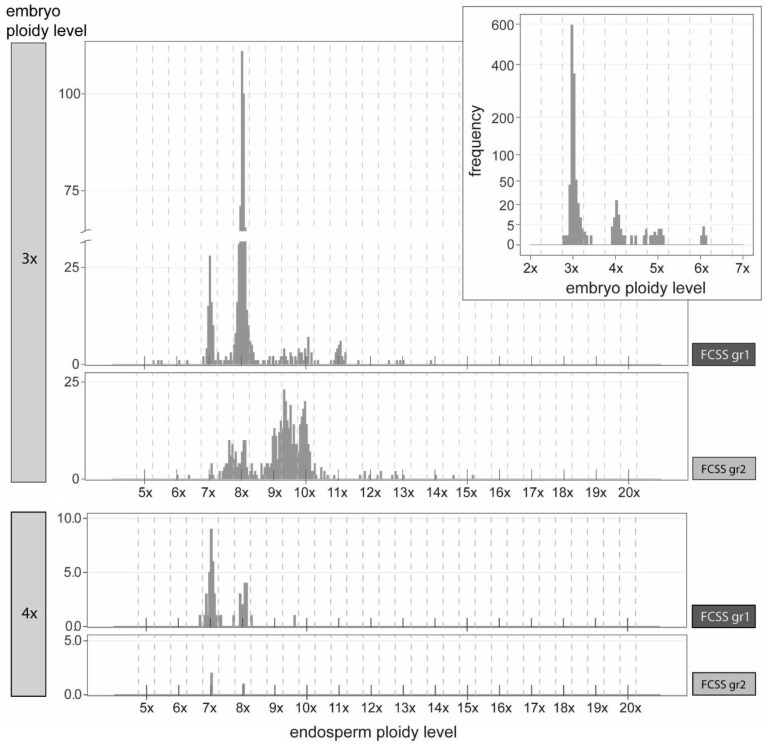
Calculated embryo (in inset) and endosperm ploidy levels from 20 triploid mother trees of *Crataegus* assigned to two FCSS groups, namely, FCSSgr1 and FCSSgr2, according to their reproduction pathways. The estimated euploid and aneuploid ploidy level categories are separated by vertical dashed lines. For simplicity, the endosperm ploidy level variation is shown only for the most abundant seed categories with 3x and 4x embryos.

In almost all cases, the FCSS measurements of polyploids showed one peak for embryo and one peak for endosperm. The FCSS of triploids allowed the identification of the ploidy level for both embryo and endosperm for 1168 seeds (97.17%). For the remaining 34 seeds, embryo or endosperm signal was missing in 27 seeds, two signals for embryo appeared in one seed, the FCSS of five seeds showed two signals for endosperm, and one measurement detected even three endosperm signals. Due to the difficult interpretation, all these 34 seeds were excluded from further analyses. While the embryo ploidy level categories were quite discrete and only 10 seeds (from 1168) were assigned to the “aneuploid” categories ~3.5x and ~4.5x, the situation for the endosperm ploidy level was different. We could not identify the discrete ploidy levels of endosperm; apparently, only the peaks for 7x, 8x, 9x–~9.5x, and 10x appeared clearly (Figure 4). Altogether, 1168 seeds can be assigned to six embryo ploidy categories 3x, ~3.5x, 4x, ~4.5x, 5x, and 6x and 23 endosperm ploidy categories 5x, ~5.5x, 6x, ~6.5x, 7x, ~7.5x, 8x, ~8.5x, 9x, ~9.5x, 10x, ~10.5x, 11x, ~11.5x, 12x, ~12.5x, 13x, 14x, ~14.5x, 15x, ~15.5x, ~17.5x, and ~19.5x.

**Figure 5:**
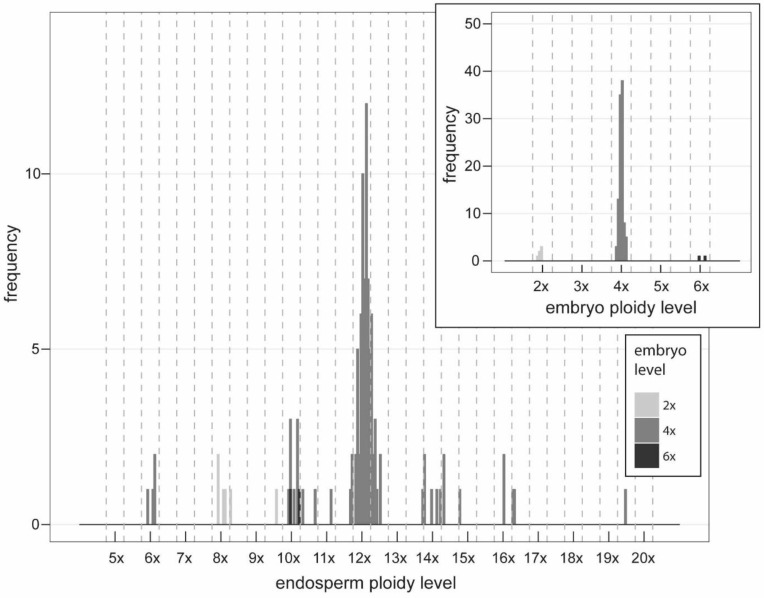
Calculated embryo (in inset) and endosperm ploidy levels from 8 tetraploid mother trees of *Crataegus*. Estimated euploid and aneuploid ploidy level categories are separated by vertical dashed lines.

In total, 110 seeds of tetraploids (96.49%) were successfully screened. The endosperm signal was missing in four seeds, and these were excluded from the analyses. We have observed well-identifiable embryo and endosperm categories in tetraploids, with few exceptions apparently of higher ploidy level (Figure 5). The seed categories are presented in Table 3. We found 2x, 4x, and 6x embryos and 6x, 8x, ~8.5x, ~9.5x, 10x, ~10.5x, 11x, ~11.5x, 12x, ~12.5x, ~13.5x, 14x, ~14.5x, 15x, 16x, ~16.5x, and ~19.5x endosperms among all 110 seeds.

We note that such a precise identification of ploidy level in the cases of >12x endosperms is only putative because the identification error is increasing in higher ploidy levels [40].

### 3.4. Reproduction Modes in Triploids

In most of the seeds produced by the triploids, ≥3x embryo and ≥6x endosperm (1164 seeds, 99.66%, Figure 3 and Figure 4, Table 3; see also Supplementary Material Table S1) were found, suggesting that triploids mostly produce seeds asexually from unreduced embryo sac with 3x egg cell and binucleate 6x central cell. Only one seed with ~3.5x_emb_/5x_end_ (0.09%) was found, suggesting rare meiotic origin of embryo sac. Additional three seeds with 3x_emb_/~5.5x_end_ were found, however, the DNA content of ~5.5x endosperm was close to 6x, and we could not exclude FCM measurement errors. Therefore, these were not interpreted as unequivocal evidence for the meiotic origin of the embryo sac. We also excluded from the interpretation three seeds where ~3.5x embryo and 9x, 10x, and 12x endosperm were found. Here again rare measurement error may be involved, DNA content of ~3.5x embryo is close to 3x. Altogether, 71 seeds (6.08%) contained 4x, ~4.5x, or 5x embryo and ≥6x endosperm. The origin of embryos was interpreted as the result of fertilization of unreduced egg cell with one 1x, ~1.5x, or 2x sperm cell (Figure 4, Table 3). With one sexually derived seed (see above), fertilization of egg cell occurred in 72 seeds altogether (6.16%). In six seeds (0.51%), 6x embryo was found, which along with the higher ploidy levels of their endosperm (≥14x) suggested the presence of an unreduced and endoreplicated embryo sac or alternatively simultaneous endoreplication of embryo and endosperm.

Four seeds with 3x_emb_/6x–~6.5x_end_ (0.34%) may be interpreted as the only ones without any contribution of sperm cells in central cell fertilization, i.e., autonomous endosperm formation. In the rest of the seeds (1161, 99.40%), except three of 3x_emb_/~5.5x_end_ (0.26%), whose origin is questionable, the central cell was always fertilized.

**Figure 6:**
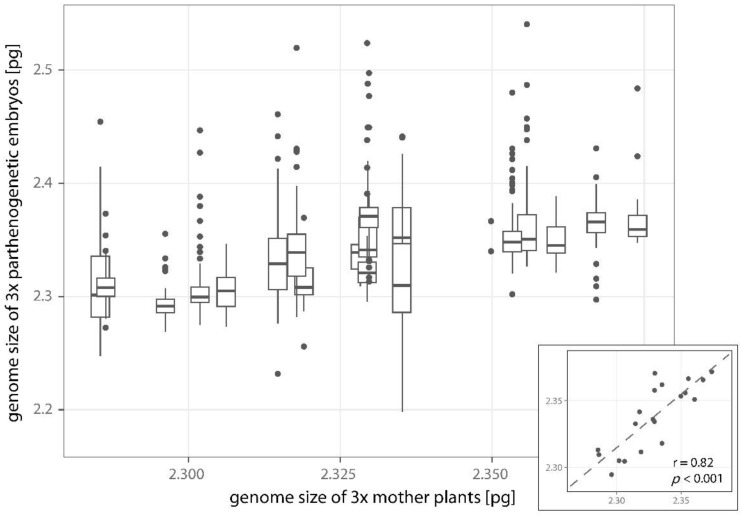
Genome size comparison of mature mother plants and their parthenogenetic (3x) embryo families in triploids. The lower and upper hinges of boxplot correspond to the first and third quartiles (delimit inter-quartile range—IQR), whiskers extend to ±1.5 × IQR and dots represent outliers. In inset; dots represent mean values, dashed line indicates regression line; the value of Spearman rank correlation coefficient r and associated *p*-value are reported.

The ploidy level of pollen grains contributing to the origin of embryo and endosperm may be various in the seeds of triploids. The most abundant seed types are reported in Table 3. The ploidy level categories determined for triploid embryos were quite discrete, 3x, 4x, 5x, and 6x, and additional intermediate ~3.5x and ~4.5x cytotypes were rare (Figure 4). For endosperms, only the peaks for 7x, 8x, 9x–~9.5x, and 10x ploidy categories clearly appeared (Figure 4), but they are not very discrete. This is difficult to interpret but seems to be consistent with the idea that triploids are able to accept viable reduced pollen grains of diploids (1x, origin of 7x and 8x endosperm), tetraploids (2x, origin of 8x and 10x endosperm), and the range of unbalanced aneuploid pollen grains, ~1.5x (maybe even ~2.5x as seen in seeds 3x_emb_/11x–~11.5x_end_), and the latter are likely produced on triploid mother plants [34,88,89]. This was also suggested to explain pollen size variation [46] and FCM pattern [47] in 3x *Crataegus*. Another contribution to this variability may stem from the genome size variation observed in triploids (Figure 6). This means that even the pollen with the same number of chromosomes may differ in the genome size between accessions and thus contribute to the variation in the endosperm DNA content. A total of 26 seeds with ≥12x endosperm may indicate the endosperm with endoreplicated genome of the central cell (6x to 12x).

The fertilization of egg cell (the origin of 4x, ~4.5x, or 5x embryo) occurred in 6.08% of the cases (B_III_ hybrids) and always by a single sperm cell and together with the fertilization of the central cell. The ploidy levels of endosperm in several seeds with higher embryo ploidy level (24 seeds of 4x_emb_/8x_end_, 4x_emb_/10x_end_) indicate the central cell fertilization by two sperm cells, which advocates for the presence of polyspermy (Table 3).

### 3.5. Reproduction Modes in Tetraploids

Tetraploids produced mostly seeds with 4x embryo and >8x endosperm (98 seeds, 89.09%, Figure 3 and Figure 5, Table 3; see also Supplementary Material Table S1). With two 6x_emb_/10x_end_ seeds, they altogether (100 seeds, 90.91%) suggest that tetraploids produce unreduced embryo sacs with 4x egg cell and binucleate 8x central cell. The meiotic origin of embryo sac was found in the rest of the seeds (10 seeds of 2x_emb_/8x–~9.5x_end_, and 4x_emb_/6x_end_, 9.09%). Most of the reduced (2x) and unreduced (4x) egg cells developed into embryo parthenogenetically (94.55%). Fertilization of reduced and unreduced egg cell occurred in six cases (5.45%); four seeds 4x_emb_/6x_end_ (endosperm to embryo ratio 1.5) exhibited residual sexuality (rare presence of meiosis and double fertilization of the egg and central cells), and two 6x_emb_/10x_end_ seeds documented the origin of B_III_ hybrids, embryo origin from the fertilization of unreduced egg cell. Autonomous endosperm development has been evidenced only in three seeds 4x_emb_/16x–~16.5x_end_ (2.73%); endosperm originated probably from 8x central cell through endoreplication. Alternatively, trinucleate central cell double-fertilized with two 2x sperm cells may explain 16x and ~16.5x endosperm. Five seeds of 2x_emb_/8x_end_ and one seed of 2x_emb_/~9.5x_end_ indicated rare meiotically derived egg cells, which developed parthenogenetically into an embryo (reduced parthenogenesis).

All of the common seed types found in tetraploids were easy to interpret as a result of an unreduced embryo sac with a parthenogenetically developing egg cell into an embryo (4x) and a fertilized binucleate (8x) central cell by one or two reduced 2x sperm cells (10x or 12x endosperm, pseudogamy) (Figure 5, Table 3). Two reduced sperm cells (2x + 2x, or ~2x + ~2x) were found to be involved in the origin of 8x and ~9.5x endosperms in the seeds of 2x_emb_/8x–~9.5x_end_. Further interpretation for few FCSS records with >~12.5x endosperm (altogether 14 cases) has suggested either the trinucleate central cell of the embryo sac or the fertilization of the central cell by unreduced sperm cells (3x or 4x), the later explanation being less probable. Finally, the fertilization of the central cell by two unbalanced ~1.5x sperm cell (~10.5x and 11x endosperm, 2 cases) was also evidenced.

### 3.6. Difference in the Proportion of the Seed Categories among Triploids

**Figure 7:**
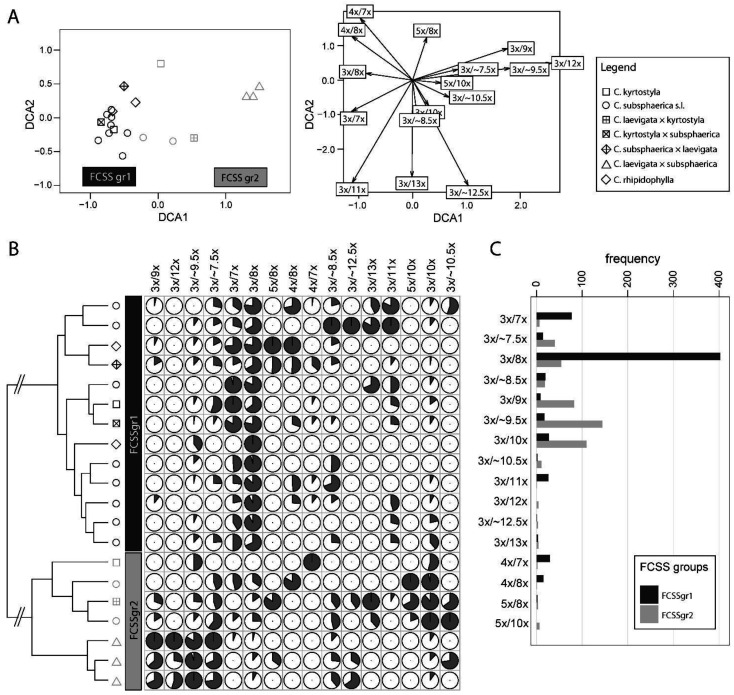
Interindividual quantitative variation in the embryo and endosperm formation of the triploids of *Crataegus* allows the recognition of two groups of plants, FCSSgr1 and FCSSgr2. (**A**) Detrended correspondence analysis (DCA) of 20 individual plants based on the embryo and endosperm ploidy level data of their seeds. The scores for the individuals are shown on the left panel and those for the seed categories on the right. The seed categories are expressed as ploidy level of embryo before slash and ploidy level of endosperm after slash. (**B**) Hierarchical clustering (HC) of 20 individuals based on the embryo and endosperm ploidy level data of their seeds. The data were percent maximum transformed on the columns prior to HC and then displayed as heatmap. The seed categories are expressed as in A. (**C**) Barplot of the frequency data of the most frequent seed categories (≥4 cases) in FCSSgr1 and FCSSgr2. Seed categories are expressed as in A.

The DCA ordination plot shows a weak separation of the triploid mother plants according to the first DCA axis, which corresponds to the HC results (Figure 7). In the cluster analyses, two groups were resolved, namely, FCSSgr1 and FCSSgr2. Both groups differed in the proportion of some seed categories (Figure 7), and each contained 664 and 504 seeds, respectively. The most numerous seed categories (represented by ≥4 seeds) are presented as barplots and compared between the two groups (Figure 7C). Some tendencies are clear. Mother trees assigned to FCSSgr1 likely produced seeds with the endosperm originating from fertilization mostly by euploid sperm cells, either one or two 1x (3x_emb_/7x_end_, 3x_emb_/8x_end_) or one 2x sperm cell (3x_emb_/8x_end_). On the other hand, the members of FCSSgr2 had the tendency to form seeds with the endosperm originating from fertilization by two euploid 1x and 2x sperm cells (3x_emb_/8x_end_, 3x_emb_/10x_end_), and supposedly unbalanced (and aneuploid) ~1.5x sperm cells (3x_emb_/9x_end_, 3x_emb_/~9.5x_end_) or possibly by one 2x or 3x sperm cell (3x_emb_/8x_end_, 3x_emb_/9x_end_).

## 4. Discussion

### 4.1. Reproduction Characteristics of a Diploid–Polyploid Plant System in Crataegus

The genus *Crataegus* is notoriously known for its high taxonomic complexity, the subjectivity in the species circumscription, and the morphological variability [35,62] due to frequent hybridization and asexual reproduction [35,40,41,43,47,48,62]. Diverse reproduction systems influence species’ genetic background and may drive future evolutionary trajectory of plants [11], but particular aspects of asexual reproduction, e.g., frequency of meiosis/apomeiosis, frequency of B_III_ hybrids or origin of nutritive tissue—endosperm in particular cytotypes, and their precise ways of formation have been rarely investigated. Quantitative analyses of reproduction systems in natural populations may shed light into this issue but they are rarely reported. Here we characterized the rate of seed production and applied the FCSS to characterize the reproduction modes in diploid and polyploid accessions of *Crataegus* from mixed-cytotype populations in eastern Slovakia (Central Europe). Our genome size and ploidy level data on the same populations ([78], Table 1) have documented that natural hawthorn populations are cytogenetically very diverse (2x, 3x, and 4x among mature trees) and cytotypic proportions apparently vary among them.

In summary, the results of this study indicated that the plant system studied here is reproductively characterized by the following:

- Sexual reproduction of diploids with reduced 1x embryo sac and double fertilization (both the egg and central cells) by reduced 1x sperm cells. Very rare fertilization by unreduced 2x pollen (or less probably reduced one originated from 3x or 4x plants) is also possible;
- Most of the polyploids (triploids and tetraploids) are almost obligate apomicts. An exception is represented by the rare case of almost sterile triploid *C. kyrtostyla*, the species which is commonly diploid. We suppose that this is probably a newly arisen triploid plant with the features of apomictic reproduction;
- Polyploid apomicts form almost exclusively unreduced embryo sac;
- Parthenogenesis is very common in the studied apomicts of *Crataegus* (>90%);
- Rare fertilization of meiotically unreduced egg cell in triploid apomicts is probably confined to certain genotypes and usually carried by a reduced 1x sperm cell;
- Triploids are capable of accepting the pollen with different ploidy levels, 1x, 2x, or even unbalanced ~1.5x, which is almost exclusively confined to fertilization of the central cell;
- Pseudogamy seems to be necessary for the seed formation in polyploid apomicts of *Crataegus* (confirmed in more than 99% of seeds);
- Tetraploids are able to produce rarely meiotically reduced embryo sac and sexual 4x embryos;
- Tetraploids are able to produce sporadically parthenogenetic diploid offspring.

### 4.2. Diploids Reproduce Sexually

As expected, diploids reproduce sexually; reduced embryo sac is double-fertilized almost exclusively by reduced 1x pollen, rarely by unreduced 2x pollen. Sexual reproduction and cross-pollination in diploids of *Crataegus* have been documented [40,45,47,62], and is the rule among diploid angiosperms, only few exceptions were evidenced, e.g., in *Paspalum* L. [90] or *Boechera* Á.Löve et D.Löve [91,92,93]. In Rosaceae family, where *Crataegus* belongs, diploids are mostly sexuals, as observed by FCM in *Amelanchier* [31], *Potentilla* L. [74], *Rubus* L. [73,94], and *Sorbus* [34,76], but few exceptions have been reported [31].

### 4.3. Meiotically Unreduced Embryo Sac Characterizes Polyploids, and Rare Functional Meiosis Occurs More Frequently in Tetraploids than in Triploids

Our analyses revealed that polyploids are practically obligate apomicts with one exception of almost seed-sterile triploid *C. kyrtostyla* (the individual coded kyrtoA). Polyploidy is apparently associated with the loss of developmental success of meiotically reduced embryo sac in *Crataegus*, and only the unreduced putatively aposporic embryo sac develops regularly [45,46]. This is confirmed also in the present study; triploids produced unreduced embryo sac in >99% of seeds, whereas rare meiotically reduced embryo sacs (ca. 9%) have been confirmed in tetraploids. A small sample size of only one tetraploid *C. subsphaerica* investigated here does not allow the generalization of the rare presence of meiosis in tetraploids of *Crataegus*. Similar results were obtained from the open and the experimentally pollinated polyploids of *Crataegus* [40,47,62] and relatives, open-pollinated *Cotoneaster* or *Sorbus* [32,33,34]. Almost total absence of meiotically derived seeds in triploids may be partially reasoned from usual origin of aneuploid embryo in such cases (embryo sac originated after unbalanced meiosis) and its developmental failure.

### 4.4. Rare Fertilization of Egg Cell and Almost Exclusive Fertilization of Central Cell Characterize Polyploids

In polyploid hawthorns, meiotically unreduced egg cell can usually develop parthenogenetically into an embryo. It may be fertilized only rarely and lead to an increase in ploidy level, the origin of 4x–6x embryos, i.e., B_III_ hybrids, together in 6.16% and 1.82% of seeds of all triploid and tetraploid mother plants, respectively. In the present study, potentially viable euploid B_III_ hybrids are well documented among seeds from triploid mother plants by the presence of 4x embryos (53 seeds, 4.54%). Pentaploid B_III_ hybrids (12 seeds, 1.03%) are less frequent, which is contrary to frequent 5x B_III_ hybrids observed earlier [40]. The frequency of ~3–7% of B_III_ hybrids is similar as found in triploids of *Sorbus* (prevalence of 5x B_III_ hybrids [34]) and in tetraploids of *Cotoneaster* (prevalence of 4x and 6x B_III_ hybrids in [33] and [32]). The origin of such 4x–6x hybridogeneous genotypes is of evolutionary importance (see below Section 4.7).

The central cell of embryo sac (usually unreduced) was fertilized in almost all seeds of every investigated polyploid accession. This suggests that pseudogamy is probably necessary for the regular development of seeds. These findings contribute to the general conclusion that in the most cases pollination is required for the seed formation in polyploid *Crataegus* [40,47,62,66,95,96]. This was, for instance, documented also in the related *Cotoneaster* [32,33] or *Sorbus* [34,69,76]. Autonomous endosperm formation in the polyploids of *Crataegus* is therefore lacking or at least very rare [40].

### 4.5. Contrary to Tetraploids, Triploids Are Capable of Utilizing Various Pollen Cytotypes in the Double Fertilization of Central Cells

A high variation in endosperm ploidy level suggests that aside from the pollen with 1x and 2x, reduced pollen from diploids and tetraploids, respectively, even the aneuploid unbalanced ~1.5x pollen (documented by the presence of 3x_emb_/9x_end_ and 3x_emb_/~9.5x_end_ seeds), may be involved in the pollination and fertilization of triploids. Alternative explanation involving unreduced 3x pollen in triploids is improbable because such pollen is at least very rare in triploid *C. subsphaerica* [47], and because of the rarity of 12x endosperm in triploids (6x central cell + 2 × 3x sperm cells), although double fertilization of central cell is quite frequent in hawthorns (documented by the frequent 10x and 12x endosperm in triploids and tetraploids, respectively). Seeds of 3x_emb_/9x_end_ and 3x_emb_/~9.5x_end_ combinations were reported in other genera [24,34,76], and in *Crataegus* by Lo et al. [60], but not by Talent and Dickinson [40]. The latter could be accounted for the small number of investigated cases or controlled pollinations with 1x (from 2x pollen donor) and 2x pollen (from 4x pollen donor), so with the absence of ~1.5x. The observed spectrum of pollen ploidy classes able to fertilize the embryo sac in triploids is opposite to what has been inferred for tetraploid *C. subsphaerica*. It is commonly fertilized only with reduced 2x pollen, and self-compatibility likely can contribute to this observed pattern in this species [47].

We have observed high frequency of 8x, 9x–~9.5x, and 10x endosperm in triploids, and 12x endosperm in tetraploids (Figure 4 and Figure 5). While pollen is likely meiotically reduced in polyploid hawthorns [47], the most abundant endosperm ploidy levels suggest that double fertilization of the central cell is more frequent than fertilization with only one sperm cell, e.g., low frequency of 7x, ~7.5x, and 8x in triploids and 10x in tetraploids. The participation of only one reduced 2x sperm cell instead of two reduced 1x sperm cells in the origin of the most frequent 8x endosperm represents an alternative explanation. However, it seems to be less probable, considering the prevalence of seeds with 9x–~9.5x and 10x endosperm in triploids, and 12x endosperm in tetraploids (for which two sperm-cell fertilization of central cell was unequivocally inferred), and the absence of 10x endosperm in those triploids with high abundance of seeds with 8x endosperm. However, some studies demonstrated that some North American tetraploid hawthorns frequently utilize only one sperm cell in the central cell fertilization [40,62]. These studies suggested that it is a species-specific phenomenon. The predominance of two sperm cells over one sperm cell in the fertilization of central cell is typical in other related genera, *Amelanchier* [31] or *Sorbus* [34,76], but not in *Cotoneaster* [32,33] or *Rubus* [73]. Overall, studies conducted so far on rosaceous genera are inconclusive to this question, but they suggested that plants may differ even within a single genus.

### 4.6. Variation in Endosperm Formation among Triploids Is Related to Pollen Self-/Cross-Compatibility and Inter-/Intracytotypic Compatibility

A large sample size allows us the detection of differences among triploids in the proportion of seed categories (with variable embryo/endosperm ratio) under natural conditions. Triploid individuals were clustered into two groups, namely, FCSSgr1 and FCSSgr2 (Figure 7C). Seeds with 3x_emb_/7x_end_ and 3x_emb_/8x_end_ predominate in FCSSgr1, whereas seeds with 3x_emb_/9x_end_, 3x_emb_/~9.5x_end_, and 3x_emb_/10x_end_ are more frequent in FCSSgr2. To explain the factors underlying this difference, it would be necessary to reveal precisely the ploidy level of the embryo sac, particularly of the egg and the central cell and the sperm cells participating in the fertilization process. However, it is not a trivial task. Resolving this issue using cytoembryological methods is time-consuming, and FCM is limited in the estimation of the number of sperm cells contributing to endosperm [74,97].

Three aspects of breeding system may be considered to explain the observed difference between FCSSgr1 and FCSSgr2: (i) self-incompatibility (conspecific cross-incompatibility) and/or low pollen fertility in some triploids; (ii) intercytotypic pollen incompatibility; and (iii) single-sperm cell vs. two-sperm cell fertilization of central cell. Triploid plants included in FCSSgr1 are likely fertilized by 1x pollen (or less likely 2x pollen) but almost without unbalanced ~1.5x pollen, which can indicate their self-incompatibility or male sterility and their ability to accept only euploid pollen (provided by diploids or less likely by tetraploids, Figure 7). Male sterility was previously reported for some triploids [62]; however, triploid *C. subsphaerica*, the species studied here, produces viable pollen [53]. On the other hand, FCSSgr2 and especially *C. laevigata* × *C. subsphaerica*, with high proportion of the seeds with 9x or ~9.5x endosperm, may differ from FCSSgr1 in the frequent production of unbalanced ~1.5x pollen [47] and con-/non-specific intracytotypic cross-compatibility. This suggests that intercytotypic cross-compatibility may differ among triploids in this study. Previous data have led to the similar conclusion in *Crataegus* [62] or in related rosaceous *Sorbus* [34,69,76]. The trees included in FCSSgr2 also differ in preference to accept 2x pollen (from tetraploids) over 1x pollen (from diploids), which is manifested by the presence of many seeds with 10x endosperm. Most of the plants of FCSSgr2 originated from population with the absence of diploids (or they are at least very rare compared with tetraploids); therefore, the prevalence of 2x over 1x pollen traced in the reconstruction of reproduction modes may be simply due to the rarity of 1x pollen in the population. However, the co-occurrence of tetraploids with triploids of FCSSgr1 on other localities calls against this explanation because it contrasts to the traced absence of 2x pollen in the endosperm origin in the seeds of FCSSgr1 trees. Furthermore, if we suppose double fertilization of central cell in both groups, then we must accept that plants in FCSSgr1 may be almost unable to be fertilized with reduced 2x pollen coming from tetraploids (low frequency of 3x_emb_/10x_end_ seeds).

### 4.7. Evolutionary Implications of the Observed Variation in the Reproduction Pathways in Crataegus

We have demonstrated that the male contribution to the embryo origin is rare, while necessary to the endosperm origin in polyploid hawthorns, and this is crucial for normal seed development. The pollen contribution to an apomictic seed is evolutionarily irrelevant because pollen genes do not get passed on to the next generation. However, if pseudogamy is necessary for regular seed development, it may be relevant what a spectrum of various pollen cytotypes may fertilize central cell. For instance, a plant has better evolutionary chances to prosper if it accepts its own pollen and also the pollen coming from different cytotypes.

The investigated taxa, both the diploids and the polyploids, are morphologically very diverse [35,77], and the presence of some hybrid individuals with intermediate characters supports the role of hybridization in the evolution of *Crataegus* [48]. Mixed-cytotype populations may play an important role in plant diversification. Few triploid offspring descended from diploid mothers more likely in mixed-cytotype populations. They originated from the double fertilization of the reduced embryo sac (1x) by either unreduced 2x sperm cells of diploids or possibly reduced 2x sperm cells of tetraploids. The prevalence of 3x progeny on diploid sexual trees in the mixed-cytotype populations rather than in pure diploid populations is supported by the finding of Hajrudinović et al. [76] in the related genus *Sorbus*. Further, the increase in embryo ploidy level in asexual triploids (the appearance of 4x, ~4.5x, and 5x B_III_ hybrids) is common in mixed-cytotype populations and results from interploid crossings. Similarly, apomictic tetraploids may also rarely form 6x progeny (B_III_ hybrids). The seeds with increased ploidy level compared with the maternal one open the possibility for the origin of new polyploid genotypes [34,40,62,69], which may enhance the evolutionary and taxonomic complexity of the plant group.

We have documented here the plant with decreased fertility, namely, *C. kyrtostyla* (the individual coded kyrtoA). This is the only triploid found with a very low proportion of well-developed seeds (Figure 2), which we interpret as the sign of triploid sterility in otherwise sexual diploid species. The plant, however, produced few seeds, which originated probably asexually. However, the formation of well-developed and fully fertile odd polyploid seeds is generally difficult. One source of these problems is a triploid-block effect, which causes abortion of triploid embryos, particularly in autotriploids, and prevents wide hybridization at the diploid level by requiring balance between parental genomes forming the endosperm genome [66,98]. This block effect involving the developmental failure of the endosperm nutritive tissue is widespread, and it can be modulated with genetic modification [99]. The parental imbalance can produce complete seed failure or abnormal but viable development of endosperm [100] or seeds with reduced seed mass [101]. If any of such newly formed sexual (and sterile) triploid genotypes can produce various proportions of even asexual offspring there is a possibility that some genotypes can be purely asexual [66]. It remains questionable if the appearance of such asexual genotypes could lead to the establishment of a new apomictic lineage. A selection may, however, act in favor of such asexual genotypes [10] and establish them as microspecies through apomictic reproduction. Asexual hawthorns studied here expressed relaxation in strict balanced maternal (m) to paternal (p) 2m:1p contributions in the endosperm origin. The high prevalence of unreduced embryo sacs in polyploids has been documented, but only 9x endosperm (maybe even ~8.5x and ~9.5x, together 24.91%) in triploids and 12x endosperm (maybe even ~11.5x and ~12.5x, together 66.36%) in tetraploids have surely obtained the 2m:1p parental contributions to the endosperm. The rest of the seeds contain the endosperm with unbalanced parental ratio. This intriguing tolerance pattern was previously documented in North American hawthorns [40], as well as other related genera, *Amelanchier* [31], *Cotoneaster* [32,33], and *Sorbus* [34,76]. Relaxed or removed endosperm-balance requirement is considered necessary for the evolution of pseudogamous apomixis and would further account for the crossing ability of diploids and polyploids [66].

## 5. Conclusions

This is the first quantitatively evaluated FCSS study of reproduction modes in diploid–polyploid hawthorn populations with a high proportion of triploids. Extensive variation in the embryo and endosperm origins was documented. As expected, diploids are fully sexual and can be pollinated predominantly only with reduced 1x pollen of diploids. Triploids are obligate apomicts, and a spectrum of pollen ploidy classes, euploid or aneuploid, participate in their central cell fertilization. The only tetraploid representative, *C. subsphaerica*, is facultative asexual species with residual sexuality. It is pollinated almost exclusively with reduced 2x pollen. Triploids of *Crataegus* differ in how both embryo and endosperm are formed and which of the reduced 1x, ~1.5x, or 2x pollen fertilizations of embryo sac occur more frequently, which may stem from differences in pollen self- and cross-compatibility and intra- and intercytotypic compatibility. Overall, seed development is apparently successful in triploid *Crataegus* for all pollen cytotypes contributing to the seed origin. We observed remarkable complexity within the hawthorn populations, which nominates this plant group as a potential candidate for further research of cytotypic interactions in sexual–apomictic populations.

## Figures and Tables

**Table 1:** Cytotypic diversity in 15 populations of *Crataegus* in eastern Slovakia irrespective of taxa affiliation [78]. Populations marked with asterisks indicate the source populations for triploid mother trees investigated using the FCSS. Populations were selected to sample various morphologically diverse taxa, especially triploids, and to include those accessible individuals that produced a considerable number of fruits available for collection.

Population	Code	Number of Plants	Proportion of Cytotypes [%]		
			2x	3x	4x
Hermanovce *	HERM	47	0.00	34.04	65.96
Lipany	LIP	40	100.00	0.00	0.00
Košice, Čičky *	CIC	28	35.71	46.43	17.86
Čierna nad Tisou	C	18	100.00	0.00	0.00
Malý Kamenec	MK	18	77.78	22.22	0.00
Leles	LL	11	90.91	9.09	0.00
Hermanovce, Gočalka	GOC	10	30.00	20.00	50.00
Košice, Botanical Garden	BOTZ	9	88.89	11.11	0.00
Ražňany *	RAZNH	9	33.33	22.22	44.44
Košice, Kavečany *	KAV	8	12.50	25.00	62.50
Biel	BIEL	7	100.00	0.00	0.00
Uzovské Pekľany	MAJ	2	0.00	0.00	100.00
Dobrá	DOBRA	1	100.00	0.00	0.00
Prešov	PO	1	100.00	0.00	0.00
Košice, UVL	KO	1	100.00	0.00	0.00
SUM	15	210			

**Table 2:** Survey of plant material of the genus *Crataegus* used in the study. Taxonomic assignment to a particular species and hybrids is given in the *Taxon* column; the inferred ploidy level, in the *Ploidy level* column; the source population, in the *Locality* column; and particular accessions, in the *Plant code* column. The sample size used in analyses is provided for fruits precisely investigated for successful development of seeds (*Fruits*), seeds analyzed for the FCSS (*FCSS seeds*), with successful determinations of both embryo and endosperm ploidy level (*FCSS success*). NA—not applied. Those plants marked with asterisks indicate the individuals with indirectly determined ploidy level—inferred from the ploidy level of their progeny.

Taxon	Ploidy Level	Locality	Plant Code	Fruits	FCSS Seeds	FCSS Success
*C. monogyna*	2x	MK	MKT	22	10	10
	2x	CIC	MONOG1	29	20	18
	2x	CIC	MONOG3	28	20	20
	2x	CIC	KVP4	26	20	20
	2x	BOTZ	BOTZ1	23	20	20
	2x	BOTZ	BOTZ2	NA	10	10
	2x	KO	KO VETER	NA	10	10
	2x	BIEL	BIEL15-3	NA	10	10
	2x	DOBRA	DOBRA1	NA	10	10
	2x	C	CIERNANT1	NA	10	7
*C. kyrtostyla*	2x	PO	PO1	31	20	18
	2x	CIC	KVP1	33	10	10
	2x	GOC	GOCAL15-1	12	10	10
	2x	GOC	GOCAL15-2	14	10	9
	2x	GOC	GOCAL15-3	11	10	10
	2x	LIP	LIP15-1	32	20	20
	2x	LIP	LIP15-2	28	20	20
	2x	LIP	LIP15-5	15	NA	NA
	2x	RAZNH	RAZNH15-1	15	10	10
	2x	RAZNH	RAZNH15-2	NA	8	5
Diploids SUM			20	319	258	247
*C. kyrtostyla*	3x	CIC	1518/11	58	29	29
	3x	KAV	kyrtoA	121	5	5
*C. rhipidophylla*	3x	CIC	1447/11	76	5	5
	3x	CIC	1453/11	58	47	46
*C. subsphaerica*	3x	HERM	Vyhon3	198	98	97
	3x	CIC	1445/11	88	18	18
	3x	CIC	1472/11	103	61	59
	3x	CIC	1478/11	59	44	44
	3x	CIC	1531/11	80	34	34
	3x	CIC	1537/11	62	54	52
	3x	CIC	1539/11	70	65	63
	3x	CIC	1521/11	32	20	19
	3x	CIC	1525/11	54	31	31
	3x	CIC	1527/11	105	88	88
*C. laevigata* × *C. kyrtostyla*	3x	HERM	Hurka1	239	114	110
*C. kyrtostyla* × *C. subsphaerica*	3x	RAZNH	RaznH2	121	104	100
*C. subsphaerica* × *C. laevigata*	3x	RAZNH	RaznH1	117	100	95
*C. laevigata* × *C. subsphaerica*	3x	HERM	Sosen3	140	135	133
	3x	HERM	Vyhon4	215	73	67
	3x	HERM	Vyhon8	87	77	73
Triploids SUM			20	2083	1202	1168
*C. subsphaerica*	4x	HERM	PS-Vyh16-1	NA	5	5
	4x	HERM	PS-Vyh16-2	NA	8	7
	4x	HERM	Vyhon2	NA	8	8
	4x *	HERM	Vyhon16-16	NA	10	10
	4x	HERM	Vyhon16-15	NA	17	16
	4x *	RAZNH	RaznH16-3	NA	15	15
	4x	MAJ	MAJ16-1	NA	27	27
	4x	MAJ	MAJ16-2	NA	24	22
Tetraploids SUM			8	NA	114	110
Total SUM			48	2402	1574	1525

**Table 3:** Seed categories in *Crataegus*, which are described as follows: Line 1, embryo ploidy/endosperm ploidy; Line 2, number of seeds; percentage calculated from all analyzed seeds with the concerned maternal ploidy; Line 3, reproduction mode—embryo formation (S, sexual; P, parthenogenetic; B_III_, unreduced egg cell fertilized; rP, reduced parthenogenesis)/mode of endosperm formation (aute, autonomous endosperm; ep, endopolyploidization; ♂, ploidy of sperm cell contributing to endosperm formation; pn, polynucleated central cell; poly, polyspermy). Note that ~1.5x ploidy level depicts highly unbalanced aneuploid reduced pollen of 3x plants, possibly of a range between 1x and 2x.

Maternal Plant Ploidy	Seed Category				
2x	2x_emb_/3x_end_ (240; 97.17%) S/ 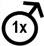	3x_emb_/4x_end_ (5; 2.02%) S/ 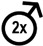 *^a^			2x_emb_/6x_end_ (2; 0.81%) S/ 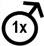 ep
3x	3x_emb_/8x_end_ (457; 38.13%) P/ 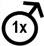 + 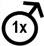 or 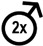	3x_emb_/~8.5x–9x–~9.5x_end_ *^b^ (289; 24.74%) P/ 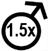 + 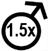			3x_emb_/10x_end_ (136; 11.64%) P/ 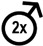 + 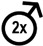
	3x_emb_/7x_end_ (83; 7.11%) P/ 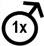	3x_emb_/~7.5x_end_ (54; 4.62%) P/ 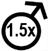			3x_emb_/6x–~6.5x_end_ (4; 0.34%) P/aute
	3x_emb_/~>10x_end_ *^c^ (61; 5.22%) P/ep or pn			~3.5x_emb_/9x–12x_end_ *^d^ (3; 0.26%)	
	4x_emb_/7x_end_ (31; 2.65%) S(B_III_)/ 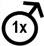	4–~4.5x_emb_/~6.5x–~7.5x_end_ (9; 0.77%) S(B_III_)/ 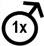 or 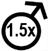			5x_emb_/8x_end_ (5; 0.43%) S(B_III_)/ 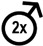
	4x, 5x_emb_/8x, 10x_end_ (27; 2.31%) S(B_III_)/ 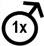 or 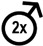 , poly			6x_emb_/~≥14x_end_ (6; 0.51%) P/ep	
4x	4x_emb_/12x_end_ (56; 50.91%) P/ 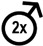 + 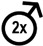	4x_emb_/~11.5x, ~12.5x_end_ *^e^ (17; 15.45%) P/ 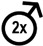 + 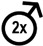			4x_emb_/10x–~10.5x_end_ *^f^ (9; 8.18%) P/ 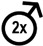
	4x_emb_/~>12.5x_end_ *^c^ (14; 12.73%) P/ 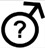 + 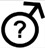 or aute + ep or pn?	4x_emb_/~10.5x–11x_end_ *^g^ (2; 1.82%) P/ 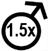 + 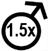			2x_emb_/8x–~9.5x_end_ *^h^ (6; 5.45%) rP/ 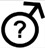 + 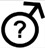 or aute + ep or pn?
	4x_emb_/6x_end_ (4; 3.64%) S/ 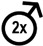		6x_emb_/10x_end_ (2; 1.82%) S(B_III_)/ 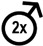		

*^a^ the reduced 2x pollen from 4x plants or the unreduced 2x pollen from 2x plants. *^b^ the measured ~9.5x endosperms could indicate double fertilization of the central cell by aneuploid pollen higher than 1.5x (but 1.5x < 2x). *^c^ unresolved contribution of sperm cells; the cases here may involve even trinucleate central cell, endopolyploidy or contribution of sperm cells with >2x ploidy level. *^d^ unresolved contribution of sperm cells. *^e^ the measured ~11.5x and ~12.5x endosperms could be considered the 12x endosperms here because of potential measurement error. *^f^ the measured ~10.5x endosperm could be considered the 10x endosperm here because of potential measurement error. *^g^ the measured ~10.5x endosperm could be considered the 11x endosperm here because of potential measurement error. *^h^ the measured ~9.5x endosperm could be considered as the result of fertilization of potentially trinucleate central cell.

## Data Availability

Flow cytometric seed screen data are presented in Supplementary Material Table S1.

## Supplementary Materials

The following supporting information can be downloaded at: https://www.mdpi.com/article/10.3390/plants11243497/s1, Figure S1: Method of mechanical isolation of seed from pyrene; Table S1: Original flow cytometric seed screen (FCSS) data; Note S1: Origin of plant material used in the present study; Note S2: Calculations of the ploidy level of the embryo and endosperm and sperm cells which fertilized egg or central cell.
