# Supplementary material for: Variability of Reproduction Pathways in the Central-European Populations of Hawthorns with Emphasis on Triploids
Source: Plants (Basel). 2022 Dec 13;11(24):3497. doi: 10.3390/plants11243497 (PMC9786806; doi:10.3390/plants11243497)
Supplement: Supplementary file 1 [file plants-11-03497-s001.zip › Supplementary Material Kolarcik et al Plants/Supplementary Material Note S1 Figure S1 Note S2.pdf]

Supplementary Material

Article Title: Variability of reproduction pathways in the Central-European populations of hawthorns with emphasis on triploids.

Authors: Vladislav Kolarčik[1,*], Valéria Kocová[2], Vlastimil Mikoláš[3], Lenka Mártonfiová[2], Nikola Hajdučeková[4] and Pavol Mártonfi[1, 2]

[1] Institute of Biology and Ecology, Faculty of Science, Pavol Jozef Šafárik University, Mánesova 23, SK-041 54, Košice, Slovak republic;

[2] Botanical Garden, Pavol Jozef Šafárik University, Mánesova 23, SK-043 52 Košice, Slovak republic;

[3] Hanojská 4, SK-040 13 Košice, Slovak Republic;

and

[4] Buzulucká 5, SK-040 22 Košice, Slovak Republic;

Note S1 Origin of plant material used in the present study.

Hermanovce - Prešov district, Hermanovce, forest margins NW of the village, approx. 49°03’06.8”, 21°00’22.5”

Uzovské Pekľany - Sabinov district, Uzovské Pekľany, forest margins ca. 1 km S of the village, approx. 49°04’34.2”, 21°01’08.9”

Lipany - Sabinov district, Lipany, W margin of the city, along the agrarian road close to cemetery, 49°09’26.9”, 20°57’31.7”

Košice, Čičky - Košice district, Košice, margins of agrarian area W of the city, local name Čičky, 48°43’22.8”, 21°12’44.3”

Čierna nad Tisou - Trebišov district, Čierna nad Tisou, along the road from Čierna nad Tisou to the village of Čierna, 48°25’30.2”, 22°06’39.3”

Malý Kamenec - Trebišov district, Malý Kamenec, vineyards E of the village, 48°21’24.9”, 21°47’02.3”

Leles - Trebišov district, Leles, along the road between the village of Leles to the town of Veľké Kapušany, approx. 48°30’49.6”, 22°03’13.4”

Hermanovce, Gočalka - Prešov district, Hermanovce, forest margins SE of village, local name Gočalka, 49°02’31.7”, 21°01’15.3”

Košice, Botanical Garden - Košice district, Košice, arboretum of the Botanical Garden in Košice, 48°44’09.3”, 21°14’08.6”

Ražňany - Sabinov district, Ražňany, along the road from the village of Jarovnice to the village of Ražňany and adjacent forest margins, 49°04’17.3”, 21°04’34.4”

Košice, Kavečany - Košice district, Kavečany, N of the village, close to ZOO park, approx. 48°47’34.0”, 21°11’54.2”

Biel - Trebišov district, Biel, along the road to the village of Veľké Trakany, approx. 48°24’16.1”, 22°03’30.3”

Dobrá - Trebišov district, Dobrá, S margin of the village, close to bridge above railway, 48°24’27.7”, 22°01’33.9”

Prešov - Prešov district, Prešov, along the motorway to Vranov nad Topľou, eastern edge of the public cemetery in Prešov, 49°00’19.1”, 21°14’45.3”

Košice, UVL - Košice district, Košice, areal of the University of Veterinary Medicine and Pharmacy in Košice, 48°44’27.6”, 21°14’49.8”

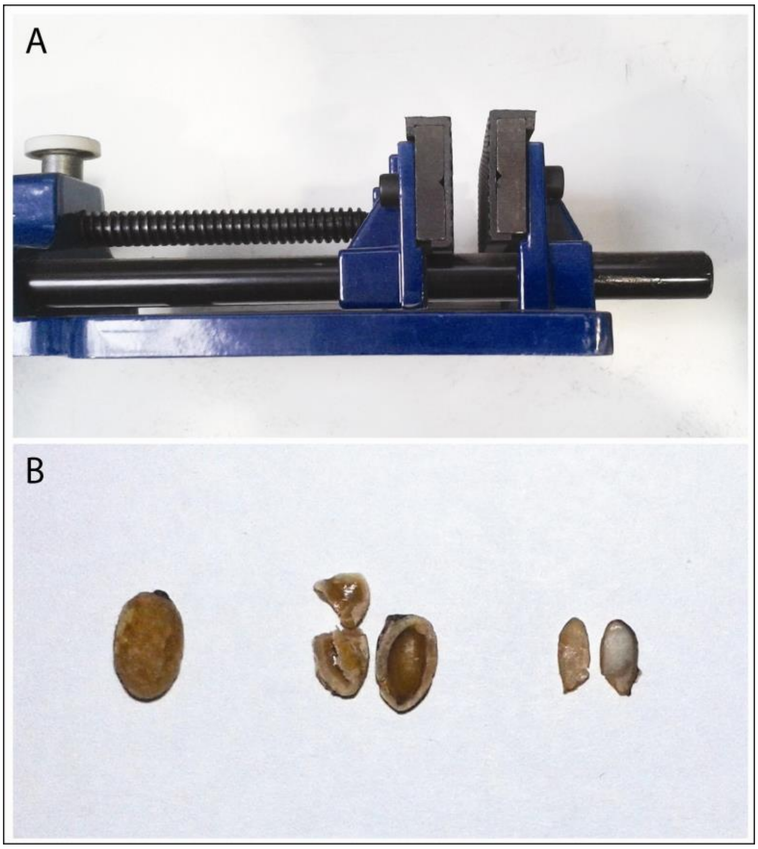

Figure S1 Method of mechanical isolation of seed from pyrene, A) vise device was used to open pyrene; B) from left to right, examples of whole pyrene, open pyrene with seed visible on right half of pyrene, and halved seed.

Note S2 Calculations of the ploidy level of the embryo and endosperm and sperm cells which fertilized egg or central cell

DNA content of embryo or endosperm was determined according to the formulas:

$G_{emb} = G_{IRS} \times F_{emb} / F_{IRS}$

$G_{end} = G_{IRS} \times F_{end} / F_{IRS}$

Or in the cases where endosperm DNA content was based on two separate measurement of only embryo+endosperm and embryo+IRS (internal reference standard) analyses, the formula was used:

$G_{end} = G_{emb} \times F_{end} / F_{emb}$

where

G_{emb} – DNA amount of embryo

G_{end} – DNA amount of endosperm

G_{IRS} – DNA amount of internal reference standard

F_{emb} – fluorescence G_0/G_1 peak mean of embryo

F_{end} – fluorescence G_0/G_1 peak mean of endosperm

F_{IRS} – fluorescence G_0/G_1 peak mean of internal reference standard

Example of calculations are here given for triploids. In most cases our analyses found genome size of embryo and endosperm in the ranges corresponding to 3x embryo and > 6x endosperm, which is very consistent with interpretation of the origin of these embryos as a result of parthenogenetic development of unreduced egg cell (see below). According to theoretical assumptions genome size of such embryos should be the same as the genome size of their mother tree. Therefore, we compared mean genome size of 3x parthenogenetic embryos and their mother trees to investigate expected genome size equality. We observed linear relationship and significant correlation between genome size of mother trees and mean genome size of their 3x parthenogenetic embryos. The relationship also suggests genome size variation among mother trees and also among 3x embryo families. This led us to calculate ploidy level of embryo with respect to mean genome size of the respective seed family.

Hence the mean genome size value of the respective 3x embryo family was used for estimation of embryo and endosperm ploidy level according to formulas:

$$P_{emb} = 3 \times G_{emb} / G_{triploid}$$

$$P_{end} = P_{emb} \times G_{end} / G_{emb}$$

where

P_{emb} – calculated ploidy level of embryo

P_{end} – calculated ploidy level of endosperm

3 – expected ploidy level of 3x embryo family

$G_{triploid}$ – mean genome size of respective parthenogenetic 3x embryo family

G_{emb} and G_{end} as above

Reproduction pathways were than interpreted based on embryo and endosperm ploidy level data according to Matzk et al. (2000, 2001).

We reckon with possible variation in ploidy level calculation, which results into noninteger number and therefore an approximation was applied. If ploidy level was calculated and falls e.g., within the range X.75-Y.249 (e.g., 1.75-2.249) we accept value Yx (e.g., 2x) as approximated ploidy level. If the range was X.25-X.749 (e.g., 2.25-2.749) we accept X.5x (e.g., 2.5x) as approximated ploidy level. Approximated ploidy levels were used to assign seeds to seed categories (see Table 3)

Mean 1Cx DNA content was calculated for each seed family (seeds of single mother tree):

$C_{triploid} = G_{triploid} / 3$

where

$C_{triploid}$ – mean 1Cx DNA content of the respective seed family

$G_{triploid}$ as above

DNA amount of central cell for each seed was calculated. We suppose 6x or 12x central cell after inspecting embryo and endosperm ploidy level which suggests that in some cases embryo sacs with endoreduplicated genome could occur:

$G_{ccell} = 6 \times G_{emb} / P_{emb}$

$G_{ccell} = 12 \times G_{emb} / P_{emb}$

where

G_{ccell} – DNA amount of central cell

6 – expected ploidy level of 6x central cell

12 – expected ploidy level of 12x central cell

G_{emb} and P_{emb} as above

We further estimate genome size and ploidy level for sperm cell/s contributing to embryo and endosperm genome:

$S_{emb} = P_{emb} – 3 \times G_{emb} / P_{emb}$

$S_{emb} = P_{emb} - 6 \times G_{emb} / P_{emb}$

$S_{end} = G_{end} - G_{ccell}$

$L_{emb} = S_{emb} / C_{triploid}$

$L_{end} = S_{end} / C_{triploid}$

where

S_{emb} – DNA amount of contributed sperm cell/s to embryo

S_{end} – DNA amount of contributed sperm cell/s to endosperm

L_{emb} – ploidy level of contributed sperm cell/s to embryo

L_{end} – ploidy level of contributed sperm cell/s to endosperm

3 – expected ploidy level of 3x egg cell of unreduced embryo sacs

6 – expected ploidy level of 6x egg cell of unreduced embryo sacs with endoreduplicated genome

G_{emb}, G_{end}, G_{ccell}, P_{emb} and $C_{triploid}$ as above
