# Supplementary material for: Variability of Reproduction Pathways in the Central-European Populations of Hawthorns with Emphasis on Triploids
Source: Plants (Basel). 2022 Dec 13;11(24):3497. doi: 10.3390/plants11243497 (PMC9786806; doi:10.3390/plants11243497)
Supplement: Supplementary file 1 [file plants-11-03497-s001.zip › Supplementary Material Kolarcik et al Plants/Supplementary Material Table S1.pdf]

Supplementary Material Table S1 Original flow cytometric seed screen (FCSS) data. Mother plants and number of seeds used in FCM sample is given followed by DNA content of embryo and endosperm, and calculated and estimated ploidy level for both embryo and endosperm. Triploid plants are differentiated into two groups based on reproduction modes.

2x plants

plant	seeds	embryo	endosperm	ploidy_emb_calc	ploidy_end_calc	emb_level_est	end_level_est	group
BIEL15-3	5	1.5208	2.2751	1.9879	2.9739	2.0	3.0	x
BIEL15-3	5	1.5393	2.3099	2.0121	3.0193	2.0	3.0	x
BOTZ1	1	1.5833	2.3478	1.9995	2.9651	2.0	3.0	x
BOTZ1	1	1.5892	2.3418	2.0070	2.9574	2.0	3.0	x
BOTZ1	1	1.6032	2.3771	2.0246	3.0020	2.0	3.0	x
BOTZ1	1	1.5753	2.3555	1.9895	2.9748	2.0	3.0	x
BOTZ1	1	1.5659	2.3359	1.9776	2.9500	2.0	3.0	x
BOTZ1	1	1.5622	2.3387	1.9729	2.9536	2.0	3.0	x
BOTZ1	1	1.5520	2.3222	1.9600	2.9327	2.0	3.0	x
BOTZ1	1	1.5675	2.3305	1.9796	2.9432	2.0	3.0	x
BOTZ1	1	1.5597	2.3188	1.9697	2.9285	2.0	3.0	x
BOTZ1	1	1.6031	2.4380	2.0245	3.0790	2.0	3.0	x
BOTZ1	5	1.6416	2.4560	2.0732	3.1017	2.0	3.0	x
BOTZ1	5	1.6009	2.3860	2.0218	3.0133	2.0	3.0	x
BOTZ2	1	1.5753	2.3361	1.9990	2.9644	2.0	3.0	x
BOTZ2	1	1.5932	2.3673	2.0217	3.0040	2.0	3.0	x
BOTZ2	1	1.5833	2.3568	2.0091	2.9907	2.0	3.0	x
BOTZ2	1	1.5873	2.3442	2.0142	2.9747	2.0	3.0	x
BOTZ2	1	1.5833	2.3478	2.0091	2.9793	2.0	3.0	x
BOTZ2	5	1.5342	2.3035	1.9468	2.9230	2.0	3.0	x
CIERNAnT1	1	1.7691	2.4262	2.0179	2.7674	2.0	3.0	x
CIERNAnT1	1	1.7691	2.6677	2.0179	3.0428	2.0	3.0	x
CIERNAnT1	1	1.7691	2.6277	2.0179	2.9972	2.0	3.0	x
CIERNAnT1	1	1.7691	2.5339	2.0179	2.8903	2.0	3.0	x
CIERNAnT1	1	1.7691	2.5890	2.0179	2.9530	2.0	3.0	x

CIERNAnT1	1	1.7142	2.4628	1.9553	2.8091	2.0	3.0	x
CIERNAnT1	1	1.7142	2.6489	1.9553	3.0214	2.0	3.0	x
KVP1	5	1.6416	2.4497	2.0031	2.9891	2.0	3.0	x
KVP1	5	1.6723	2.4994	2.0405	3.0497	2.0	3.0	x
KVP4	5	1.6231	2.4171	1.9804	2.9493	2.0	3.0	x
KVP4	5	1.6271	2.4356	1.9853	2.9718	2.0	3.0	x
KVP4	5	1.6024	2.3965	1.9552	2.9241	2.0	3.0	x
KVP4	5	1.6682	2.4843	2.0355	3.0313	2.0	3.0	x
LIP15-1	5	1.6262	2.4012	1.9621	2.8972	2.0	3.0	x
LIP15-1	5	1.6199	2.3911	1.9545	2.8851	2.0	3.0	x
LIP15-1	5	1.7148	2.5292	2.0691	3.0517	2.0	3.0	x
LIP15-1	5	1.6694	2.4500	2.0143	2.9561	2.0	3.0	x
LIP15-2	5	1.5903	2.3649	1.9970	2.9697	2.0	3.0	x
LIP15-2	5	1.5802	2.3566	1.9844	2.9593	2.0	3.0	x
LIP15-2	5	1.5793	2.3746	1.9832	2.9818	2.0	3.0	x
LIP15-2	5	1.6209	2.4023	2.0354	3.0166	2.0	3.0	x
MKT	1	1.5578	2.3159	1.9660	2.9227	2.0	3.0	x
MKT	1	1.5985	2.3433	2.0173	2.9574	2.0	3.0	x
MKT	1	1.5752	2.3419	1.9879	2.9556	2.0	3.0	x
MKT	1	1.5749	2.3426	1.9876	2.9565	2.0	3.0	x
MKT	1	1.5974	2.3851	2.0160	3.0100	2.0	3.0	x
MKT	1	1.6017	2.3762	2.0214	2.9989	2.0	3.0	x
MKT	1	1.5724	2.3567	1.9844	2.9743	2.0	3.0	x
MKT	1	1.5606	2.3287	1.9695	2.9389	2.0	3.0	x
MKT	1	1.5855	2.3645	2.0010	2.9841	2.0	3.0	x
MKT	1	1.6236	2.4126	2.0490	3.0448	2.0	3.0	x
PO1	1	1.5852	2.3662	2.0540	3.0659	2.0	3.0	x
PO1	1	1.5692	2.3323	2.0332	3.0220	2.0	3.0	x
PO1	1	1.5873	2.3541	2.0567	3.0503	2.0	3.0	x
PO1	1	1.5733	2.3284	2.0386	3.0169	2.0	3.0	x
PO1	1	1.5365	2.2946	1.9908	2.9732	2.0	3.0	x
PO1	1	1.5424	2.3106	1.9985	2.9940	2.0	3.0	x
PO1	1	1.5465	2.3098	2.0038	2.9928	2.0	3.0	x

PO1	1	1.5338	2.2906	1.9874	2.9680	2.0	3.0	x
PO1	1	1.5055	2.2701	1.9507	2.9414	2.0	3.0	x
PO1	1	1.5077	2.2568	1.9535	2.9242	2.0	3.0	x
PO1	1	1.5215	2.2874	1.9714	2.9638	2.0	3.0	x
PO1	1	1.5371	2.2918	1.9916	2.9696	2.0	3.0	x
PO1	1	1.5355	2.3000	1.9896	2.9802	2.0	3.0	x
PO1	1	1.5373	2.2935	1.9920	2.9717	2.0	3.0	x
PO1	1	1.5495	2.3171	2.0077	3.0024	2.0	3.0	x
PO1	1	1.5339	2.3063	1.9876	2.9883	2.0	3.0	x
PO1	1	1.5400	2.2898	1.9954	2.9669	2.0	3.0	x
PO1	1	1.5418	2.3054	1.9977	2.9872	2.0	3.0	x
DOBRA1	1	1.6608	2.4760	1.9377	2.8888	2.0	3.0	x
DOBRA1	1	1.7042	2.4465	1.9883	2.8543	2.0	3.0	x
DOBRA1	1	1.7299	2.4975	2.0183	2.9139	2.0	3.0	x
DOBRA1	1	1.6539	2.3813	1.9296	2.7783	2.0	3.0	x
DOBRA1	1	1.6709	2.4367	1.9495	2.8429	2.0	3.0	x
DOBRA1	1	1.8606	2.6593	2.1708	3.1027	2.0	3.0	x
DOBRA1	1	1.7155	2.6195	2.0014	3.0562	2.0	3.0	x
DOBRA1	1	1.7155	2.5258	2.0014	2.9469	2.0	3.0	x
DOBRA1	1	1.7155	2.5257	2.0014	2.9467	2.0	3.0	x
DOBRA1	1	1.7155	2.4870	2.0014	2.9016	2.0	3.0	x
GOCAL15-1	1	1.5731	2.3530	2.0058	3.0002	2.0	3.0	x
GOCAL15-1	1	1.5644	2.3468	1.9947	2.9923	2.0	3.0	x
GOCAL15-1	1	1.5700	2.3413	2.0018	2.9853	2.0	3.0	x
GOCAL15-1	1	1.5736	2.3378	2.0064	2.9808	2.0	3.0	x
GOCAL15-1	1	1.5731	2.3382	2.0058	2.9814	2.0	3.0	x
GOCAL15-1	1	1.5731	2.3530	2.0058	3.0002	2.0	3.0	x
GOCAL15-1	1	1.5700	2.3413	2.0018	2.9853	2.0	3.0	x
GOCAL15-1	1	1.5644	2.3322	1.9947	2.9737	2.0	3.0	x
GOCAL15-1	1	1.5481	2.3022	1.9739	2.9354	2.0	3.0	x
GOCAL15-1	1	1.5759	2.3356	2.0093	2.9779	2.0	3.0	x
GOCAL15-2	1	1.5265	2.2690	1.9732	2.9328	2.0	3.0	x
GOCAL15-2	1	1.5706	2.3120	2.0301	2.9885	2.0	3.0	x

GOCAL15-2	1	1.5084	2.2570	1.9497	2.9173	2.0	3.0	x
GOCAL15-2	1	1.5675	2.3148	2.0261	2.9921	2.0	3.0	x
GOCAL15-2	1	2.1865	2.9590	2.8262	3.8248	3.0	4.0	x
GOCAL15-2	1	1.5675	2.3148	2.0261	2.9921	2.0	3.0	x
GOCAL15-2	1	1.5822	2.3294	2.0451	3.0110	2.0	3.0	x
GOCAL15-2	1	1.5515	2.3144	2.0054	2.9915	2.0	3.0	x
GOCAL15-2	1	1.5042	2.2500	1.9444	2.9083	2.0	3.0	x
GOCAL15-3	1	1.5612	2.3205	1.9934	2.9629	2.0	3.0	x
GOCAL15-3	1	1.5515	2.3144	1.9809	2.9551	2.0	3.0	x
GOCAL15-3	1	1.5495	2.3177	1.9784	2.9593	2.0	3.0	x
GOCAL15-3	1	1.5644	2.3177	1.9975	2.9593	2.0	3.0	x
GOCAL15-3	1	1.5162	2.2765	1.9359	2.9067	2.0	3.0	x
GOCAL15-3	1	1.6670	2.4003	2.1285	3.0647	2.0	3.0	x
GOCAL15-3	1	1.5668	2.3294	2.0006	2.9743	2.0	3.0	x
GOCAL15-3	1	1.5679	2.3387	2.0020	2.9861	2.0	3.0	x
GOCAL15-3	1	1.5580	2.3234	1.9893	2.9666	2.0	3.0	x
GOCAL15-3	1	1.5612	2.3352	1.9934	2.9817	2.0	3.0	x
KO VETER	5	1.5516	2.3067	1.9469	2.8945	2.0	3.0	x
KO VETER	5	1.6362	2.4319	2.0531	3.0516	2.0	3.0	x
MONOGYNA1	5	1.7108	2.5521	2.1052	3.1405	2.0	3.0	x
MONOGYNA1	5	1.6036	2.3685	1.9733	2.9145	2.0	3.0	x
MONOGYNA1	1	1.6200	2.3996	1.9934	2.9528	2.0	3.0	x
MONOGYNA1	1	1.6056	2.3787	1.9757	2.9270	2.0	3.0	x
MONOGYNA1	1	1.6059	2.3800	1.9761	2.9287	2.0	3.0	x
MONOGYNA1	5	1.6060	2.4031	1.9763	2.9571	2.0	3.0	x
MONOG3	5	1.5897	2.3398	1.9811	2.9160	2.0	3.0	x
MONOG3	1	1.5574	2.3232	1.9408	2.8952	2.0	3.0	x
MONOG3	1	1.6551	2.5601	2.0627	3.1904	2.0	3.0	x
MONOG3	1	1.5845	4.9141	1.9746	6.1241	2.0	6.0	x
MONOG3	1	1.5772	2.2700	1.9655	2.8290	2.0	3.0	x
MONOG3	1	1.5915	4.8085	1.9833	5.9925	2.0	6.0	x
MONOG3	5	1.6527	2.4654	2.0596	3.0724	2.0	3.0	x
MONOG3	5	1.6307	2.4159	2.0323	3.0108	2.0	3.0	x

RAZNH15-1	1	2.3356	3.1599	2.9755	4.0257	3.0	4.0	x
RAZNH15-1	1	1.5617	2.3233	1.9896	2.9599	2.0	3.0	x
RAZNH15-1	1	2.3745	3.1860	3.0251	4.0590	3.0	4.0	x
RAZNH15-1	1	1.5727	2.3387	2.0036	2.9795	2.0	3.0	x
RAZNH15-1	1	1.5548	2.3264	1.9808	2.9639	2.0	3.0	x
RAZNH15-1	1	1.5762	2.3646	2.0081	3.0125	2.0	3.0	x
RAZNH15-1	1	1.5736	2.3378	2.0048	2.9784	2.0	3.0	x
RAZNH15-1	1	1.5793	2.3468	2.0120	2.9899	2.0	3.0	x
RAZNH15-1	1	1.5762	2.3499	2.0081	2.9938	2.0	3.0	x
RAZNH15-1	1	1.5644	2.3468	1.9930	2.9899	2.0	3.0	x
Raznh15-2	1	1.5823	2.3148	2.0484	2.9968	2.0	3.0	x
Raznh15-2	1	2.2156	3.0028	2.8684	3.8874	3.0	4.0	x
Raznh15-2	1	2.2337	3.0028	2.8917	3.8874	3.0	4.0	x
Raznh15-2	1	1.5412	2.3120	1.9953	2.9932	2.0	3.0	x
Raznh15-2	1	1.5111	2.2316	1.9563	2.8890	2.0	3.0	x

3x plants

plant	seeds	embryo	endosperm	ploidy_emb_ca	ploidy_end_cal	emb_level_est	end_level_est	group
1445-11	1	2.3553	5.5470	2.9795	7.0171	3.0	7.0	FCSSgr1
1445-11	1	2.3570	5.5060	2.9817	6.9652	3.0	7.0	FCSSgr1
1445-11	1	2.3475	6.2978	2.9697	7.9669	3.0	8.0	FCSSgr1
1445-11	1	2.3503	6.2187	2.9731	7.8667	3.0	8.0	FCSSgr1
1445-11	1	2.3503	6.3248	2.9732	8.0011	3.0	8.0	FCSSgr1
1445-11	1	2.3516	6.2763	2.9748	7.9397	3.0	8.0	FCSSgr1
1445-11	1	2.3523	6.3734	2.9757	8.0625	3.0	8.0	FCSSgr1
1445-11	1	2.3604	6.3938	2.9860	8.0883	3.0	8.0	FCSSgr1
1445-11	1	2.3645	6.3435	2.9911	8.0246	3.0	8.0	FCSSgr1
1445-11	1	2.3660	6.3714	2.9930	8.0600	3.0	8.0	FCSSgr1
1445-11	1	2.3691	6.3452	2.9969	8.0268	3.0	8.0	FCSSgr1
1445-11	1	2.3723	6.3087	3.0011	7.9806	3.0	8.0	FCSSgr1
1445-11	1	2.3817	6.3197	3.0129	7.9945	3.0	8.0	FCSSgr1
1445-11	1	2.3856	6.2830	3.0179	7.9481	3.0	8.0	FCSSgr1

1445-11	1	2.4237	6.4011	3.0660	8.0975	3.0	8.0	FCSSgr1
1445-11	1	2.4838	6.5571	3.1421	8.2949	3.0	8.5	FCSSgr1
1445-11	1	2.3578	7.4341	2.9827	9.4043	3.0	9.5	FCSSgr1
1445-11	1	2.3578	10.9592	2.9826	13.8636	3.0	14.0	FCSSgr1
1447-11	1	2.3090	6.2355	2.9654	8.0081	3.0	8.0	FCSSgr1
1447-11	1	2.3243	6.2498	2.9850	8.0264	3.0	8.0	FCSSgr1
1447-11	1	2.3389	6.3288	3.0038	8.1279	3.0	8.0	FCSSgr1
1447-11	1	2.3459	6.2526	3.0128	8.0301	3.0	8.0	FCSSgr1
1447-11	1	2.3617	7.2445	3.0331	9.3038	3.0	9.5	FCSSgr1
1453-11	1	2.3259	5.5328	2.9545	7.0281	3.0	7.0	FCSSgr1
1453-11	1	2.3332	5.4784	2.9637	6.9590	3.0	7.0	FCSSgr1
1453-11	1	2.3444	5.5030	2.9780	6.9903	3.0	7.0	FCSSgr1
1453-11	1	2.3489	5.5408	2.9837	7.0382	3.0	7.0	FCSSgr1
1453-11	1	2.3516	5.5730	2.9872	7.0791	3.0	7.0	FCSSgr1
1453-11	1	2.3572	5.5857	2.9942	7.0953	3.0	7.0	FCSSgr1
1453-11	1	2.3795	5.4929	3.0225	6.9774	3.0	7.0	FCSSgr1
1453-11	1	2.3830	5.5455	3.0270	7.0442	3.0	7.0	FCSSgr1
1453-11	1	2.3466	6.0189	2.9808	7.6455	3.0	7.5	FCSSgr1
1453-11	1	3.3377	5.7174	4.2397	7.2626	4.0	7.5	FCSSgr1
1453-11	1	2.3327	6.2474	2.9632	7.9358	3.0	8.0	FCSSgr1
1453-11	1	2.3331	6.2861	2.9637	7.9850	3.0	8.0	FCSSgr1
1453-11	1	2.3339	6.2527	2.9646	7.9425	3.0	8.0	FCSSgr1
1453-11	1	2.3362	6.2705	2.9675	7.9652	3.0	8.0	FCSSgr1
1453-11	1	2.3401	6.1244	2.9725	7.7795	3.0	8.0	FCSSgr1
1453-11	1	2.3407	6.2600	2.9733	7.9518	3.0	8.0	FCSSgr1
1453-11	1	2.3417	6.2894	2.9746	7.9892	3.0	8.0	FCSSgr1
1453-11	1	2.3420	6.2556	2.9749	7.9462	3.0	8.0	FCSSgr1
1453-11	1	2.3474	6.2708	2.9817	7.9656	3.0	8.0	FCSSgr1
1453-11	1	2.3494	6.3217	2.9843	8.0302	3.0	8.0	FCSSgr1
1453-11	1	2.3502	6.3193	2.9853	8.0271	3.0	8.0	FCSSgr1
1453-11	1	2.3503	6.3002	2.9855	8.0029	3.0	8.0	FCSSgr1
1453-11	1	2.3506	6.2636	2.9859	7.9564	3.0	8.0	FCSSgr1
1453-11	1	2.3516	6.2870	2.9872	7.9861	3.0	8.0	FCSSgr1

1453-11	1	2.3520	6.3317	2.9877	8.0429	3.0	8.0	FCSSgr1
1453-11	1	2.3537	6.3027	2.9899	8.0061	3.0	8.0	FCSSgr1
1453-11	1	2.3547	6.2968	2.9911	7.9986	3.0	8.0	FCSSgr1
1453-11	1	2.3585	6.2849	2.9959	7.9835	3.0	8.0	FCSSgr1
1453-11	1	2.3655	6.3015	3.0047	8.0045	3.0	8.0	FCSSgr1
1453-11	1	2.3670	6.2578	3.0067	7.9490	3.0	8.0	FCSSgr1
1453-11	1	2.3770	6.3437	3.0194	8.0582	3.0	8.0	FCSSgr1
1453-11	1	2.3784	6.3576	3.0212	8.0758	3.0	8.0	FCSSgr1
1453-11	1	2.3795	6.3073	3.0226	8.0118	3.0	8.0	FCSSgr1
1453-11	1	2.3822	6.3012	3.0260	8.0041	3.0	8.0	FCSSgr1
1453-11	1	2.3997	6.3208	3.0482	8.0290	3.0	8.0	FCSSgr1
1453-11	1	2.4032	6.3719	3.0526	8.0939	3.0	8.0	FCSSgr1
1453-11	1	2.4060	6.2360	3.0562	7.9213	3.0	8.0	FCSSgr1
1453-11	1	2.4404	6.4726	3.0999	8.2219	3.0	8.0	FCSSgr1
1453-11	1	3.1355	6.2440	3.9829	7.9315	4.0	8.0	FCSSgr1
1453-11	1	3.1639	6.2878	4.0190	7.9871	4.0	8.0	FCSSgr1
1453-11	1	3.1747	6.2523	4.0326	7.9420	4.0	8.0	FCSSgr1
1453-11	1	3.8603	6.1266	4.9036	7.7824	5.0	8.0	FCSSgr1
1453-11	1	2.3665	6.8205	3.0061	8.6638	3.0	8.5	FCSSgr1
1453-11	1	2.3655	7.2539	3.0048	9.2143	3.0	9.0	FCSSgr1
1453-11	1	2.3965	7.3515	3.0441	9.3382	3.0	9.5	FCSSgr1
1453-11	1	2.4148	7.3880	3.0674	9.3846	3.0	9.5	FCSSgr1
1472-11	1	2.2761	4.6415	2.9758	6.0685	3.0	6.0	FCSSgr1
1472-11	1	2.2854	5.4419	2.9880	7.1150	3.0	7.0	FCSSgr1
1472-11	1	2.2958	5.4188	3.0017	7.0848	3.0	7.0	FCSSgr1
1472-11	1	2.2963	5.4129	3.0023	7.0771	3.0	7.0	FCSSgr1
1472-11	1	3.0542	5.4301	3.9932	7.0996	4.0	7.0	FCSSgr1
1472-11	1	3.1077	5.3793	4.0632	7.0332	4.0	7.0	FCSSgr1
1472-11	1	3.1209	5.4413	4.0805	7.1142	4.0	7.0	FCSSgr1
1472-11	1	2.2686	6.1712	2.9661	8.0685	3.0	8.0	FCSSgr1
1472-11	1	2.2736	6.1429	2.9726	8.0315	3.0	8.0	FCSSgr1
1472-11	1	2.2786	6.1743	2.9792	8.0725	3.0	8.0	FCSSgr1
1472-11	1	2.2795	6.0904	2.9803	7.9628	3.0	8.0	FCSSgr1

1472-11	1	2.2803	6.2050	2.9813	8.1127	3.0	8.0	FCSSgr1
1472-11	1	2.2805	6.1761	2.9817	8.0749	3.0	8.0	FCSSgr1
1472-11	1	2.2819	6.1256	2.9834	8.0089	3.0	8.0	FCSSgr1
1472-11	1	2.2825	6.1749	2.9842	8.0733	3.0	8.0	FCSSgr1
1472-11	1	2.2831	6.1440	2.9850	8.0329	3.0	8.0	FCSSgr1
1472-11	1	2.2833	6.2068	2.9853	8.1150	3.0	8.0	FCSSgr1
1472-11	1	2.2856	6.2036	2.9883	8.1108	3.0	8.0	FCSSgr1
1472-11	1	2.2862	6.2240	2.9891	8.1375	3.0	8.0	FCSSgr1
1472-11	1	2.2869	6.1856	2.9900	8.0873	3.0	8.0	FCSSgr1
1472-11	1	2.2882	6.1738	2.9917	8.0719	3.0	8.0	FCSSgr1
1472-11	1	2.2883	6.1316	2.9918	8.0167	3.0	8.0	FCSSgr1
1472-11	1	2.2885	6.1911	2.9922	8.0946	3.0	8.0	FCSSgr1
1472-11	1	2.2887	6.2226	2.9924	8.1357	3.0	8.0	FCSSgr1
1472-11	1	2.2897	6.1952	2.9937	8.0999	3.0	8.0	FCSSgr1
1472-11	1	2.2905	6.1790	2.9947	8.0787	3.0	8.0	FCSSgr1
1472-11	1	2.2907	6.2253	2.9949	8.1393	3.0	8.0	FCSSgr1
1472-11	1	2.2908	6.1547	2.9951	8.0470	3.0	8.0	FCSSgr1
1472-11	1	2.2910	6.1624	2.9954	8.0571	3.0	8.0	FCSSgr1
1472-11	1	2.2914	6.1512	2.9958	8.0424	3.0	8.0	FCSSgr1
1472-11	1	2.2928	6.2035	2.9977	8.1107	3.0	8.0	FCSSgr1
1472-11	1	2.2937	6.1914	2.9989	8.0950	3.0	8.0	FCSSgr1
1472-11	1	2.2952	6.1766	3.0008	8.0756	3.0	8.0	FCSSgr1
1472-11	1	2.2956	6.1655	3.0013	8.0611	3.0	8.0	FCSSgr1
1472-11	1	2.2964	6.2307	3.0024	8.1463	3.0	8.0	FCSSgr1
1472-11	1	2.2967	6.1461	3.0028	8.0357	3.0	8.0	FCSSgr1
1472-11	1	2.2969	6.1730	3.0031	8.0708	3.0	8.0	FCSSgr1
1472-11	1	2.2970	6.1781	3.0032	8.0776	3.0	8.0	FCSSgr1
1472-11	1	2.2979	6.1811	3.0044	8.0815	3.0	8.0	FCSSgr1
1472-11	1	2.2981	6.0613	3.0047	7.9248	3.0	8.0	FCSSgr1
1472-11	1	2.2995	6.0705	3.0065	7.9369	3.0	8.0	FCSSgr1
1472-11	1	2.3001	6.1853	3.0073	8.0870	3.0	8.0	FCSSgr1
1472-11	1	2.3035	6.1087	3.0118	7.9868	3.0	8.0	FCSSgr1
1472-11	1	2.3038	6.2195	3.0121	8.1316	3.0	8.0	FCSSgr1

1472-11	1	2.3236	6.1945	3.0379	8.0990	3.0	8.0	FCSSgr1
1472-11	1	2.3255	6.2728	3.0404	8.2014	3.0	8.0	FCSSgr1
1472-11	1	2.3259	6.2023	3.0410	8.1091	3.0	8.0	FCSSgr1
1472-11	1	2.3336	6.2996	3.0511	8.2364	3.0	8.0	FCSSgr1
1472-11	1	2.3553	6.2314	3.0794	8.1472	3.0	8.0	FCSSgr1
1472-11	1	3.1017	6.2132	4.0553	8.1234	4.0	8.0	FCSSgr1
1472-11	1	2.2838	6.6738	2.9860	8.7256	3.0	8.5	FCSSgr1
1472-11	1	2.2922	6.8559	2.9969	8.9637	3.0	9.0	FCSSgr1
1472-11	1	2.3073	7.0604	3.0166	9.2311	3.0	9.0	FCSSgr1
1472-11	1	2.2777	7.7697	2.9779	10.1585	3.0	10.0	FCSSgr1
1472-11	1	2.2941	7.7139	2.9994	10.0855	3.0	10.0	FCSSgr1
1472-11	1	2.2953	8.4783	3.0010	11.0849	3.0	11.0	FCSSgr1
1472-11	1	2.3028	8.5016	3.0108	11.1153	3.0	11.0	FCSSgr1
1472-11	1	2.3225	8.5464	3.0365	11.1739	3.0	11.0	FCSSgr1
1472-11	1	2.2915	8.8798	2.9961	11.6099	3.0	11.5	FCSSgr1
1472-11	1	2.3938	not recorded	3.1298	NA	3.0	NA	FCSSgr1
1472-11	1	2.8880	not recorded	3.7760	NA	4.0	NA	FCSSgr1
1478-11	1	2.2924	5.4020	2.9732	7.0062	3.0	7.0	FCSSgr1
1478-11	1	2.3223	5.4936	3.0120	7.1250	3.0	7.0	FCSSgr1
1478-11	1	2.3354	5.5053	3.0289	7.1401	3.0	7.0	FCSSgr1
1478-11	1	2.2883	5.9557	2.9678	7.7244	3.0	7.5	FCSSgr1
1478-11	1	2.2474	6.0131	2.9148	7.7988	3.0	8.0	FCSSgr1
1478-11	1	2.2650	6.0154	2.9376	7.8018	3.0	8.0	FCSSgr1
1478-11	1	2.2681	6.0284	2.9416	7.8186	3.0	8.0	FCSSgr1
1478-11	1	2.2706	6.1205	2.9449	7.9380	3.0	8.0	FCSSgr1
1478-11	1	2.2734	6.1218	2.9485	7.9397	3.0	8.0	FCSSgr1
1478-11	1	2.2739	6.0896	2.9492	7.8980	3.0	8.0	FCSSgr1
1478-11	1	2.2813	6.1224	2.9588	7.9405	3.0	8.0	FCSSgr1
1478-11	1	2.2824	6.1847	2.9602	8.0213	3.0	8.0	FCSSgr1
1478-11	1	2.2852	6.2125	2.9638	8.0574	3.0	8.0	FCSSgr1
1478-11	1	2.2887	6.1905	2.9684	8.0288	3.0	8.0	FCSSgr1
1478-11	1	2.2928	6.2181	2.9736	8.0647	3.0	8.0	FCSSgr1
1478-11	1	2.2963	6.1775	2.9782	8.0120	3.0	8.0	FCSSgr1

1478-11	1	2.3004	6.2154	2.9835	8.0611	3.0	8.0	FCSSgr1
1478-11	1	2.3031	6.2424	2.9870	8.0962	3.0	8.0	FCSSgr1
1478-11	1	2.3048	6.2164	2.9892	8.0624	3.0	8.0	FCSSgr1
1478-11	1	2.3071	6.0687	2.9922	7.8709	3.0	8.0	FCSSgr1
1478-11	1	2.3078	6.1949	2.9931	8.0345	3.0	8.0	FCSSgr1
1478-11	1	2.3114	6.2202	2.9978	8.0674	3.0	8.0	FCSSgr1
1478-11	1	2.3254	6.2833	3.0159	8.1492	3.0	8.0	FCSSgr1
1478-11	1	2.3370	6.1905	3.0310	8.0288	3.0	8.0	FCSSgr1
1478-11	1	2.3437	6.3354	3.0397	8.2168	3.0	8.0	FCSSgr1
1478-11	1	2.3500	6.2456	3.0479	8.1003	3.0	8.0	FCSSgr1
1478-11	1	2.3566	6.2004	3.0564	8.0416	3.0	8.0	FCSSgr1
1478-11	1	2.3755	6.3633	3.0810	8.2529	3.0	8.5	FCSSgr1
1478-11	1	2.4036	6.4031	3.1174	8.3046	3.0	8.5	FCSSgr1
1478-11	1	2.4117	6.4511	3.1279	8.3669	3.0	8.5	FCSSgr1
1478-11	1	2.4147	6.4433	3.1318	8.3567	3.0	8.5	FCSSgr1
1478-11	1	2.4544	6.4905	3.1833	8.4179	3.0	8.5	FCSSgr1
1478-11	1	2.2774	7.4777	2.9537	9.6983	3.0	9.5	FCSSgr1
1478-11	1	2.2783	7.4717	2.9549	9.6905	3.0	9.5	FCSSgr1
1478-11	1	2.3012	7.5682	2.9845	9.8157	3.0	10.0	FCSSgr1
1478-11	1	2.3360	7.8565	3.0296	10.1895	3.0	10.0	FCSSgr1
1478-11	1	2.2789	8.5058	2.9556	11.0317	3.0	11.0	FCSSgr1
1478-11	1	2.2953	8.4357	2.9769	10.9408	3.0	11.0	FCSSgr1
1478-11	1	2.3111	8.5533	2.9974	11.0932	3.0	11.0	FCSSgr1
1478-11	1	2.3188	8.5528	3.0074	11.0927	3.0	11.0	FCSSgr1
1478-11	1	2.3370	8.6405	3.0309	11.2064	3.0	11.0	FCSSgr1
1478-11	1	2.2628	9.6965	2.9348	12.5760	3.0	12.5	FCSSgr1
1478-11	1	2.2958	9.8996	2.9775	12.8394	3.0	13.0	FCSSgr1
1478-11	1	4.6963	13.4491	6.0909	17.4429	6.0	17.5	FCSSgr1
1518-11	1	2.3311	5.4839	2.9747	6.9982	3.0	7.0	FCSSgr1
1518-11	1	2.3337	5.4846	2.9780	6.9991	3.0	7.0	FCSSgr1
1518-11	1	2.3447	5.4923	2.9921	7.0089	3.0	7.0	FCSSgr1
1518-11	1	2.3504	5.4887	2.9994	7.0043	3.0	7.0	FCSSgr1
1518-11	1	2.3553	5.5035	3.0057	7.0232	3.0	7.0	FCSSgr1

1518-11	1	2.3772	5.5673	3.0336	7.1046	3.0	7.0	FCSSgr1
1518-11	1	2.3790	5.5786	3.0359	7.1190	3.0	7.0	FCSSgr1
1518-11	1	3.1684	5.4991	4.0433	7.0176	4.0	7.0	FCSSgr1
1518-11	1	2.3697	5.7113	3.0240	7.2884	3.0	7.5	FCSSgr1
1518-11	1	2.3768	6.0030	3.0331	7.6606	3.0	7.5	FCSSgr1
1518-11	1	2.3211	6.2947	2.9620	8.0328	3.0	8.0	FCSSgr1
1518-11	1	2.3251	6.2462	2.9672	7.9710	3.0	8.0	FCSSgr1
1518-11	1	2.3313	6.2765	2.9751	8.0096	3.0	8.0	FCSSgr1
1518-11	1	2.3379	6.2733	2.9835	8.0055	3.0	8.0	FCSSgr1
1518-11	1	2.3387	6.2971	2.9845	8.0359	3.0	8.0	FCSSgr1
1518-11	1	2.3414	6.2603	2.9880	7.9889	3.0	8.0	FCSSgr1
1518-11	1	2.3423	6.2852	2.9891	8.0207	3.0	8.0	FCSSgr1
1518-11	1	2.3440	6.3086	2.9912	8.0506	3.0	8.0	FCSSgr1
1518-11	1	2.3443	6.3116	2.9916	8.0544	3.0	8.0	FCSSgr1
1518-11	1	2.3456	6.2268	2.9933	7.9461	3.0	8.0	FCSSgr1
1518-11	1	2.3506	6.3100	2.9997	8.0524	3.0	8.0	FCSSgr1
1518-11	1	2.3522	6.2649	3.0018	7.9948	3.0	8.0	FCSSgr1
1518-11	1	2.3773	6.3329	3.0338	8.0816	3.0	8.0	FCSSgr1
1518-11	1	2.3811	6.3468	3.0386	8.0994	3.0	8.0	FCSSgr1
1518-11	1	2.3886	6.3378	3.0481	8.0878	3.0	8.0	FCSSgr1
1518-11	1	2.3429	7.5050	2.9899	9.5773	3.0	9.5	FCSSgr1
1518-11	1	2.3478	7.8287	2.9961	9.9905	3.0	10.0	FCSSgr1
1518-11	1	2.3586	7.7571	3.0098	9.8991	3.0	10.0	FCSSgr1
1518-11	1	2.3354	8.5602	2.9803	10.9239	3.0	11.0	FCSSgr1
1521-11	1	2.3080	5.4322	2.9953	7.0499	3.0	7.0	FCSSgr2
1521-11	1	2.3277	5.4475	3.0209	7.0697	3.0	7.0	FCSSgr2
1521-11	1	2.2868	5.9498	2.9678	7.7217	3.0	7.5	FCSSgr2
1521-11	1	2.3039	6.1974	2.9899	8.0430	3.0	8.0	FCSSgr2
1521-11	1	2.3197	6.2359	3.0106	8.0930	3.0	8.0	FCSSgr2
1521-11	1	2.3251	6.2452	3.0176	8.1050	3.0	8.0	FCSSgr2
1521-11	1	2.3327	6.2692	3.0274	8.1361	3.0	8.0	FCSSgr2
1521-11	1	2.3365	6.1948	3.0323	8.0396	3.0	8.0	FCSSgr2
1521-11	1	3.1901	6.1965	4.1402	8.0418	4.0	8.0	FCSSgr2

1521-11	1	2.2558	7.5767	2.9276	9.8330	3.0	10.0	FCSSgr2
1521-11	1	2.2889	7.5574	2.9706	9.8080	3.0	10.0	FCSSgr2
1521-11	1	2.2980	7.5701	2.9823	9.8244	3.0	10.0	FCSSgr2
1521-11	1	2.3015	7.5975	2.9869	9.8600	3.0	10.0	FCSSgr2
1521-11	1	2.3038	7.7290	2.9899	10.0307	3.0	10.0	FCSSgr2
1521-11	1	2.3073	7.7809	2.9944	10.0980	3.0	10.0	FCSSgr2
1521-11	1	2.3142	7.7133	3.0034	10.0103	3.0	10.0	FCSSgr2
1521-11	1	2.3176	7.6647	3.0078	9.9473	3.0	10.0	FCSSgr2
1521-11	1	3.9241	7.7016	5.0928	9.9951	5.0	10.0	FCSSgr2
1521-11	1	2.3695	10.8092	3.0751	14.0281	3.0	14.0	FCSSgr2
1521-11	1	2.2326	not recorded	2.8975	NA	3.0	NA	FCSSgr2
1525-11	1	2.3278	5.4994	2.9620	6.9980	3.0	7.0	FCSSgr1
1525-11	1	2.3326	5.4837	2.9683	6.9779	3.0	7.0	FCSSgr1
1525-11	1	2.3940	5.5497	3.0463	7.0620	3.0	7.0	FCSSgr1
1525-11	1	3.2791	5.7485	4.1726	7.3148	4.0	7.5	FCSSgr1
1525-11	1	2.3104	6.2755	2.9399	7.9854	3.0	8.0	FCSSgr1
1525-11	1	2.3294	6.3012	2.9642	8.0182	3.0	8.0	FCSSgr1
1525-11	1	2.3301	6.2820	2.9650	7.9937	3.0	8.0	FCSSgr1
1525-11	1	2.3305	6.2756	2.9656	7.9856	3.0	8.0	FCSSgr1
1525-11	1	2.3329	6.3276	2.9686	8.0518	3.0	8.0	FCSSgr1
1525-11	1	2.3348	6.2653	2.9710	7.9725	3.0	8.0	FCSSgr1
1525-11	1	2.3351	6.2647	2.9714	7.9718	3.0	8.0	FCSSgr1
1525-11	1	2.3355	6.2806	2.9719	7.9920	3.0	8.0	FCSSgr1
1525-11	1	2.3360	6.2585	2.9725	7.9639	3.0	8.0	FCSSgr1
1525-11	1	2.3364	6.2719	2.9730	7.9808	3.0	8.0	FCSSgr1
1525-11	1	2.3397	6.2592	2.9772	7.9648	3.0	8.0	FCSSgr1
1525-11	1	2.3405	6.2841	2.9783	7.9964	3.0	8.0	FCSSgr1
1525-11	1	2.3418	6.2738	2.9799	7.9833	3.0	8.0	FCSSgr1
1525-11	1	2.3450	6.2638	2.9839	7.9706	3.0	8.0	FCSSgr1
1525-11	1	2.3472	6.3263	2.9868	8.0501	3.0	8.0	FCSSgr1
1525-11	1	2.3569	6.3160	2.9991	8.0370	3.0	8.0	FCSSgr1
1525-11	1	2.3581	6.3102	3.0007	8.0296	3.0	8.0	FCSSgr1
1525-11	1	2.3638	6.2476	3.0079	7.9500	3.0	8.0	FCSSgr1

1525-11	1	2.3715	6.2740	3.0178	7.9836	3.0	8.0	FCSSgr1
1525-11	1	2.3754	6.2752	3.0227	7.9851	3.0	8.0	FCSSgr1
1525-11	1	2.4196	6.3142	3.0790	8.0347	3.0	8.0	FCSSgr1
1525-11	1	2.4493	6.4618	3.1168	8.2226	3.0	8.0	FCSSgr1
1525-11	1	2.4880	7.6283	3.1659	9.7069	3.0	9.5	FCSSgr1
1525-11	1	2.3400	7.7705	2.9776	9.8879	3.0	10.0	FCSSgr1
1525-11	1	2.3647	7.6835	3.0090	9.7772	3.0	10.0	FCSSgr1
1525-11	1	2.3860	7.7102	3.0361	9.8112	3.0	10.0	FCSSgr1
1525-11	1	2.3747	8.6949	3.0218	11.0641	3.0	11.0	FCSSgr1
1527-11	1	2.1185	4.2675	2.7549	5.5493	3.0	5.5	FCSSgr1
1527-11	1	2.3066	4.1810	2.9995	5.4369	3.0	5.5	FCSSgr1
1527-11	1	2.2963	4.8679	2.9860	6.3302	3.0	6.5	FCSSgr1
1527-11	1	2.2981	5.3818	2.9884	6.9984	3.0	7.0	FCSSgr1
1527-11	1	2.3048	5.4170	2.9971	7.0442	3.0	7.0	FCSSgr1
1527-11	1	2.3085	5.3646	3.0020	6.9761	3.0	7.0	FCSSgr1
1527-11	1	2.3122	5.4998	3.0068	7.1518	3.0	7.0	FCSSgr1
1527-11	1	2.3122	5.4197	3.0068	7.0476	3.0	7.0	FCSSgr1
1527-11	1	2.3146	5.4678	3.0099	7.1103	3.0	7.0	FCSSgr1
1527-11	1	2.3197	5.4950	3.0164	7.1456	3.0	7.0	FCSSgr1
1527-11	1	3.1051	5.3858	4.0378	7.0036	4.0	7.0	FCSSgr1
1527-11	1	2.3006	5.7680	2.9916	7.5006	3.0	7.5	FCSSgr1
1527-11	1	2.3049	5.6118	2.9972	7.2974	3.0	7.5	FCSSgr1
1527-11	1	2.3150	5.8283	3.0103	7.5790	3.0	7.5	FCSSgr1
1527-11	1	2.2724	6.1484	2.9550	7.9953	3.0	8.0	FCSSgr1
1527-11	1	2.2804	6.1906	2.9654	8.0502	3.0	8.0	FCSSgr1
1527-11	1	2.2812	6.1360	2.9665	7.9791	3.0	8.0	FCSSgr1
1527-11	1	2.2917	6.0091	2.9800	7.8141	3.0	8.0	FCSSgr1
1527-11	1	2.2935	6.2057	2.9825	8.0698	3.0	8.0	FCSSgr1
1527-11	1	2.2942	6.2074	2.9834	8.0719	3.0	8.0	FCSSgr1
1527-11	1	2.2953	6.1888	2.9847	8.0477	3.0	8.0	FCSSgr1
1527-11	1	2.2956	6.1052	2.9851	7.9391	3.0	8.0	FCSSgr1
1527-11	1	2.2973	6.1914	2.9874	8.0512	3.0	8.0	FCSSgr1
1527-11	1	2.2979	6.1943	2.9882	8.0550	3.0	8.0	FCSSgr1

1527-11	1	2.2997	6.2124	2.9904	8.0785	3.0	8.0	FCSSgr1
1527-11	1	2.3001	6.1451	2.9910	7.9909	3.0	8.0	FCSSgr1
1527-11	1	2.3001	6.1808	2.9910	8.0374	3.0	8.0	FCSSgr1
1527-11	1	2.3004	6.1644	2.9914	8.0161	3.0	8.0	FCSSgr1
1527-11	1	2.3015	6.2064	2.9929	8.0706	3.0	8.0	FCSSgr1
1527-11	1	2.3017	6.2011	2.9931	8.0638	3.0	8.0	FCSSgr1
1527-11	1	2.3018	6.2186	2.9932	8.0866	3.0	8.0	FCSSgr1
1527-11	1	2.3020	6.1420	2.9935	7.9870	3.0	8.0	FCSSgr1
1527-11	1	2.3020	6.2176	2.9935	8.0852	3.0	8.0	FCSSgr1
1527-11	1	2.3029	6.1944	2.9946	8.0550	3.0	8.0	FCSSgr1
1527-11	1	2.3034	6.1706	2.9953	8.0241	3.0	8.0	FCSSgr1
1527-11	1	2.3040	6.1985	2.9961	8.0604	3.0	8.0	FCSSgr1
1527-11	1	2.3042	6.2201	2.9963	8.0885	3.0	8.0	FCSSgr1
1527-11	1	2.3060	6.1342	2.9986	7.9768	3.0	8.0	FCSSgr1
1527-11	1	2.3065	6.1677	2.9994	8.0203	3.0	8.0	FCSSgr1
1527-11	1	2.3072	6.1699	3.0002	8.0233	3.0	8.0	FCSSgr1
1527-11	1	2.3078	6.2100	3.0010	8.0753	3.0	8.0	FCSSgr1
1527-11	1	2.3078	6.2221	3.0011	8.0911	3.0	8.0	FCSSgr1
1527-11	1	2.3079	6.2329	3.0012	8.1051	3.0	8.0	FCSSgr1
1527-11	1	2.3086	6.2650	3.0021	8.1469	3.0	8.0	FCSSgr1
1527-11	1	2.3090	6.2449	3.0026	8.1207	3.0	8.0	FCSSgr1
1527-11	1	2.3096	6.1761	3.0034	8.0312	3.0	8.0	FCSSgr1
1527-11	1	2.3098	6.1585	3.0036	8.0083	3.0	8.0	FCSSgr1
1527-11	1	2.3103	6.2350	3.0043	8.1078	3.0	8.0	FCSSgr1
1527-11	1	2.3124	6.2051	3.0070	8.0689	3.0	8.0	FCSSgr1
1527-11	1	2.3133	6.2243	3.0082	8.0940	3.0	8.0	FCSSgr1
1527-11	1	2.3134	6.2098	3.0082	8.0751	3.0	8.0	FCSSgr1
1527-11	1	2.3160	6.2217	3.0117	8.0906	3.0	8.0	FCSSgr1
1527-11	1	2.3166	6.2393	3.0124	8.1134	3.0	8.0	FCSSgr1
1527-11	1	2.3179	6.2779	3.0142	8.1636	3.0	8.0	FCSSgr1
1527-11	1	2.3210	6.2617	3.0182	8.1426	3.0	8.0	FCSSgr1
1527-11	1	2.3225	6.2671	3.0202	8.1496	3.0	8.0	FCSSgr1
1527-11	1	2.3245	6.2983	3.0227	8.1902	3.0	8.0	FCSSgr1

1527-11	1	2.3249	6.1982	3.0233	8.0600	3.0	8.0	FCSSgr1
1527-11	1	2.3258	6.1970	3.0244	8.0584	3.0	8.0	FCSSgr1
1527-11	1	2.3258	6.1656	3.0244	8.0175	3.0	8.0	FCSSgr1
1527-11	1	2.3287	5.9876	3.0282	7.7862	3.0	8.0	FCSSgr1
1527-11	1	2.3295	6.3082	3.0292	8.2030	3.0	8.0	FCSSgr1
1527-11	1	2.3295	6.2434	3.0293	8.1188	3.0	8.0	FCSSgr1
1527-11	1	2.3343	6.1020	3.0355	7.9350	3.0	8.0	FCSSgr1
1527-11	1	2.3386	6.2779	3.0410	8.1637	3.0	8.0	FCSSgr1
1527-11	1	2.3404	6.2700	3.0434	8.1533	3.0	8.0	FCSSgr1
1527-11	1	3.0784	6.2000	4.0031	8.0623	4.0	8.0	FCSSgr1
1527-11	1	3.1084	6.2656	4.0421	8.1476	4.0	8.0	FCSSgr1
1527-11	1	3.1329	6.2655	4.0739	8.1476	4.0	8.0	FCSSgr1
1527-11	1	3.1494	6.2438	4.0954	8.1192	4.0	8.0	FCSSgr1
1527-11	1	2.2949	6.3576	2.9843	8.2673	3.0	8.5	FCSSgr1
1527-11	1	2.3730	6.4350	3.0858	8.3680	3.0	8.5	FCSSgr1
1527-11	1	2.3079	6.8437	3.0011	8.8993	3.0	9.0	FCSSgr1
1527-11	1	2.3022	7.6799	2.9937	9.9868	3.0	10.0	FCSSgr1
1527-11	1	2.3186	7.7236	3.0151	10.0436	3.0	10.0	FCSSgr1
1527-11	1	2.3370	7.7374	3.0390	10.0616	3.0	10.0	FCSSgr1
1527-11	1	2.3101	7.9064	3.0040	10.2814	3.0	10.5	FCSSgr1
1527-11	1	2.3539	7.9619	3.0610	10.3534	3.0	10.5	FCSSgr1
1527-11	1	2.2842	8.4967	2.9703	11.0489	3.0	11.0	FCSSgr1
1527-11	1	2.2936	8.4024	2.9826	10.9263	3.0	11.0	FCSSgr1
1527-11	1	2.2953	8.4676	2.9848	11.0111	3.0	11.0	FCSSgr1
1527-11	1	2.2989	8.4938	2.9895	11.0452	3.0	11.0	FCSSgr1
1527-11	1	2.2996	8.4229	2.9903	10.9529	3.0	11.0	FCSSgr1
1527-11	1	2.3030	8.4392	2.9948	10.9742	3.0	11.0	FCSSgr1
1527-11	1	2.3132	8.5543	3.0081	11.1238	3.0	11.0	FCSSgr1
1527-11	1	2.3192	8.5307	3.0158	11.0931	3.0	11.0	FCSSgr1
1527-11	1	2.3101	9.9281	3.0039	12.9102	3.0	13.0	FCSSgr1
1527-11	1	4.6710	10.6059	6.0741	13.7917	6.0	14.0	FCSSgr1
1531-11	1	2.3043	5.4048	2.9616	6.9464	3.0	7.0	FCSSgr1
1531-11	1	2.3087	5.3876	2.9672	6.9244	3.0	7.0	FCSSgr1

1531-11	1	2.3121	5.4479	2.9716	7.0018	3.0	7.0	FCSSgr1
1531-11	1	2.3301	5.4569	2.9947	7.0134	3.0	7.0	FCSSgr1
1531-11	1	2.4136	5.7973	3.1021	7.4508	3.0	7.5	FCSSgr1
1531-11	1	2.2955	6.1616	2.9502	7.9191	3.0	8.0	FCSSgr1
1531-11	1	2.3005	6.1233	2.9566	7.8699	3.0	8.0	FCSSgr1
1531-11	1	2.3028	6.2095	2.9597	7.9807	3.0	8.0	FCSSgr1
1531-11	1	2.3058	6.2921	2.9635	8.0868	3.0	8.0	FCSSgr1
1531-11	1	2.3161	6.1985	2.9767	7.9665	3.0	8.0	FCSSgr1
1531-11	1	2.3170	6.3808	2.9778	8.2008	3.0	8.0	FCSSgr1
1531-11	1	2.3194	6.1644	2.9809	7.9227	3.0	8.0	FCSSgr1
1531-11	1	2.3198	6.2238	2.9814	7.9990	3.0	8.0	FCSSgr1
1531-11	1	2.3205	6.3034	2.9824	8.1013	3.0	8.0	FCSSgr1
1531-11	1	2.3208	6.0595	2.9828	7.7878	3.0	8.0	FCSSgr1
1531-11	1	2.3210	6.2723	2.9830	8.0613	3.0	8.0	FCSSgr1
1531-11	1	2.3212	6.2720	2.9832	8.0610	3.0	8.0	FCSSgr1
1531-11	1	2.3222	6.2658	2.9846	8.0530	3.0	8.0	FCSSgr1
1531-11	1	2.3233	6.2472	2.9860	8.0291	3.0	8.0	FCSSgr1
1531-11	1	2.3302	6.2690	2.9948	8.0571	3.0	8.0	FCSSgr1
1531-11	1	2.3369	6.2451	3.0034	8.0264	3.0	8.0	FCSSgr1
1531-11	1	2.3533	6.3351	3.0245	8.1421	3.0	8.0	FCSSgr1
1531-11	1	2.3907	6.3885	3.0726	8.2107	3.0	8.0	FCSSgr1
1531-11	1	2.4381	6.4810	3.1335	8.3296	3.0	8.5	FCSSgr1
1531-11	1	2.5900	6.9086	3.3288	8.8792	3.5	9.0	FCSSgr1
1531-11	1	2.3220	7.4033	2.9843	9.5150	3.0	9.5	FCSSgr1
1531-11	1	2.3341	7.4199	2.9998	9.5363	3.0	9.5	FCSSgr1
1531-11	1	2.3071	7.8372	2.9652	10.0727	3.0	10.0	FCSSgr1
1531-11	1	2.3089	7.9285	2.9675	10.1900	3.0	10.0	FCSSgr1
1531-11	1	2.3184	7.8207	2.9797	10.0514	3.0	10.0	FCSSgr1
1531-11	1	2.3199	7.5917	2.9817	9.7571	3.0	10.0	FCSSgr1
1531-11	1	2.3220	7.7546	2.9843	9.9664	3.0	10.0	FCSSgr1
1531-11	1	2.3492	7.7819	3.0193	10.0015	3.0	10.0	FCSSgr1
1531-11	1	2.5237	8.4018	3.2436	10.7983	3.0	11.0	FCSSgr1
1537-11	1	2.2770	5.4080	2.9642	7.0403	3.0	7.0	FCSSgr1

1537-11	1	2.2848	5.3925	2.9744	7.0201	3.0	7.0	FCSSgr1
1537-11	1	2.2865	5.4407	2.9766	7.0827	3.0	7.0	FCSSgr1
1537-11	1	2.2869	5.4132	2.9771	7.0470	3.0	7.0	FCSSgr1
1537-11	1	2.2881	5.4128	2.9786	7.0465	3.0	7.0	FCSSgr1
1537-11	1	2.2892	5.4193	2.9801	7.0549	3.0	7.0	FCSSgr1
1537-11	1	2.2961	5.3184	2.9891	6.9236	3.0	7.0	FCSSgr1
1537-11	1	2.2988	5.4397	2.9927	7.0815	3.0	7.0	FCSSgr1
1537-11	1	2.3015	5.4325	2.9961	7.0721	3.0	7.0	FCSSgr1
1537-11	1	2.3046	5.4497	3.0001	7.0945	3.0	7.0	FCSSgr1
1537-11	1	2.3053	5.4286	3.0010	7.0671	3.0	7.0	FCSSgr1
1537-11	1	2.3176	5.4863	3.0171	7.1421	3.0	7.0	FCSSgr1
1537-11	1	2.2743	6.1755	2.9607	8.0393	3.0	8.0	FCSSgr1
1537-11	1	2.2795	6.1865	2.9675	8.0537	3.0	8.0	FCSSgr1
1537-11	1	2.2862	6.1624	2.9762	8.0223	3.0	8.0	FCSSgr1
1537-11	1	2.2885	6.1566	2.9792	8.0148	3.0	8.0	FCSSgr1
1537-11	1	2.2886	6.1741	2.9794	8.0375	3.0	8.0	FCSSgr1
1537-11	1	2.2899	6.1638	2.9810	8.0241	3.0	8.0	FCSSgr1
1537-11	1	2.2916	6.1495	2.9832	8.0055	3.0	8.0	FCSSgr1
1537-11	1	2.2924	6.1892	2.9842	8.0572	3.0	8.0	FCSSgr1
1537-11	1	2.2928	6.1885	2.9848	8.0562	3.0	8.0	FCSSgr1
1537-11	1	2.2939	6.0397	2.9863	7.8625	3.0	8.0	FCSSgr1
1537-11	1	2.2941	6.1996	2.9865	8.0707	3.0	8.0	FCSSgr1
1537-11	1	2.2959	6.1804	2.9888	8.0458	3.0	8.0	FCSSgr1
1537-11	1	2.2980	6.2258	2.9916	8.1048	3.0	8.0	FCSSgr1
1537-11	1	2.3004	6.1771	2.9946	8.0414	3.0	8.0	FCSSgr1
1537-11	1	2.3051	6.2229	3.0008	8.1010	3.0	8.0	FCSSgr1
1537-11	1	2.3060	6.1697	3.0020	8.0318	3.0	8.0	FCSSgr1
1537-11	1	2.3111	6.2884	3.0086	8.1864	3.0	8.0	FCSSgr1
1537-11	1	2.3111	6.1527	3.0087	8.0097	3.0	8.0	FCSSgr1
1537-11	1	2.3122	6.1826	3.0100	8.0486	3.0	8.0	FCSSgr1
1537-11	1	2.3126	6.2225	3.0105	8.1005	3.0	8.0	FCSSgr1
1537-11	1	2.3155	6.2130	3.0144	8.0881	3.0	8.0	FCSSgr1
1537-11	1	2.3156	6.2019	3.0144	8.0737	3.0	8.0	FCSSgr1

1537-11	1	2.3156	6.2357	3.0145	8.1177	3.0	8.0	FCSSgr1
1537-11	1	2.3165	6.2219	3.0157	8.0997	3.0	8.0	FCSSgr1
1537-11	1	2.3176	6.1715	3.0170	8.0341	3.0	8.0	FCSSgr1
1537-11	1	2.3185	6.1869	3.0183	8.0541	3.0	8.0	FCSSgr1
1537-11	1	2.3191	6.2203	3.0190	8.0977	3.0	8.0	FCSSgr1
1537-11	1	2.3205	6.1641	3.0208	8.0245	3.0	8.0	FCSSgr1
1537-11	1	2.3220	6.2820	3.0229	8.1780	3.0	8.0	FCSSgr1
1537-11	1	2.3266	6.2111	3.0288	8.0857	3.0	8.0	FCSSgr1
1537-11	1	2.3292	6.2776	3.0322	8.1723	3.0	8.0	FCSSgr1
1537-11	1	2.3297	6.1622	3.0328	8.0220	3.0	8.0	FCSSgr1
1537-11	1	2.3319	6.1995	3.0357	8.0706	3.0	8.0	FCSSgr1
1537-11	1	2.3354	6.2455	3.0402	8.1305	3.0	8.0	FCSSgr1
1537-11	1	2.3056	7.6452	3.0015	9.9526	3.0	10.0	FCSSgr1
1537-11	1	2.3058	7.7480	3.0018	10.0865	3.0	10.0	FCSSgr1
1537-11	1	2.2735	8.3625	2.9596	10.8864	3.0	11.0	FCSSgr1
1537-11	1	2.3255	8.6124	3.0273	11.2117	3.0	11.0	FCSSgr1
1537-11	1	2.3464	8.6351	3.0546	11.2413	3.0	11.0	FCSSgr1
1537-11	1	2.3019	10.0219	2.9967	13.0466	3.0	13.0	FCSSgr1
1537-11	1	4.5109	not recorded	5.8723	NA	6.0	NA	FCSSgr1
1539-11	1	2.3388	5.5247	2.9651	7.0040	3.0	7.0	FCSSgr1
1539-11	1	2.3401	5.5193	2.9667	6.9972	3.0	7.0	FCSSgr1
1539-11	1	2.3670	5.5275	3.0008	7.0075	3.0	7.0	FCSSgr1
1539-11	1	2.4495	5.6950	3.1054	7.2199	3.0	7.0	FCSSgr1
1539-11	1	3.1493	5.5532	3.9925	7.0401	4.0	7.0	FCSSgr1
1539-11	1	3.1493	5.5211	3.9925	6.9995	4.0	7.0	FCSSgr1
1539-11	1	3.1721	5.5453	4.0215	7.0301	4.0	7.0	FCSSgr1
1539-11	1	2.4476	5.7636	3.1030	7.3068	3.0	7.5	FCSSgr1
1539-11	1	2.4868	5.8256	3.1526	7.3855	3.0	7.5	FCSSgr1
1539-11	1	3.9699	6.1083	5.0328	7.7438	5.0	7.5	FCSSgr1
1539-11	1	2.3267	6.3130	2.9497	8.0034	3.0	8.0	FCSSgr1
1539-11	1	2.3317	6.2699	2.9560	7.9488	3.0	8.0	FCSSgr1
1539-11	1	2.3319	6.2309	2.9563	7.8993	3.0	8.0	FCSSgr1
1539-11	1	2.3336	6.2996	2.9584	7.9863	3.0	8.0	FCSSgr1

1539-11	1	2.3346	6.2932	2.9598	7.9782	3.0	8.0	FCSSgr1
1539-11	1	2.3348	6.2651	2.9599	7.9426	3.0	8.0	FCSSgr1
1539-11	1	2.3367	6.3058	2.9623	7.9942	3.0	8.0	FCSSgr1
1539-11	1	2.3385	6.2787	2.9647	7.9599	3.0	8.0	FCSSgr1
1539-11	1	2.3387	6.2925	2.9649	7.9773	3.0	8.0	FCSSgr1
1539-11	1	2.3395	6.2747	2.9660	7.9548	3.0	8.0	FCSSgr1
1539-11	1	2.3403	6.3354	2.9669	8.0317	3.0	8.0	FCSSgr1
1539-11	1	2.3404	6.3307	2.9671	8.0258	3.0	8.0	FCSSgr1
1539-11	1	2.3407	6.2558	2.9674	7.9308	3.0	8.0	FCSSgr1
1539-11	1	2.3407	6.2904	2.9674	7.9747	3.0	8.0	FCSSgr1
1539-11	1	2.3422	6.2926	2.9694	7.9775	3.0	8.0	FCSSgr1
1539-11	1	2.3427	6.3267	2.9699	8.0207	3.0	8.0	FCSSgr1
1539-11	1	2.3428	6.3346	2.9701	8.0308	3.0	8.0	FCSSgr1
1539-11	1	2.3437	6.3292	2.9712	8.0239	3.0	8.0	FCSSgr1
1539-11	1	2.3453	6.2983	2.9733	7.9847	3.0	8.0	FCSSgr1
1539-11	1	2.3467	6.3796	2.9750	8.0878	3.0	8.0	FCSSgr1
1539-11	1	2.3472	6.2987	2.9756	7.9852	3.0	8.0	FCSSgr1
1539-11	1	2.3482	6.3039	2.9769	7.9919	3.0	8.0	FCSSgr1
1539-11	1	2.3486	6.3592	2.9775	8.0620	3.0	8.0	FCSSgr1
1539-11	1	2.3490	6.2882	2.9780	7.9720	3.0	8.0	FCSSgr1
1539-11	1	2.3497	6.3150	2.9788	8.0059	3.0	8.0	FCSSgr1
1539-11	1	2.3506	6.1857	2.9800	7.8420	3.0	8.0	FCSSgr1
1539-11	1	2.3508	6.2975	2.9802	7.9837	3.0	8.0	FCSSgr1
1539-11	1	2.3535	6.3431	2.9836	8.0415	3.0	8.0	FCSSgr1
1539-11	1	2.3540	6.2817	2.9843	7.9637	3.0	8.0	FCSSgr1
1539-11	1	2.3540	6.3014	2.9843	7.9887	3.0	8.0	FCSSgr1
1539-11	1	2.3543	6.2906	2.9847	7.9750	3.0	8.0	FCSSgr1
1539-11	1	2.3547	6.4352	2.9851	8.1583	3.0	8.0	FCSSgr1
1539-11	1	2.3585	6.3296	2.9900	8.0244	3.0	8.0	FCSSgr1
1539-11	1	2.3666	6.2407	3.0003	7.9117	3.0	8.0	FCSSgr1
1539-11	1	2.3682	6.3817	3.0024	8.0905	3.0	8.0	FCSSgr1
1539-11	1	2.3696	6.3436	3.0040	8.0422	3.0	8.0	FCSSgr1
1539-11	1	2.3721	6.3183	3.0072	8.0101	3.0	8.0	FCSSgr1

1539-11	1	2.3750	6.3945	3.0110	8.1066	3.0	8.0	FCSSgr1
1539-11	1	2.3806	6.4728	3.0180	8.2060	3.0	8.0	FCSSgr1
1539-11	1	2.3814	6.3721	3.0190	8.0783	3.0	8.0	FCSSgr1
1539-11	1	2.3896	6.4470	3.0294	8.1732	3.0	8.0	FCSSgr1
1539-11	1	2.3919	6.2597	3.0323	7.9358	3.0	8.0	FCSSgr1
1539-11	1	2.4381	6.4649	3.0910	8.1960	3.0	8.0	FCSSgr1
1539-11	1	3.1525	6.2519	3.9966	7.9259	4.0	8.0	FCSSgr1
1539-11	1	3.2001	6.3807	4.0569	8.0892	4.0	8.0	FCSSgr1
1539-11	1	2.3987	6.5366	3.0410	8.2869	3.0	8.5	FCSSgr1
1539-11	1	2.4025	6.7062	3.0458	8.5019	3.0	8.5	FCSSgr1
1539-11	1	2.4152	6.5197	3.0619	8.2654	3.0	8.5	FCSSgr1
1539-11	1	2.4570	6.5747	3.1149	8.3352	3.0	8.5	FCSSgr1
1539-11	1	2.5404	6.5600	3.2207	8.3164	3.0	8.5	FCSSgr1
1539-11	1	2.3672	7.5295	3.0010	9.5456	3.0	9.5	FCSSgr1
1539-11	1	2.3415	7.8209	2.9684	9.9150	3.0	10.0	FCSSgr1
1539-11	1	2.3530	7.9522	2.9831	10.0815	3.0	10.0	FCSSgr1
1539-11	1	2.3932	not recorded	3.0339	NA	3.0	NA	FCSSgr1
Hurka1	1	2.3469	6.0245	3.0069	7.7187	3.0	7.5	FCSSgr2
Hurka1	1	2.3496	5.7328	3.0103	7.3450	3.0	7.5	FCSSgr2
Hurka1	1	2.3496	5.8092	3.0104	7.4428	3.0	7.5	FCSSgr2
Hurka1	1	2.3590	5.9481	3.0224	7.6208	3.0	7.5	FCSSgr2
Hurka1	1	2.3652	5.9375	3.0304	7.6072	3.0	7.5	FCSSgr2
Hurka1	1	2.4306	5.9391	3.1141	7.6093	3.0	7.5	FCSSgr2
Hurka1	1	3.4149	5.7825	4.3752	7.4085	4.5	7.5	FCSSgr2
Hurka1	1	2.2819	6.0918	2.9236	7.8048	3.0	8.0	FCSSgr2
Hurka1	1	2.2991	6.3298	2.9456	8.1098	3.0	8.0	FCSSgr2
Hurka1	1	2.3076	6.0841	2.9566	7.7950	3.0	8.0	FCSSgr2
Hurka1	1	2.3280	6.1255	2.9826	7.8481	3.0	8.0	FCSSgr2
Hurka1	1	2.3284	6.2013	2.9831	7.9452	3.0	8.0	FCSSgr2
Hurka1	1	2.3340	6.2107	2.9904	7.9573	3.0	8.0	FCSSgr2
Hurka1	1	2.3366	6.1979	2.9937	7.9408	3.0	8.0	FCSSgr2
Hurka1	1	2.3388	6.2914	2.9965	8.0606	3.0	8.0	FCSSgr2
Hurka1	1	2.3388	6.2871	2.9965	8.0551	3.0	8.0	FCSSgr2

Hurka1	1	2.3409	6.3068	2.9992	8.0803	3.0	8.0	FCSSgr2
Hurka1	1	2.3474	6.2140	3.0076	7.9614	3.0	8.0	FCSSgr2
Hurka1	1	2.3596	6.2907	3.0232	8.0597	3.0	8.0	FCSSgr2
Hurka1	1	2.3607	6.3236	3.0246	8.1019	3.0	8.0	FCSSgr2
Hurka1	1	3.8327	6.1435	4.9104	7.8712	5.0	8.0	FCSSgr2
Hurka1	1	3.9512	6.2362	5.0623	7.9899	5.0	8.0	FCSSgr2
Hurka1	1	2.3062	6.7198	2.9547	8.6095	3.0	8.5	FCSSgr2
Hurka1	1	2.3164	6.6207	2.9678	8.4825	3.0	8.5	FCSSgr2
Hurka1	1	2.3403	6.4919	2.9984	8.3174	3.0	8.5	FCSSgr2
Hurka1	1	2.3562	6.7950	3.0188	8.7058	3.0	8.5	FCSSgr2
Hurka1	1	2.3938	6.4456	3.0669	8.2582	3.0	8.5	FCSSgr2
Hurka1	1	2.2932	7.1672	2.9380	9.1827	3.0	9.0	FCSSgr2
Hurka1	1	2.2998	7.0245	2.9466	8.9999	3.0	9.0	FCSSgr2
Hurka1	1	2.3028	7.0380	2.9504	9.0172	3.0	9.0	FCSSgr2
Hurka1	1	2.3107	7.0029	2.9604	8.9722	3.0	9.0	FCSSgr2
Hurka1	1	2.3231	7.0967	2.9763	9.0923	3.0	9.0	FCSSgr2
Hurka1	1	2.3286	7.1820	2.9834	9.2016	3.0	9.0	FCSSgr2
Hurka1	1	2.3397	7.0824	2.9976	9.0740	3.0	9.0	FCSSgr2
Hurka1	1	2.3403	6.9225	2.9984	8.8692	3.0	9.0	FCSSgr2
Hurka1	1	2.3546	7.1950	3.0167	9.2183	3.0	9.0	FCSSgr2
Hurka1	1	2.3681	7.2140	3.0340	9.2426	3.0	9.0	FCSSgr2
Hurka1	1	2.4144	7.0192	3.0933	8.9931	3.0	9.0	FCSSgr2
Hurka1	1	2.3066	7.2683	2.9552	9.3122	3.0	9.5	FCSSgr2
Hurka1	1	2.3086	7.5615	2.9578	9.6879	3.0	9.5	FCSSgr2
Hurka1	1	2.3161	7.5960	2.9674	9.7320	3.0	9.5	FCSSgr2
Hurka1	1	2.3178	7.2293	2.9695	9.2622	3.0	9.5	FCSSgr2
Hurka1	1	2.3363	7.2664	2.9933	9.3098	3.0	9.5	FCSSgr2
Hurka1	1	2.3385	7.3164	2.9961	9.3739	3.0	9.5	FCSSgr2
Hurka1	1	2.3389	7.3937	2.9966	9.4729	3.0	9.5	FCSSgr2
Hurka1	1	2.3412	7.4307	2.9996	9.5203	3.0	9.5	FCSSgr2
Hurka1	1	2.3424	7.4935	3.0011	9.6007	3.0	9.5	FCSSgr2
Hurka1	1	2.3435	7.2697	3.0025	9.3140	3.0	9.5	FCSSgr2
Hurka1	1	2.3608	7.2712	3.0247	9.3159	3.0	9.5	FCSSgr2

Hurka1	1	2.3614	7.3753	3.0255	9.4493	3.0	9.5	FCSSgr2
Hurka1	1	2.3632	7.3338	3.0278	9.3962	3.0	9.5	FCSSgr2
Hurka1	1	2.3973	7.2928	3.0715	9.3436	3.0	9.5	FCSSgr2
Hurka1	1	2.2976	7.8389	2.9437	10.0432	3.0	10.0	FCSSgr2
Hurka1	1	2.3006	7.6227	2.9475	9.7663	3.0	10.0	FCSSgr2
Hurka1	1	2.3077	7.7147	2.9567	9.8842	3.0	10.0	FCSSgr2
Hurka1	1	2.3104	7.9667	2.9601	10.2070	3.0	10.0	FCSSgr2
Hurka1	1	2.3126	7.8250	2.9629	10.0254	3.0	10.0	FCSSgr2
Hurka1	1	2.3139	7.6875	2.9645	9.8493	3.0	10.0	FCSSgr2
Hurka1	1	2.3148	7.7776	2.9657	9.9647	3.0	10.0	FCSSgr2
Hurka1	1	2.3151	7.7582	2.9662	9.9399	3.0	10.0	FCSSgr2
Hurka1	1	2.3157	7.7520	2.9669	9.9320	3.0	10.0	FCSSgr2
Hurka1	1	2.3158	7.7803	2.9670	9.9681	3.0	10.0	FCSSgr2
Hurka1	1	2.3159	7.7377	2.9671	9.9136	3.0	10.0	FCSSgr2
Hurka1	1	2.3203	7.7352	2.9728	9.9104	3.0	10.0	FCSSgr2
Hurka1	1	2.3205	7.9125	2.9731	10.1376	3.0	10.0	FCSSgr2
Hurka1	1	2.3234	7.7954	2.9768	9.9875	3.0	10.0	FCSSgr2
Hurka1	1	2.3242	7.6239	2.9778	9.7678	3.0	10.0	FCSSgr2
Hurka1	1	2.3246	7.7046	2.9783	9.8712	3.0	10.0	FCSSgr2
Hurka1	1	2.3251	7.6823	2.9789	9.8427	3.0	10.0	FCSSgr2
Hurka1	1	2.3251	7.7291	2.9790	9.9026	3.0	10.0	FCSSgr2
Hurka1	1	2.3294	7.7640	2.9845	9.9473	3.0	10.0	FCSSgr2
Hurka1	1	2.3296	7.7308	2.9847	9.9047	3.0	10.0	FCSSgr2
Hurka1	1	2.3313	7.7026	2.9869	9.8686	3.0	10.0	FCSSgr2
Hurka1	1	2.3330	7.8626	2.9890	10.0736	3.0	10.0	FCSSgr2
Hurka1	1	2.3357	7.7485	2.9925	9.9274	3.0	10.0	FCSSgr2
Hurka1	1	2.3373	7.7050	2.9945	9.8717	3.0	10.0	FCSSgr2
Hurka1	1	2.3373	7.7895	2.9946	9.9800	3.0	10.0	FCSSgr2
Hurka1	1	2.3389	7.7549	2.9966	9.9357	3.0	10.0	FCSSgr2
Hurka1	1	2.3410	7.6922	2.9993	9.8553	3.0	10.0	FCSSgr2
Hurka1	1	2.3417	7.7946	3.0002	9.9866	3.0	10.0	FCSSgr2
Hurka1	1	2.3437	7.7879	3.0027	9.9780	3.0	10.0	FCSSgr2
Hurka1	1	2.3441	7.6779	3.0033	9.8370	3.0	10.0	FCSSgr2

Hurka1	1	2.3456	7.7095	3.0052	9.8774	3.0	10.0	FCSSgr2
Hurka1	1	2.3479	7.7837	3.0082	9.9725	3.0	10.0	FCSSgr2
Hurka1	1	2.3495	7.6181	3.0101	9.7604	3.0	10.0	FCSSgr2
Hurka1	1	2.3495	7.8966	3.0102	10.1172	3.0	10.0	FCSSgr2
Hurka1	1	2.3517	7.8692	3.0130	10.0821	3.0	10.0	FCSSgr2
Hurka1	1	2.3549	7.7477	3.0171	9.9265	3.0	10.0	FCSSgr2
Hurka1	1	2.3565	7.7950	3.0192	9.9871	3.0	10.0	FCSSgr2
Hurka1	1	2.3581	7.6634	3.0212	9.8184	3.0	10.0	FCSSgr2
Hurka1	1	2.3626	7.7682	3.0269	9.9527	3.0	10.0	FCSSgr2
Hurka1	1	2.3684	7.7513	3.0344	9.9310	3.0	10.0	FCSSgr2
Hurka1	1	2.3701	7.8125	3.0366	10.0095	3.0	10.0	FCSSgr2
Hurka1	1	2.3975	7.8656	3.0717	10.0775	3.0	10.0	FCSSgr2
Hurka1	1	2.4278	7.9880	3.1105	10.2343	3.0	10.0	FCSSgr2
Hurka1	1	2.5196	7.9342	3.2282	10.1654	3.0	10.0	FCSSgr2
Hurka1	1	3.8753	7.8001	4.9651	9.9935	5.0	10.0	FCSSgr2
Hurka1	1	3.9242	7.7039	5.0277	9.8703	5.0	10.0	FCSSgr2
Hurka1	1	3.9408	7.7221	5.0490	9.8936	5.0	10.0	FCSSgr2
Hurka1	1	3.9953	7.8060	5.1188	10.0012	5.0	10.0	FCSSgr2
Hurka1	1	2.3118	8.0244	2.9619	10.2810	3.0	10.5	FCSSgr2
Hurka1	1	2.3799	8.0188	3.0492	10.2737	3.0	10.5	FCSSgr2
Hurka1	1	2.3908	8.0645	3.0631	10.3323	3.0	10.5	FCSSgr2
Hurka1	1	2.3720	8.5073	3.0390	10.8997	3.0	11.0	FCSSgr2
Hurka1	1	2.4296	9.8900	3.1128	12.6712	3.0	12.5	FCSSgr2
Hurka1	1	2.3030	9.9799	2.9506	12.7863	3.0	13.0	FCSSgr2
Hurka1	1	2.3215	9.9936	2.9743	12.8039	3.0	13.0	FCSSgr2
Hurka1	1	2.3368	9.9897	2.9940	12.7989	3.0	13.0	FCSSgr2
Hurka1	1	4.7841	11.2331	6.1294	14.3919	6.0	14.5	FCSSgr2
Hurka1	1	4.7540	15.3804	6.0909	19.7055	6.0	19.5	FCSSgr2
Hurka1	1	2.3084	not recorded	2.9575	NA	3.0	NA	FCSSgr2
Hurka1	1	2.3499	not recorded	3.0108	NA	3.0	NA	FCSSgr2
Hurka1	1	2.3552	not recorded	3.0175	NA	3.0	NA	FCSSgr2
Hurka1	1	2.3689	not recorded	3.0351	NA	3.0	NA	FCSSgr2
kyrtostylaA	1	3.1468	5.5118	4.0118	7.0269	4.0	7.0	FCSSgr2

kyrtostylaA	1	3.1607	5.5266	4.0295	7.0458	4.0	7.0	FCSSgr2
kyrtostylaA	1	3.4952	5.8562	4.4559	7.4660	4.5	7.5	FCSSgr2
kyrtostylaA	1	2.3665	7.3007	3.0170	9.3076	3.0	9.5	FCSSgr2
kyrtostylaA	1	2.3399	7.9302	2.9831	10.1101	3.0	10.0	FCSSgr2
RaznHura1	1	2.3889	4.1719	3.0231	5.2796	3.0	5.5	FCSSgr1
RaznHura1	1	3.1900	5.2891	4.0369	6.6935	4.0	6.5	FCSSgr1
RaznHura1	1	2.3475	5.6179	2.9708	7.1095	3.0	7.0	FCSSgr1
RaznHura1	1	2.3559	5.4969	2.9815	6.9564	3.0	7.0	FCSSgr1
RaznHura1	1	2.3560	5.5503	2.9816	7.0240	3.0	7.0	FCSSgr1
RaznHura1	1	2.3596	5.5732	2.9861	7.0530	3.0	7.0	FCSSgr1
RaznHura1	1	3.0837	5.3780	3.9024	6.8060	4.0	7.0	FCSSgr1
RaznHura1	1	3.0952	5.4191	3.9170	6.8580	4.0	7.0	FCSSgr1
RaznHura1	1	3.1009	5.4889	3.9242	6.9463	4.0	7.0	FCSSgr1
RaznHura1	1	3.1144	5.5198	3.9413	6.9854	4.0	7.0	FCSSgr1
RaznHura1	1	3.1476	5.4232	3.9834	6.8631	4.0	7.0	FCSSgr1
RaznHura1	1	3.1505	5.5546	3.9870	7.0294	4.0	7.0	FCSSgr1
RaznHura1	1	3.1506	5.4248	3.9872	6.8652	4.0	7.0	FCSSgr1
RaznHura1	1	3.1681	5.5403	4.0093	7.0113	4.0	7.0	FCSSgr1
RaznHura1	1	3.1719	5.6010	4.0141	7.0882	4.0	7.0	FCSSgr1
RaznHura1	1	3.1731	5.4961	4.0156	6.9553	4.0	7.0	FCSSgr1
RaznHura1	1	3.1816	5.6152	4.0264	7.1061	4.0	7.0	FCSSgr1
RaznHura1	1	3.1867	5.5636	4.0328	7.0408	4.0	7.0	FCSSgr1
RaznHura1	1	3.1874	5.5821	4.0337	7.0642	4.0	7.0	FCSSgr1
RaznHura1	1	3.2042	5.6015	4.0549	7.0889	4.0	7.0	FCSSgr1
RaznHura1	1	3.2349	5.6764	4.0938	7.1835	4.0	7.0	FCSSgr1
RaznHura1	1	3.2475	5.5894	4.1098	7.0735	4.0	7.0	FCSSgr1
RaznHura1	1	2.3313	6.0781	2.9503	7.6919	3.0	7.5	FCSSgr1
RaznHura1	1	2.3618	5.7546	2.9889	7.2825	3.0	7.5	FCSSgr1
RaznHura1	1	2.3767	5.9520	3.0077	7.5324	3.0	7.5	FCSSgr1
RaznHura1	1	3.1692	6.1080	4.0107	7.7298	4.0	7.5	FCSSgr1
RaznHura1	1	3.7164	6.1082	4.7032	7.7300	4.5	7.5	FCSSgr1
RaznHura1	1	2.3130	6.2342	2.9271	7.8895	3.0	8.0	FCSSgr1
RaznHura1	1	2.3169	6.2018	2.9321	7.8485	3.0	8.0	FCSSgr1

RaznHura1	1	2.3256	6.3528	2.9430	8.0395	3.0	8.0	FCSSgr1
RaznHura1	1	2.3311	6.2242	2.9500	7.8769	3.0	8.0	FCSSgr1
RaznHura1	1	2.3325	6.3028	2.9518	7.9763	3.0	8.0	FCSSgr1
RaznHura1	1	2.3346	6.3365	2.9545	8.0189	3.0	8.0	FCSSgr1
RaznHura1	1	2.3354	6.2368	2.9555	7.8927	3.0	8.0	FCSSgr1
RaznHura1	1	2.3595	6.1833	2.9859	7.8250	3.0	8.0	FCSSgr1
RaznHura1	1	2.3596	6.2025	2.9861	7.8493	3.0	8.0	FCSSgr1
RaznHura1	1	2.3599	6.3328	2.9864	8.0143	3.0	8.0	FCSSgr1
RaznHura1	1	2.3616	6.2707	2.9887	7.9357	3.0	8.0	FCSSgr1
RaznHura1	1	2.3620	6.3386	2.9891	8.0217	3.0	8.0	FCSSgr1
RaznHura1	1	2.3626	6.2630	2.9900	7.9259	3.0	8.0	FCSSgr1
RaznHura1	1	2.3630	6.3607	2.9904	8.0496	3.0	8.0	FCSSgr1
RaznHura1	1	2.3633	6.3794	2.9907	8.0732	3.0	8.0	FCSSgr1
RaznHura1	1	2.3642	6.2054	2.9919	7.8530	3.0	8.0	FCSSgr1
RaznHura1	1	2.3647	6.3236	2.9925	8.0027	3.0	8.0	FCSSgr1
RaznHura1	1	2.3652	6.1906	2.9932	7.8343	3.0	8.0	FCSSgr1
RaznHura1	1	2.3654	6.3795	2.9935	8.0734	3.0	8.0	FCSSgr1
RaznHura1	1	2.3684	6.3338	2.9972	8.0155	3.0	8.0	FCSSgr1
RaznHura1	1	2.3699	6.3667	2.9992	8.0571	3.0	8.0	FCSSgr1
RaznHura1	1	2.3707	6.2257	3.0002	7.8788	3.0	8.0	FCSSgr1
RaznHura1	1	2.3710	6.3832	3.0005	8.0780	3.0	8.0	FCSSgr1
RaznHura1	1	2.3715	6.3076	3.0012	7.9823	3.0	8.0	FCSSgr1
RaznHura1	1	2.3716	6.2686	3.0013	7.9331	3.0	8.0	FCSSgr1
RaznHura1	1	2.3729	6.2965	3.0030	7.9684	3.0	8.0	FCSSgr1
RaznHura1	1	2.3741	6.3580	3.0044	8.0462	3.0	8.0	FCSSgr1
RaznHura1	1	2.3742	6.4181	3.0045	8.1222	3.0	8.0	FCSSgr1
RaznHura1	1	2.3750	6.3823	3.0056	8.0769	3.0	8.0	FCSSgr1
RaznHura1	1	2.3754	6.3598	3.0060	8.0484	3.0	8.0	FCSSgr1
RaznHura1	1	2.3754	6.4267	3.0061	8.1331	3.0	8.0	FCSSgr1
RaznHura1	1	2.3761	6.3406	3.0070	8.0242	3.0	8.0	FCSSgr1
RaznHura1	1	2.3763	6.3990	3.0072	8.0980	3.0	8.0	FCSSgr1
RaznHura1	1	2.3769	6.3364	3.0079	8.0189	3.0	8.0	FCSSgr1
RaznHura1	1	2.3785	6.4700	3.0101	8.1879	3.0	8.0	FCSSgr1

RaznHura1	1	2.3792	6.2924	3.0109	7.9631	3.0	8.0	FCSSgr1
RaznHura1	1	2.3794	6.3530	3.0112	8.0398	3.0	8.0	FCSSgr1
RaznHura1	1	2.3807	6.4026	3.0128	8.1026	3.0	8.0	FCSSgr1
RaznHura1	1	2.3840	6.2582	3.0170	7.9199	3.0	8.0	FCSSgr1
RaznHura1	1	2.3851	6.2302	3.0184	7.8844	3.0	8.0	FCSSgr1
RaznHura1	1	2.3854	6.2525	3.0188	7.9127	3.0	8.0	FCSSgr1
RaznHura1	1	2.3855	6.3994	3.0189	8.0985	3.0	8.0	FCSSgr1
RaznHura1	1	2.3863	6.3460	3.0200	8.0310	3.0	8.0	FCSSgr1
RaznHura1	1	2.3867	6.3982	3.0204	8.0971	3.0	8.0	FCSSgr1
RaznHura1	1	2.3898	6.4209	3.0243	8.1258	3.0	8.0	FCSSgr1
RaznHura1	1	2.3932	6.3648	3.0286	8.0547	3.0	8.0	FCSSgr1
RaznHura1	1	2.4492	6.5044	3.0995	8.2314	3.0	8.0	FCSSgr1
RaznHura1	1	3.1691	6.3511	4.0105	8.0375	4.0	8.0	FCSSgr1
RaznHura1	1	3.1814	6.3968	4.0261	8.0953	4.0	8.0	FCSSgr1
RaznHura1	1	3.2013	6.3198	4.0513	7.9979	4.0	8.0	FCSSgr1
RaznHura1	1	3.8341	6.2599	4.8521	7.9221	5.0	8.0	FCSSgr1
RaznHura1	1	2.4769	6.7084	3.1345	8.4895	3.0	8.5	FCSSgr1
RaznHura1	1	2.4973	6.5980	3.1603	8.3499	3.0	8.5	FCSSgr1
RaznHura1	1	3.2619	6.5206	4.1280	8.2519	4.0	8.5	FCSSgr1
RaznHura1	1	2.3390	7.1005	2.9601	8.9858	3.0	9.0	FCSSgr1
RaznHura1	1	2.3699	7.1633	2.9991	9.0653	3.0	9.0	FCSSgr1
RaznHura1	1	2.3820	6.9730	3.0145	8.8245	3.0	9.0	FCSSgr1
RaznHura1	1	2.3830	7.0044	3.0157	8.8642	3.0	9.0	FCSSgr1
RaznHura1	1	2.3910	7.2555	3.0259	9.1820	3.0	9.0	FCSSgr1
RaznHura1	1	2.3317	7.3843	2.9508	9.3449	3.0	9.5	FCSSgr1
RaznHura1	1	2.3612	7.3203	2.9882	9.2640	3.0	9.5	FCSSgr1
RaznHura1	1	2.3642	7.3665	2.9919	9.3224	3.0	9.5	FCSSgr1
RaznHura1	1	2.3752	7.3969	3.0059	9.3609	3.0	9.5	FCSSgr1
RaznHura1	1	2.3756	7.5813	3.0064	9.5942	3.0	9.5	FCSSgr1
RaznHura1	1	3.2268	7.5970	4.0836	9.6141	4.0	9.5	FCSSgr1
RaznHura1	1	2.3773	7.7422	3.0085	9.7978	3.0	10.0	FCSSgr1
RaznHura1	1	3.6810	8.4083	4.6583	10.6409	4.5	10.5	FCSSgr1
RaznHura1	1	2.3655	8.6758	2.9936	10.9793	3.0	11.0	FCSSgr1

RaznHura1	1	3.1690	not recorded	4.0104	NA	4.0	NA	FCSSgr1
RaznHura2	1	2.2973	5.4966	2.9137	6.9715	3.0	7.0	FCSSgr1
RaznHura2	1	2.3445	5.5510	2.9736	7.0404	3.0	7.0	FCSSgr1
RaznHura2	1	2.3523	5.5157	2.9834	6.9956	3.0	7.0	FCSSgr1
RaznHura2	1	2.3536	5.3936	2.9851	6.8408	3.0	7.0	FCSSgr1
RaznHura2	1	2.3537	5.4824	2.9852	6.9534	3.0	7.0	FCSSgr1
RaznHura2	1	2.3562	5.5199	2.9885	7.0010	3.0	7.0	FCSSgr1
RaznHura2	1	2.3579	5.4699	2.9905	6.9375	3.0	7.0	FCSSgr1
RaznHura2	1	2.3587	5.5360	2.9916	7.0214	3.0	7.0	FCSSgr1
RaznHura2	1	2.3593	5.5316	2.9923	7.0159	3.0	7.0	FCSSgr1
RaznHura2	1	2.3601	5.5302	2.9934	7.0140	3.0	7.0	FCSSgr1
RaznHura2	1	2.3685	5.5347	3.0040	7.0197	3.0	7.0	FCSSgr1
RaznHura2	1	2.3691	5.5916	3.0048	7.0919	3.0	7.0	FCSSgr1
RaznHura2	1	2.3693	5.5459	3.0050	7.0339	3.0	7.0	FCSSgr1
RaznHura2	1	2.3713	5.5347	3.0075	7.0198	3.0	7.0	FCSSgr1
RaznHura2	1	2.3727	5.5863	3.0094	7.0852	3.0	7.0	FCSSgr1
RaznHura2	1	2.3728	5.5869	3.0094	7.0860	3.0	7.0	FCSSgr1
RaznHura2	1	2.3732	5.5598	3.0099	7.0516	3.0	7.0	FCSSgr1
RaznHura2	1	2.3734	5.5484	3.0103	7.0372	3.0	7.0	FCSSgr1
RaznHura2	1	2.3818	5.3940	3.0209	6.8414	3.0	7.0	FCSSgr1
RaznHura2	1	2.4053	5.5982	3.0507	7.1002	3.0	7.0	FCSSgr1
RaznHura2	1	3.1531	5.5157	3.9991	6.9956	4.0	7.0	FCSSgr1
RaznHura2	1	3.1589	5.4804	4.0064	6.9509	4.0	7.0	FCSSgr1
RaznHura2	1	3.1695	5.5886	4.0200	7.0881	4.0	7.0	FCSSgr1
RaznHura2	1	3.1876	5.6080	4.0429	7.1128	4.0	7.0	FCSSgr1
RaznHura2	1	3.1909	5.5537	4.0471	7.0439	4.0	7.0	FCSSgr1
RaznHura2	1	2.3090	6.0371	2.9285	7.6570	3.0	7.5	FCSSgr1
RaznHura2	1	2.3157	6.2008	2.9371	7.8646	3.0	8.0	FCSSgr1
RaznHura2	1	2.3287	6.1343	2.9535	7.7802	3.0	8.0	FCSSgr1
RaznHura2	1	2.3460	6.3084	2.9754	8.0011	3.0	8.0	FCSSgr1
RaznHura2	1	2.3476	6.2831	2.9775	7.9689	3.0	8.0	FCSSgr1
RaznHura2	1	2.3484	6.2954	2.9785	7.9846	3.0	8.0	FCSSgr1
RaznHura2	1	2.3508	6.2084	2.9815	7.8742	3.0	8.0	FCSSgr1

RaznHura2	1	2.3515	6.2326	2.9824	7.9049	3.0	8.0	FCSSgr1
RaznHura2	1	2.3517	6.2959	2.9827	7.9852	3.0	8.0	FCSSgr1
RaznHura2	1	2.3522	6.2086	2.9833	7.8744	3.0	8.0	FCSSgr1
RaznHura2	1	2.3528	6.3267	2.9841	8.0242	3.0	8.0	FCSSgr1
RaznHura2	1	2.3537	6.3006	2.9853	7.9912	3.0	8.0	FCSSgr1
RaznHura2	1	2.3540	6.2919	2.9856	7.9802	3.0	8.0	FCSSgr1
RaznHura2	1	2.3562	6.4099	2.9883	8.1298	3.0	8.0	FCSSgr1
RaznHura2	1	2.3562	6.3670	2.9884	8.0754	3.0	8.0	FCSSgr1
RaznHura2	1	2.3573	6.2879	2.9898	7.9751	3.0	8.0	FCSSgr1
RaznHura2	1	2.3588	6.3129	2.9917	8.0068	3.0	8.0	FCSSgr1
RaznHura2	1	2.3591	6.3083	2.9922	8.0009	3.0	8.0	FCSSgr1
RaznHura2	1	2.3597	6.3684	2.9929	8.0772	3.0	8.0	FCSSgr1
RaznHura2	1	2.3609	6.2943	2.9944	7.9832	3.0	8.0	FCSSgr1
RaznHura2	1	2.3611	6.3395	2.9946	8.0405	3.0	8.0	FCSSgr1
RaznHura2	1	2.3621	6.3219	2.9959	8.0182	3.0	8.0	FCSSgr1
RaznHura2	1	2.3625	6.3267	2.9965	8.0242	3.0	8.0	FCSSgr1
RaznHura2	1	2.3628	6.3255	2.9968	8.0228	3.0	8.0	FCSSgr1
RaznHura2	1	2.3631	6.3144	2.9972	8.0087	3.0	8.0	FCSSgr1
RaznHura2	1	2.3634	6.3148	2.9976	8.0092	3.0	8.0	FCSSgr1
RaznHura2	1	2.3635	6.3201	2.9977	8.0159	3.0	8.0	FCSSgr1
RaznHura2	1	2.3648	6.2656	2.9993	7.9468	3.0	8.0	FCSSgr1
RaznHura2	1	2.3653	6.4647	2.9999	8.1993	3.0	8.0	FCSSgr1
RaznHura2	1	2.3654	6.3261	3.0001	8.0235	3.0	8.0	FCSSgr1
RaznHura2	1	2.3655	6.3136	3.0002	8.0077	3.0	8.0	FCSSgr1
RaznHura2	1	2.3657	6.2940	3.0005	7.9828	3.0	8.0	FCSSgr1
RaznHura2	1	2.3659	6.3333	3.0008	8.0326	3.0	8.0	FCSSgr1
RaznHura2	1	2.3664	6.3560	3.0013	8.0614	3.0	8.0	FCSSgr1
RaznHura2	1	2.3667	6.3091	3.0017	8.0020	3.0	8.0	FCSSgr1
RaznHura2	1	2.3669	6.3274	3.0020	8.0251	3.0	8.0	FCSSgr1
RaznHura2	1	2.3670	6.3103	3.0021	8.0034	3.0	8.0	FCSSgr1
RaznHura2	1	2.3673	6.3452	3.0024	8.0477	3.0	8.0	FCSSgr1
RaznHura2	1	2.3675	6.3406	3.0028	8.0419	3.0	8.0	FCSSgr1
RaznHura2	1	2.3676	6.2280	3.0028	7.8991	3.0	8.0	FCSSgr1

RaznHura2	1	2.3692	6.2852	3.0049	7.9716	3.0	8.0	FCSSgr1
RaznHura2	1	2.3702	6.3540	3.0061	8.0589	3.0	8.0	FCSSgr1
RaznHura2	1	2.3706	6.3585	3.0067	8.0646	3.0	8.0	FCSSgr1
RaznHura2	1	2.3707	6.3524	3.0068	8.0569	3.0	8.0	FCSSgr1
RaznHura2	1	2.3730	6.3679	3.0097	8.0765	3.0	8.0	FCSSgr1
RaznHura2	1	2.3738	6.3099	3.0107	8.0029	3.0	8.0	FCSSgr1
RaznHura2	1	2.3747	6.4063	3.0118	8.1252	3.0	8.0	FCSSgr1
RaznHura2	1	2.3754	6.2626	3.0127	7.9430	3.0	8.0	FCSSgr1
RaznHura2	1	2.3758	6.3050	3.0132	7.9968	3.0	8.0	FCSSgr1
RaznHura2	1	2.3767	6.3538	3.0144	8.0586	3.0	8.0	FCSSgr1
RaznHura2	1	2.3773	6.3018	3.0152	7.9927	3.0	8.0	FCSSgr1
RaznHura2	1	2.3779	6.3163	3.0160	8.0111	3.0	8.0	FCSSgr1
RaznHura2	1	2.3782	6.3487	3.0163	8.0522	3.0	8.0	FCSSgr1
RaznHura2	1	2.3785	6.2924	3.0167	7.9808	3.0	8.0	FCSSgr1
RaznHura2	1	2.3817	6.3878	3.0207	8.1018	3.0	8.0	FCSSgr1
RaznHura2	1	2.3825	6.3149	3.0218	8.0093	3.0	8.0	FCSSgr1
RaznHura2	1	2.3829	6.3577	3.0223	8.0636	3.0	8.0	FCSSgr1
RaznHura2	1	2.3834	6.4562	3.0229	8.1885	3.0	8.0	FCSSgr1
RaznHura2	1	2.3835	6.3309	3.0230	8.0296	3.0	8.0	FCSSgr1
RaznHura2	1	2.3886	6.3220	3.0295	8.0183	3.0	8.0	FCSSgr1
RaznHura2	1	2.3944	6.3200	3.0369	8.0157	3.0	8.0	FCSSgr1
RaznHura2	1	2.3954	6.3615	3.0382	8.0683	3.0	8.0	FCSSgr1
RaznHura2	1	2.3992	6.4315	3.0429	8.1571	3.0	8.0	FCSSgr1
RaznHura2	1	3.1610	6.3261	4.0091	8.0235	4.0	8.0	FCSSgr1
RaznHura2	1	3.2271	6.3621	4.0930	8.0691	4.0	8.0	FCSSgr1
RaznHura2	1	2.4307	6.5276	3.0828	8.2791	3.0	8.5	FCSSgr1
RaznHura2	1	2.3817	7.3130	3.0208	9.2752	3.0	9.5	FCSSgr1
RaznHura2	1	2.3428	7.6892	2.9714	9.7523	3.0	10.0	FCSSgr1
RaznHura2	1	2.3503	7.9388	2.9809	10.0690	3.0	10.0	FCSSgr1
RaznHura2	1	2.3523	7.7842	2.9835	9.8729	3.0	10.0	FCSSgr1
RaznHura2	1	2.3719	7.7624	3.0083	9.8452	3.0	10.0	FCSSgr1
RaznHura2	1	2.3654	8.6590	3.0001	10.9823	3.0	11.0	FCSSgr1
RaznHura2	1	2.3749	8.7239	3.0121	11.0646	3.0	11.0	FCSSgr1

RaznHura2	1	2.3865	8.6751	3.0268	11.0027	3.0	11.0	FCSSgr1
RaznHura2	1	4.7342	10.9332	6.0045	13.8667	6.0	14.0	FCSSgr1
RaznHura2	1	2.3629	not recorded	2.9969	NA	3.0	NA	FCSSgr1
RaznHura2	1	2.3645	not recorded	2.9989	NA	3.0	NA	FCSSgr1
RaznHura2	1	2.3794	not recorded	3.0178	NA	3.0	NA	FCSSgr1
RaznHura2	1	3.1717	not recorded	4.0228	NA	4.0	NA	FCSSgr1
Sosen3	1	2.2958	4.6286	2.9880	6.0243	3.0	6.0	FCSSgr2
Sosen3	1	2.4467	4.9065	3.1845	6.3860	3.0	6.5	FCSSgr2
Sosen3	1	2.2910	5.8515	2.9818	7.6159	3.0	7.5	FCSSgr2
Sosen3	1	2.2937	5.8507	2.9853	7.6148	3.0	7.5	FCSSgr2
Sosen3	1	2.2953	5.9307	2.9874	7.7190	3.0	7.5	FCSSgr2
Sosen3	1	2.2958	5.9198	2.9881	7.7049	3.0	7.5	FCSSgr2
Sosen3	1	2.2995	5.8218	2.9928	7.5773	3.0	7.5	FCSSgr2
Sosen3	1	2.2999	5.7428	2.9934	7.4745	3.0	7.5	FCSSgr2
Sosen3	1	2.3040	5.8115	2.9987	7.5638	3.0	7.5	FCSSgr2
Sosen3	1	2.3044	5.8948	2.9992	7.6723	3.0	7.5	FCSSgr2
Sosen3	1	2.3049	5.9475	2.9999	7.7408	3.0	7.5	FCSSgr2
Sosen3	1	2.3159	5.9244	3.0142	7.7108	3.0	7.5	FCSSgr2
Sosen3	1	2.3235	5.9464	3.0241	7.7395	3.0	7.5	FCSSgr2
Sosen3	1	2.2863	6.1163	2.9757	7.9606	3.0	8.0	FCSSgr2
Sosen3	1	2.2927	6.0168	2.9840	7.8310	3.0	8.0	FCSSgr2
Sosen3	1	2.2944	6.1986	2.9863	8.0676	3.0	8.0	FCSSgr2
Sosen3	1	2.2980	6.0017	2.9909	7.8114	3.0	8.0	FCSSgr2
Sosen3	1	2.3020	5.9644	2.9961	7.7629	3.0	8.0	FCSSgr2
Sosen3	1	2.3071	6.0050	3.0028	7.8157	3.0	8.0	FCSSgr2
Sosen3	1	2.3087	6.0484	3.0048	7.8722	3.0	8.0	FCSSgr2
Sosen3	1	2.3090	5.9916	3.0052	7.7983	3.0	8.0	FCSSgr2
Sosen3	1	2.3130	5.9802	3.0104	7.7834	3.0	8.0	FCSSgr2
Sosen3	1	2.3335	5.9655	3.0372	7.7644	3.0	8.0	FCSSgr2
Sosen3	1	2.3799	6.1010	3.0975	7.9407	3.0	8.0	FCSSgr2
Sosen3	1	3.7128	5.9781	4.8323	7.7807	5.0	8.0	FCSSgr2
Sosen3	1	2.2859	6.6427	2.9752	8.6456	3.0	8.5	FCSSgr2
Sosen3	1	2.2969	6.4865	2.9895	8.4424	3.0	8.5	FCSSgr2

Sosen3	1	2.2970	6.7185	2.9897	8.7443	3.0	8.5	FCSSgr2
Sosen3	1	2.3123	6.3928	3.0096	8.3205	3.0	8.5	FCSSgr2
Sosen3	1	2.2774	6.9822	2.9642	9.0875	3.0	9.0	FCSSgr2
Sosen3	1	2.2839	6.8993	2.9725	8.9796	3.0	9.0	FCSSgr2
Sosen3	1	2.2872	6.9258	2.9769	9.0141	3.0	9.0	FCSSgr2
Sosen3	1	2.2919	6.8783	2.9829	8.9523	3.0	9.0	FCSSgr2
Sosen3	1	2.2933	6.8180	2.9849	8.8739	3.0	9.0	FCSSgr2
Sosen3	1	2.2934	7.0327	2.9849	9.1533	3.0	9.0	FCSSgr2
Sosen3	1	2.2934	6.9894	2.9850	9.0969	3.0	9.0	FCSSgr2
Sosen3	1	2.2957	7.0965	2.9879	9.2364	3.0	9.0	FCSSgr2
Sosen3	1	2.2963	6.8898	2.9887	8.9673	3.0	9.0	FCSSgr2
Sosen3	1	2.2964	6.9069	2.9889	8.9895	3.0	9.0	FCSSgr2
Sosen3	1	2.2964	6.9380	2.9889	9.0300	3.0	9.0	FCSSgr2
Sosen3	1	2.2968	7.1053	2.9894	9.2478	3.0	9.0	FCSSgr2
Sosen3	1	2.2985	7.0710	2.9916	9.2031	3.0	9.0	FCSSgr2
Sosen3	1	2.2988	7.0882	2.9920	9.2255	3.0	9.0	FCSSgr2
Sosen3	1	2.2990	6.8976	2.9922	8.9774	3.0	9.0	FCSSgr2
Sosen3	1	2.2996	7.0471	2.9930	9.1721	3.0	9.0	FCSSgr2
Sosen3	1	2.3036	7.0613	2.9982	9.1906	3.0	9.0	FCSSgr2
Sosen3	1	2.3040	7.0640	2.9988	9.1940	3.0	9.0	FCSSgr2
Sosen3	1	2.3045	6.9730	2.9994	9.0756	3.0	9.0	FCSSgr2
Sosen3	1	2.3055	6.9931	3.0007	9.1017	3.0	9.0	FCSSgr2
Sosen3	1	2.3063	6.9799	3.0018	9.0846	3.0	9.0	FCSSgr2
Sosen3	1	2.3081	7.1021	3.0041	9.2436	3.0	9.0	FCSSgr2
Sosen3	1	2.3104	6.9525	3.0070	9.0489	3.0	9.0	FCSSgr2
Sosen3	1	2.3142	7.0835	3.0120	9.2194	3.0	9.0	FCSSgr2
Sosen3	1	2.3266	6.7796	3.0281	8.8239	3.0	9.0	FCSSgr2
Sosen3	1	2.3289	6.8562	3.0311	8.9236	3.0	9.0	FCSSgr2
Sosen3	1	2.3392	6.8464	3.0445	8.9108	3.0	9.0	FCSSgr2
Sosen3	1	2.2748	7.2455	2.9607	9.4303	3.0	9.5	FCSSgr2
Sosen3	1	2.2799	7.3233	2.9674	9.5315	3.0	9.5	FCSSgr2
Sosen3	1	2.2838	7.3273	2.9725	9.5367	3.0	9.5	FCSSgr2
Sosen3	1	2.2840	7.2516	2.9727	9.4381	3.0	9.5	FCSSgr2

Sosen3	1	2.2842	7.1772	2.9730	9.3414	3.0	9.5	FCSSgr2
Sosen3	1	2.2849	7.1389	2.9739	9.2915	3.0	9.5	FCSSgr2
Sosen3	1	2.2875	7.2455	2.9772	9.4303	3.0	9.5	FCSSgr2
Sosen3	1	2.2890	7.1507	2.9792	9.3069	3.0	9.5	FCSSgr2
Sosen3	1	2.2900	7.3235	2.9805	9.5318	3.0	9.5	FCSSgr2
Sosen3	1	2.2907	7.1142	2.9815	9.2594	3.0	9.5	FCSSgr2
Sosen3	1	2.2911	7.2177	2.9819	9.3941	3.0	9.5	FCSSgr2
Sosen3	1	2.2920	7.3064	2.9831	9.5095	3.0	9.5	FCSSgr2
Sosen3	1	2.2922	7.3674	2.9834	9.5889	3.0	9.5	FCSSgr2
Sosen3	1	2.2927	7.2053	2.9840	9.3779	3.0	9.5	FCSSgr2
Sosen3	1	2.2934	7.1984	2.9849	9.3689	3.0	9.5	FCSSgr2
Sosen3	1	2.2942	7.1929	2.9859	9.3618	3.0	9.5	FCSSgr2
Sosen3	1	2.2945	7.3824	2.9863	9.6084	3.0	9.5	FCSSgr2
Sosen3	1	2.2946	7.2102	2.9865	9.3843	3.0	9.5	FCSSgr2
Sosen3	1	2.2947	7.4100	2.9866	9.6444	3.0	9.5	FCSSgr2
Sosen3	1	2.2950	7.2755	2.9870	9.4694	3.0	9.5	FCSSgr2
Sosen3	1	2.2950	7.2105	2.9871	9.3847	3.0	9.5	FCSSgr2
Sosen3	1	2.2952	7.1182	2.9873	9.2646	3.0	9.5	FCSSgr2
Sosen3	1	2.2952	7.2643	2.9873	9.4547	3.0	9.5	FCSSgr2
Sosen3	1	2.2953	7.3369	2.9874	9.5493	3.0	9.5	FCSSgr2
Sosen3	1	2.2953	7.2890	2.9875	9.4869	3.0	9.5	FCSSgr2
Sosen3	1	2.2963	7.2676	2.9888	9.4590	3.0	9.5	FCSSgr2
Sosen3	1	2.2964	7.3257	2.9889	9.5346	3.0	9.5	FCSSgr2
Sosen3	1	2.2973	7.1238	2.9901	9.2718	3.0	9.5	FCSSgr2
Sosen3	1	2.2983	7.4479	2.9913	9.6936	3.0	9.5	FCSSgr2
Sosen3	1	2.2986	7.2081	2.9918	9.3816	3.0	9.5	FCSSgr2
Sosen3	1	2.2989	7.1618	2.9921	9.3213	3.0	9.5	FCSSgr2
Sosen3	1	2.2991	7.2775	2.9924	9.4719	3.0	9.5	FCSSgr2
Sosen3	1	2.2997	7.2321	2.9931	9.4128	3.0	9.5	FCSSgr2
Sosen3	1	2.2997	7.2432	2.9931	9.4272	3.0	9.5	FCSSgr2
Sosen3	1	2.3005	7.2449	2.9942	9.4295	3.0	9.5	FCSSgr2
Sosen3	1	2.3007	7.2406	2.9945	9.4239	3.0	9.5	FCSSgr2
Sosen3	1	2.3012	7.2874	2.9951	9.4848	3.0	9.5	FCSSgr2

Sosen3	1	2.3017	7.3595	2.9958	9.5787	3.0	9.5	FCSSgr2
Sosen3	1	2.3019	7.4499	2.9960	9.6963	3.0	9.5	FCSSgr2
Sosen3	1	2.3020	7.2458	2.9961	9.4307	3.0	9.5	FCSSgr2
Sosen3	1	2.3031	7.2798	2.9976	9.4749	3.0	9.5	FCSSgr2
Sosen3	1	2.3035	7.2079	2.9981	9.3813	3.0	9.5	FCSSgr2
Sosen3	1	2.3037	7.3653	2.9984	9.5862	3.0	9.5	FCSSgr2
Sosen3	1	2.3046	7.4604	2.9996	9.7099	3.0	9.5	FCSSgr2
Sosen3	1	2.3056	7.1549	3.0008	9.3124	3.0	9.5	FCSSgr2
Sosen3	1	2.3068	7.4643	3.0024	9.7150	3.0	9.5	FCSSgr2
Sosen3	1	2.3075	7.1683	3.0033	9.3298	3.0	9.5	FCSSgr2
Sosen3	1	2.3075	7.3487	3.0033	9.5646	3.0	9.5	FCSSgr2
Sosen3	1	2.3076	7.3129	3.0035	9.5179	3.0	9.5	FCSSgr2
Sosen3	1	2.3085	7.4808	3.0046	9.7365	3.0	9.5	FCSSgr2
Sosen3	1	2.3089	7.1904	3.0051	9.3586	3.0	9.5	FCSSgr2
Sosen3	1	2.3093	7.1886	3.0056	9.3563	3.0	9.5	FCSSgr2
Sosen3	1	2.3093	7.3889	3.0056	9.6169	3.0	9.5	FCSSgr2
Sosen3	1	2.3116	7.1489	3.0086	9.3045	3.0	9.5	FCSSgr2
Sosen3	1	2.3120	7.4523	3.0092	9.6994	3.0	9.5	FCSSgr2
Sosen3	1	2.3133	7.2588	3.0109	9.4476	3.0	9.5	FCSSgr2
Sosen3	1	2.3149	7.1253	3.0129	9.2739	3.0	9.5	FCSSgr2
Sosen3	1	2.3180	7.3484	3.0170	9.5641	3.0	9.5	FCSSgr2
Sosen3	1	2.3235	7.1423	3.0241	9.2960	3.0	9.5	FCSSgr2
Sosen3	1	2.3438	7.1611	3.0505	9.3204	3.0	9.5	FCSSgr2
Sosen3	1	2.3530	7.4840	3.0625	9.7407	3.0	9.5	FCSSgr2
Sosen3	1	2.2899	7.5190	2.9804	9.7862	3.0	10.0	FCSSgr2
Sosen3	1	2.2965	7.6516	2.9890	9.9588	3.0	10.0	FCSSgr2
Sosen3	1	2.3093	7.4950	3.0057	9.7549	3.0	10.0	FCSSgr2
Sosen3	1	2.3147	7.5344	3.0127	9.8062	3.0	10.0	FCSSgr2
Sosen3	1	2.3666	7.7358	3.0803	10.0684	3.0	10.0	FCSSgr2
Sosen3	1	2.3881	7.5105	3.1082	9.7751	3.0	10.0	FCSSgr2
Sosen3	1	2.4270	7.7105	3.1588	10.0354	3.0	10.0	FCSSgr2
Sosen3	1	2.2888	8.0347	2.9790	10.4574	3.0	10.5	FCSSgr2
Sosen3	1	2.2916	8.0395	2.9826	10.4637	3.0	10.5	FCSSgr2

Sosen3	1	2.2994	8.2203	2.9928	10.6990	3.0	10.5	FCSSgr2
Sosen3	1	2.3089	8.1281	3.0051	10.5790	3.0	10.5	FCSSgr2
Sosen3	1	2.2978	8.9806	2.9906	11.6886	3.0	11.5	FCSSgr2
Sosen3	1	2.2997	9.0926	2.9932	11.8343	3.0	12.0	FCSSgr2
Sosen3	1	2.3093	9.4797	3.0056	12.3381	3.0	12.5	FCSSgr2
Sosen3	1	2.3002	11.6438	2.9938	15.1547	3.0	15.0	FCSSgr2
Sosen3	1	4.6844	12.0907	6.0969	15.7365	6.0	15.5	FCSSgr2
Sosen3	1	2.2901	not recorded	2.9807	NA	3.0	NA	FCSSgr2
Sosen3	1	2.2966	not recorded	2.9891	NA	3.0	NA	FCSSgr2
Vyhon3	1	2.6772	3.7818	3.4093	4.8161	3.5	5.0	FCSSgr2
Vyhon3	1	2.3380	5.5495	2.9773	7.0672	3.0	7.0	FCSSgr2
Vyhon3	1	2.3394	5.6100	2.9792	7.1441	3.0	7.0	FCSSgr2
Vyhon3	1	2.3443	5.5655	2.9854	7.0874	3.0	7.0	FCSSgr2
Vyhon3	1	2.3204	6.0120	2.9550	7.6561	3.0	7.5	FCSSgr2
Vyhon3	1	2.3375	6.0012	2.9767	7.6424	3.0	7.5	FCSSgr2
Vyhon3	1	2.3401	5.9480	2.9800	7.5746	3.0	7.5	FCSSgr2
Vyhon3	1	2.3472	5.9821	2.9891	7.6181	3.0	7.5	FCSSgr2
Vyhon3	1	2.3524	6.0402	2.9957	7.6921	3.0	7.5	FCSSgr2
Vyhon3	1	2.3537	5.8785	2.9974	7.4860	3.0	7.5	FCSSgr2
Vyhon3	1	2.3553	6.0266	2.9993	7.6747	3.0	7.5	FCSSgr2
Vyhon3	1	2.3319	6.2843	2.9696	8.0029	3.0	8.0	FCSSgr2
Vyhon3	1	2.3334	6.2932	2.9716	8.0142	3.0	8.0	FCSSgr2
Vyhon3	1	2.3350	6.2984	2.9736	8.0208	3.0	8.0	FCSSgr2
Vyhon3	1	2.3409	6.4366	2.9811	8.1968	3.0	8.0	FCSSgr2
Vyhon3	1	2.3438	6.2862	2.9848	8.0053	3.0	8.0	FCSSgr2
Vyhon3	1	2.3458	6.3363	2.9873	8.0690	3.0	8.0	FCSSgr2
Vyhon3	1	2.3478	6.3626	2.9899	8.1025	3.0	8.0	FCSSgr2
Vyhon3	1	2.3481	6.4259	2.9902	8.1832	3.0	8.0	FCSSgr2
Vyhon3	1	2.3508	6.3500	2.9936	8.0866	3.0	8.0	FCSSgr2
Vyhon3	1	2.3531	6.3476	2.9966	8.0834	3.0	8.0	FCSSgr2
Vyhon3	1	2.3561	6.3288	3.0004	8.0595	3.0	8.0	FCSSgr2
Vyhon3	1	2.3562	6.1806	3.0006	7.8708	3.0	8.0	FCSSgr2
Vyhon3	1	2.3572	6.3076	3.0018	8.0326	3.0	8.0	FCSSgr2

Vyhon3	1	2.3660	6.3875	3.0130	8.1342	3.0	8.0	FCSSgr2
Vyhon3	1	2.3744	6.2550	3.0237	7.9655	3.0	8.0	FCSSgr2
Vyhon3	1	2.3929	6.3673	3.0473	8.1085	3.0	8.0	FCSSgr2
Vyhon3	1	2.3983	6.3683	3.0541	8.1098	3.0	8.0	FCSSgr2
Vyhon3	1	2.3986	6.3748	3.0546	8.1181	3.0	8.0	FCSSgr2
Vyhon3	1	2.4005	6.4575	3.0569	8.2234	3.0	8.0	FCSSgr2
Vyhon3	1	2.3357	6.5654	2.9744	8.3608	3.0	8.5	FCSSgr2
Vyhon3	1	2.3384	6.5565	2.9779	8.3496	3.0	8.5	FCSSgr2
Vyhon3	1	2.3521	6.7861	2.9953	8.6419	3.0	8.5	FCSSgr2
Vyhon3	1	2.3523	6.8530	2.9956	8.7271	3.0	8.5	FCSSgr2
Vyhon3	1	2.4118	6.4795	3.0714	8.2515	3.0	8.5	FCSSgr2
Vyhon3	1	2.3020	7.1362	2.9315	9.0877	3.0	9.0	FCSSgr2
Vyhon3	1	2.3413	6.8784	2.9816	8.7595	3.0	9.0	FCSSgr2
Vyhon3	1	2.3460	6.8717	2.9876	8.7509	3.0	9.0	FCSSgr2
Vyhon3	1	2.3495	7.0647	2.9920	8.9967	3.0	9.0	FCSSgr2
Vyhon3	1	2.3573	7.2339	3.0020	9.2121	3.0	9.0	FCSSgr2
Vyhon3	1	2.3363	7.5731	2.9752	9.6442	3.0	9.5	FCSSgr2
Vyhon3	1	2.3395	7.5055	2.9793	9.5581	3.0	9.5	FCSSgr2
Vyhon3	1	2.3408	7.3559	2.9810	9.3675	3.0	9.5	FCSSgr2
Vyhon3	1	2.3423	7.3153	2.9829	9.3158	3.0	9.5	FCSSgr2
Vyhon3	1	2.3450	7.3598	2.9863	9.3725	3.0	9.5	FCSSgr2
Vyhon3	1	2.3267	7.8916	2.9630	10.0498	3.0	10.0	FCSSgr2
Vyhon3	1	2.3316	7.8114	2.9692	9.9476	3.0	10.0	FCSSgr2
Vyhon3	1	2.3351	7.8031	2.9737	9.9370	3.0	10.0	FCSSgr2
Vyhon3	1	2.3353	7.7114	2.9740	9.8202	3.0	10.0	FCSSgr2
Vyhon3	1	2.3363	7.8673	2.9753	10.0188	3.0	10.0	FCSSgr2
Vyhon3	1	2.3364	7.7525	2.9753	9.8726	3.0	10.0	FCSSgr2
Vyhon3	1	2.3372	7.8764	2.9763	10.0304	3.0	10.0	FCSSgr2
Vyhon3	1	2.3373	7.8632	2.9765	10.0136	3.0	10.0	FCSSgr2
Vyhon3	1	2.3374	7.9716	2.9766	10.1516	3.0	10.0	FCSSgr2
Vyhon3	1	2.3393	7.7517	2.9790	9.8716	3.0	10.0	FCSSgr2
Vyhon3	1	2.3396	7.8276	2.9795	9.9682	3.0	10.0	FCSSgr2
Vyhon3	1	2.3397	7.8787	2.9796	10.0333	3.0	10.0	FCSSgr2

Vyhon3	1	2.3402	7.7208	2.9801	9.8322	3.0	10.0	FCSSgr2
Vyhon3	1	2.3408	7.7622	2.9809	9.8849	3.0	10.0	FCSSgr2
Vyhon3	1	2.3416	7.8325	2.9819	9.9745	3.0	10.0	FCSSgr2
Vyhon3	1	2.3428	7.7809	2.9835	9.9087	3.0	10.0	FCSSgr2
Vyhon3	1	2.3436	7.8423	2.9845	9.9869	3.0	10.0	FCSSgr2
Vyhon3	1	2.3439	7.7730	2.9849	9.8986	3.0	10.0	FCSSgr2
Vyhon3	1	2.3440	7.7365	2.9851	9.8523	3.0	10.0	FCSSgr2
Vyhon3	1	2.3450	7.7174	2.9863	9.8279	3.0	10.0	FCSSgr2
Vyhon3	1	2.3453	7.8589	2.9866	10.0080	3.0	10.0	FCSSgr2
Vyhon3	1	2.3482	7.8007	2.9904	9.9339	3.0	10.0	FCSSgr2
Vyhon3	1	2.3484	7.8410	2.9906	9.9853	3.0	10.0	FCSSgr2
Vyhon3	1	2.3496	7.7049	2.9921	9.8120	3.0	10.0	FCSSgr2
Vyhon3	1	2.3500	7.7681	2.9927	9.8925	3.0	10.0	FCSSgr2
Vyhon3	1	2.3513	7.8194	2.9943	9.9578	3.0	10.0	FCSSgr2
Vyhon3	1	2.3515	7.8178	2.9945	9.9558	3.0	10.0	FCSSgr2
Vyhon3	1	2.3517	7.7921	2.9949	9.9230	3.0	10.0	FCSSgr2
Vyhon3	1	2.3540	7.8519	2.9978	9.9992	3.0	10.0	FCSSgr2
Vyhon3	1	2.3544	7.8361	2.9983	9.9790	3.0	10.0	FCSSgr2
Vyhon3	1	2.3549	7.9053	2.9989	10.0672	3.0	10.0	FCSSgr2
Vyhon3	1	2.3556	7.8407	2.9998	9.9849	3.0	10.0	FCSSgr2
Vyhon3	1	2.3590	7.8588	3.0041	10.0079	3.0	10.0	FCSSgr2
Vyhon3	1	2.3629	7.7215	3.0091	9.8331	3.0	10.0	FCSSgr2
Vyhon3	1	2.3645	7.8651	3.0112	10.0160	3.0	10.0	FCSSgr2
Vyhon3	1	2.3665	7.7515	3.0136	9.8713	3.0	10.0	FCSSgr2
Vyhon3	1	2.3691	7.9188	3.0170	10.0843	3.0	10.0	FCSSgr2
Vyhon3	1	2.3713	7.9058	3.0198	10.0677	3.0	10.0	FCSSgr2
Vyhon3	1	2.3825	7.9632	3.0341	10.1409	3.0	10.0	FCSSgr2
Vyhon3	1	2.3932	7.9565	3.0477	10.1323	3.0	10.0	FCSSgr2
Vyhon3	1	2.3940	7.9332	3.0487	10.1026	3.0	10.0	FCSSgr2
Vyhon3	1	2.4116	7.9167	3.0711	10.0816	3.0	10.0	FCSSgr2
Vyhon3	1	2.4213	7.9358	3.0834	10.1060	3.0	10.0	FCSSgr2
Vyhon3	1	2.5832	8.0037	3.2897	10.1925	3.5	10.0	FCSSgr2
Vyhon3	1	3.9827	7.9717	5.0719	10.1517	5.0	10.0	FCSSgr2

Vyhon3	1	2.3798	8.0748	3.0306	10.2830	3.0	10.5	FCSSgr2
Vyhon3	1	2.4043	8.0681	3.0618	10.2744	3.0	10.5	FCSSgr2
Vyhon3	1	2.4304	8.2100	3.0950	10.4552	3.0	10.5	FCSSgr2
Vyhon3	1	2.4801	8.2868	3.1583	10.5530	3.0	10.5	FCSSgr2
Vyhon3	1	2.5657	9.3469	3.2674	11.9030	3.5	12.0	FCSSgr2
Vyhon3	1	2.4261	10.2389	3.0895	13.0390	3.0	13.0	FCSSgr2
Vyhon3	1	2.3321	11.4596	2.9699	14.5934	3.0	14.5	FCSSgr2
Vyhon3	1	2.3485	not recorded	2.9908	NA	3.0	NA	FCSSgr2
Vyhon4	1	2.1983	5.7672	2.8452	7.4642	3.0	7.5	FCSSgr2
Vyhon4	1	2.2766	5.8972	2.9465	7.6324	3.0	7.5	FCSSgr2
Vyhon4	1	2.2830	5.9140	2.9547	7.6542	3.0	7.5	FCSSgr2
Vyhon4	1	2.2898	5.8831	2.9636	7.6142	3.0	7.5	FCSSgr2
Vyhon4	1	2.3069	5.9623	2.9857	7.7167	3.0	7.5	FCSSgr2
Vyhon4	1	2.3455	5.8877	3.0357	7.6201	3.0	7.5	FCSSgr2
Vyhon4	1	3.6523	5.9576	4.7270	7.7106	4.5	7.5	FCSSgr2
Vyhon4	1	2.3114	6.0435	2.9915	7.8218	3.0	8.0	FCSSgr2
Vyhon4	1	2.3704	6.1275	3.0679	7.9305	3.0	8.0	FCSSgr2
Vyhon4	1	2.3897	6.2672	3.0928	8.1114	3.0	8.0	FCSSgr2
Vyhon4	1	2.4413	6.0919	3.1596	7.8844	3.0	8.0	FCSSgr2
Vyhon4	1	2.2853	6.7181	2.9577	8.6949	3.0	8.5	FCSSgr2
Vyhon4	1	2.3829	6.5221	3.0841	8.4412	3.0	8.5	FCSSgr2
Vyhon4	1	2.4021	6.4503	3.1089	8.3484	3.0	8.5	FCSSgr2
Vyhon4	1	2.2431	6.7866	2.9032	8.7835	3.0	9.0	FCSSgr2
Vyhon4	1	2.2616	7.1365	2.9270	9.2365	3.0	9.0	FCSSgr2
Vyhon4	1	2.2642	7.1272	2.9304	9.2244	3.0	9.0	FCSSgr2
Vyhon4	1	2.2658	7.1222	2.9325	9.2179	3.0	9.0	FCSSgr2
Vyhon4	1	2.2766	7.0149	2.9465	9.0790	3.0	9.0	FCSSgr2
Vyhon4	1	2.2812	6.9550	2.9525	9.0015	3.0	9.0	FCSSgr2
Vyhon4	1	2.2906	6.9828	2.9646	9.0376	3.0	9.0	FCSSgr2
Vyhon4	1	2.2964	6.9990	2.9721	9.0585	3.0	9.0	FCSSgr2
Vyhon4	1	2.3060	7.0378	2.9846	9.1086	3.0	9.0	FCSSgr2
Vyhon4	1	2.3096	7.0927	2.9892	9.1797	3.0	9.0	FCSSgr2
Vyhon4	1	2.3196	7.1183	3.0021	9.2129	3.0	9.0	FCSSgr2

Vyhon4	1	2.3211	7.0588	3.0041	9.1358	3.0	9.0	FCSSgr2
Vyhon4	1	2.3350	7.0661	3.0220	9.1453	3.0	9.0	FCSSgr2
Vyhon4	1	2.3449	6.8054	3.0349	8.8079	3.0	9.0	FCSSgr2
Vyhon4	1	2.3521	6.9611	3.0442	9.0095	3.0	9.0	FCSSgr2
Vyhon4	1	2.2627	7.4374	2.9285	9.6258	3.0	9.5	FCSSgr2
Vyhon4	1	2.2660	7.3029	2.9328	9.4518	3.0	9.5	FCSSgr2
Vyhon4	1	2.2747	7.3647	2.9441	9.5318	3.0	9.5	FCSSgr2
Vyhon4	1	2.2752	7.1987	2.9447	9.3169	3.0	9.5	FCSSgr2
Vyhon4	1	2.2781	7.2348	2.9484	9.3637	3.0	9.5	FCSSgr2
Vyhon4	1	2.2811	7.3035	2.9523	9.4525	3.0	9.5	FCSSgr2
Vyhon4	1	2.2878	7.4326	2.9610	9.6197	3.0	9.5	FCSSgr2
Vyhon4	1	2.2892	7.4190	2.9628	9.6020	3.0	9.5	FCSSgr2
Vyhon4	1	2.2900	7.4298	2.9638	9.6161	3.0	9.5	FCSSgr2
Vyhon4	1	2.2945	7.1989	2.9697	9.3172	3.0	9.5	FCSSgr2
Vyhon4	1	2.2947	7.4238	2.9700	9.6082	3.0	9.5	FCSSgr2
Vyhon4	1	2.2960	7.3719	2.9716	9.5411	3.0	9.5	FCSSgr2
Vyhon4	1	2.3030	7.3526	2.9806	9.5161	3.0	9.5	FCSSgr2
Vyhon4	1	2.3045	7.2014	2.9826	9.3204	3.0	9.5	FCSSgr2
Vyhon4	1	2.3094	7.3266	2.9889	9.4824	3.0	9.5	FCSSgr2
Vyhon4	1	2.3096	7.4898	2.9892	9.6937	3.0	9.5	FCSSgr2
Vyhon4	1	2.3105	7.5063	2.9904	9.7151	3.0	9.5	FCSSgr2
Vyhon4	1	2.3117	7.2376	2.9919	9.3673	3.0	9.5	FCSSgr2
Vyhon4	1	2.3121	7.2625	2.9925	9.3994	3.0	9.5	FCSSgr2
Vyhon4	1	2.3161	7.1521	2.9976	9.2566	3.0	9.5	FCSSgr2
Vyhon4	1	2.3188	7.3609	3.0011	9.5269	3.0	9.5	FCSSgr2
Vyhon4	1	2.3248	7.1622	3.0088	9.2697	3.0	9.5	FCSSgr2
Vyhon4	1	2.3353	7.4766	3.0225	9.6766	3.0	9.5	FCSSgr2
Vyhon4	1	2.3431	7.1733	3.0326	9.2840	3.0	9.5	FCSSgr2
Vyhon4	1	2.3451	7.2065	3.0351	9.3270	3.0	9.5	FCSSgr2
Vyhon4	1	2.3469	7.5246	3.0375	9.7387	3.0	9.5	FCSSgr2
Vyhon4	1	2.3472	7.3622	3.0379	9.5285	3.0	9.5	FCSSgr2
Vyhon4	1	2.3558	7.2692	3.0489	9.4081	3.0	9.5	FCSSgr2
Vyhon4	1	2.3651	7.3667	3.0610	9.5344	3.0	9.5	FCSSgr2

Vyhon4	1	2.3736	7.3580	3.0720	9.5231	3.0	9.5	FCSSgr2
Vyhon4	1	2.3757	7.3528	3.0747	9.5164	3.0	9.5	FCSSgr2
Vyhon4	1	2.4121	7.4086	3.1219	9.5885	3.0	9.5	FCSSgr2
Vyhon4	1	2.4260	7.4483	3.1398	9.6399	3.0	9.5	FCSSgr2
Vyhon4	1	2.3078	7.6254	2.9868	9.8691	3.0	10.0	FCSSgr2
Vyhon4	1	2.3595	7.6825	3.0537	9.9431	3.0	10.0	FCSSgr2
Vyhon4	1	2.3864	7.6017	3.0886	9.8385	3.0	10.0	FCSSgr2
Vyhon4	1	2.2849	9.1295	2.9572	11.8159	3.0	12.0	FCSSgr2
Vyhon4	1	2.3582	9.5399	3.0520	12.3470	3.0	12.5	FCSSgr2
Vyhon4	1	2.2434	not recorded	2.9035	NA	3.0	NA	FCSSgr2
Vyhon4	1	2.2997	not recorded	2.9764	NA	3.0	NA	FCSSgr2
Vyhon4	1	2.3050	not recorded	2.9832	NA	3.0	NA	FCSSgr2
Vyhon4	1	2.3115	not recorded	2.9917	NA	3.0	NA	FCSSgr2
Vyhon4	1	2.3191	not recorded	3.0015	NA	3.0	NA	FCSSgr2
Vyhon4	1	4.4674	not recorded	5.7819	NA	6.0	NA	FCSSgr2
Vyhon8	1	2.3384	5.5123	3.0074	7.0895	3.0	7.0	FCSSgr2
Vyhon8	1	2.2899	5.8578	2.9451	7.5339	3.0	7.5	FCSSgr2
Vyhon8	1	2.3004	5.8476	2.9586	7.5208	3.0	7.5	FCSSgr2
Vyhon8	1	2.3031	5.8622	2.9621	7.5396	3.0	7.5	FCSSgr2
Vyhon8	1	2.3035	5.9015	2.9625	7.5902	3.0	7.5	FCSSgr2
Vyhon8	1	2.3060	5.9831	2.9659	7.6950	3.0	7.5	FCSSgr2
Vyhon8	1	2.3102	5.8526	2.9712	7.5272	3.0	7.5	FCSSgr2
Vyhon8	1	2.3172	5.9927	2.9803	7.7074	3.0	7.5	FCSSgr2
Vyhon8	1	2.3301	5.6843	2.9968	7.3108	3.0	7.5	FCSSgr2
Vyhon8	1	2.3508	5.7883	3.0235	7.4445	3.0	7.5	FCSSgr2
Vyhon8	1	3.6766	5.9027	4.7286	7.5916	4.5	7.5	FCSSgr2
Vyhon8	1	2.3362	6.0921	3.0046	7.8352	3.0	8.0	FCSSgr2
Vyhon8	1	2.3377	6.3616	3.0066	8.1819	3.0	8.0	FCSSgr2
Vyhon8	1	2.3207	6.7010	2.9847	8.6184	3.0	8.5	FCSSgr2
Vyhon8	1	2.2317	6.9957	2.8703	8.9974	3.0	9.0	FCSSgr2
Vyhon8	1	2.2869	7.0080	2.9413	9.0133	3.0	9.0	FCSSgr2
Vyhon8	1	2.2877	7.1141	2.9423	9.1496	3.0	9.0	FCSSgr2
Vyhon8	1	2.2892	6.9119	2.9441	8.8896	3.0	9.0	FCSSgr2

Vyhon8	1	2.2914	7.0061	2.9471	9.0108	3.0	9.0	FCSSgr2
Vyhon8	1	2.2917	6.8345	2.9475	8.7900	3.0	9.0	FCSSgr2
Vyhon8	1	2.3008	7.1480	2.9591	9.1932	3.0	9.0	FCSSgr2
Vyhon8	1	2.3041	7.0688	2.9633	9.0914	3.0	9.0	FCSSgr2
Vyhon8	1	2.3057	7.1323	2.9654	9.1731	3.0	9.0	FCSSgr2
Vyhon8	1	2.3069	6.9477	2.9670	8.9357	3.0	9.0	FCSSgr2
Vyhon8	1	2.3111	7.0013	2.9724	9.0046	3.0	9.0	FCSSgr2
Vyhon8	1	2.3113	6.9967	2.9726	8.9987	3.0	9.0	FCSSgr2
Vyhon8	1	2.3145	7.1901	2.9768	9.2474	3.0	9.0	FCSSgr2
Vyhon8	1	2.3194	7.1181	2.9831	9.1548	3.0	9.0	FCSSgr2
Vyhon8	1	2.3202	7.1221	2.9840	9.1599	3.0	9.0	FCSSgr2
Vyhon8	1	2.3253	7.0309	2.9906	9.0426	3.0	9.0	FCSSgr2
Vyhon8	1	2.3277	6.8705	2.9937	8.8364	3.0	9.0	FCSSgr2
Vyhon8	1	2.3355	7.0281	3.0038	9.0391	3.0	9.0	FCSSgr2
Vyhon8	1	2.3362	6.8798	3.0047	8.8483	3.0	9.0	FCSSgr2
Vyhon8	1	2.3483	7.1187	3.0203	9.1556	3.0	9.0	FCSSgr2
Vyhon8	1	2.3500	7.0058	3.0224	9.0104	3.0	9.0	FCSSgr2
Vyhon8	1	2.3562	6.9550	3.0303	8.9451	3.0	9.0	FCSSgr2
Vyhon8	1	2.4414	7.0568	3.1399	9.0759	3.0	9.0	FCSSgr2
Vyhon8	1	2.4609	7.1293	3.1651	9.1692	3.0	9.0	FCSSgr2
Vyhon8	1	2.2762	7.2694	2.9275	9.3494	3.0	9.5	FCSSgr2
Vyhon8	1	2.2847	7.2217	2.9384	9.2880	3.0	9.5	FCSSgr2
Vyhon8	1	2.2893	7.2086	2.9444	9.2712	3.0	9.5	FCSSgr2
Vyhon8	1	2.2974	7.2708	2.9548	9.3512	3.0	9.5	FCSSgr2
Vyhon8	1	2.3081	7.2934	2.9685	9.3802	3.0	9.5	FCSSgr2
Vyhon8	1	2.3149	7.3157	2.9773	9.4089	3.0	9.5	FCSSgr2
Vyhon8	1	2.3177	7.5730	2.9808	9.7399	3.0	9.5	FCSSgr2
Vyhon8	1	2.3194	7.2413	2.9831	9.3133	3.0	9.5	FCSSgr2
Vyhon8	1	2.3220	7.4482	2.9864	9.5793	3.0	9.5	FCSSgr2
Vyhon8	1	2.3268	7.4793	2.9925	9.6194	3.0	9.5	FCSSgr2
Vyhon8	1	2.3315	7.3553	2.9986	9.4598	3.0	9.5	FCSSgr2
Vyhon8	1	2.3326	7.5234	3.0001	9.6760	3.0	9.5	FCSSgr2
Vyhon8	1	2.3370	7.4735	3.0056	9.6118	3.0	9.5	FCSSgr2

Vyhon8	1	2.3403	7.2371	3.0099	9.3079	3.0	9.5	FCSSgr2
Vyhon8	1	2.3430	7.2747	3.0134	9.3562	3.0	9.5	FCSSgr2
Vyhon8	1	2.3449	7.4166	3.0158	9.5388	3.0	9.5	FCSSgr2
Vyhon8	1	2.3475	7.3140	3.0192	9.4068	3.0	9.5	FCSSgr2
Vyhon8	1	2.3478	7.2216	3.0196	9.2879	3.0	9.5	FCSSgr2
Vyhon8	1	2.3496	7.3792	3.0219	9.4906	3.0	9.5	FCSSgr2
Vyhon8	1	2.3564	7.3380	3.0306	9.4377	3.0	9.5	FCSSgr2
Vyhon8	1	2.3577	7.5285	3.0323	9.6826	3.0	9.5	FCSSgr2
Vyhon8	1	2.3593	7.2340	3.0343	9.3039	3.0	9.5	FCSSgr2
Vyhon8	1	2.3598	7.5169	3.0350	9.6677	3.0	9.5	FCSSgr2
Vyhon8	1	2.3681	7.4963	3.0456	9.6412	3.0	9.5	FCSSgr2
Vyhon8	1	2.3766	7.4289	3.0567	9.5546	3.0	9.5	FCSSgr2
Vyhon8	1	2.3771	7.2620	3.0573	9.3399	3.0	9.5	FCSSgr2
Vyhon8	1	2.3796	7.5526	3.0604	9.7137	3.0	9.5	FCSSgr2
Vyhon8	1	2.3838	7.4116	3.0659	9.5323	3.0	9.5	FCSSgr2
Vyhon8	1	2.3875	7.3113	3.0707	9.4033	3.0	9.5	FCSSgr2
Vyhon8	1	2.4129	7.3998	3.1033	9.5171	3.0	9.5	FCSSgr2
Vyhon8	1	2.3526	7.6625	3.0257	9.8550	3.0	10.0	FCSSgr2
Vyhon8	1	2.3666	7.5925	3.0438	9.7650	3.0	10.0	FCSSgr2
Vyhon8	1	2.4215	7.7402	3.1143	9.9549	3.0	10.0	FCSSgr2
Vyhon8	1	2.2995	9.3038	2.9575	11.9659	3.0	12.0	FCSSgr2
Vyhon8	1	2.3575	9.5113	3.0320	12.2327	3.0	12.0	FCSSgr2
Vyhon8	1	2.2973	not recorded	2.9546	NA	3.0	NA	FCSSgr2
Vyhon8	1	2.3017	not recorded	2.9602	NA	3.0	NA	FCSSgr2
Vyhon8	1	2.3131	not recorded	2.9749	NA	3.0	NA	FCSSgr2
Vyhon8	1	2.3769	not recorded	3.0570	NA	3.0	NA	FCSSgr2

4x plants

plant	seeds	embryo	endosperm	ploidy_emb_ca	ploidy_end_cal	emb_level_est	end_level_est	group
MAJ16-1	1	2.9763	9.0488	3.9525	12.0166	4.0	12.0	x
MAJ16-1	1	2.9763	9.0284	3.9525	11.9895	4.0	12.0	x
MAJ16-1	1	2.9763	9.1098	3.9525	12.0977	4.0	12.0	x

MAJ16-1	1	3.0388	9.1783	4.0355	12.1886	4.0	12.0	x
MAJ16-1	1	3.0388	4.5945	4.0355	6.1013	4.0	6.0	x
MAJ16-1	1	3.0388	9.0841	4.0355	12.0635	4.0	12.0	x
MAJ16-1	1	3.0048	9.1211	3.9903	12.1127	4.0	12.0	x
MAJ16-1	1	3.0048	9.1209	3.9903	12.1124	4.0	12.0	x
MAJ16-1	1	3.0048	9.1716	3.9903	12.1797	4.0	12.0	x
MAJ16-1	1	3.0157	9.1479	4.0047	12.1482	4.0	12.0	x
MAJ16-1	1	3.0157	9.1208	4.0047	12.1122	4.0	12.0	x
MAJ16-1	1	3.0157	9.1576	4.0047	12.1611	4.0	12.0	x
MAJ16-1	1	3.1068	9.4367	4.1257	12.5317	4.0	12.5	x
MAJ16-1	1	1.4950	6.2486	1.9853	8.2980	2.0	8.0	x
MAJ16-1	1	3.1068	9.3453	4.1257	12.4103	4.0	12.5	x
MAJ16-1	1	3.0069	9.0681	3.9931	12.0423	4.0	12.0	x
MAJ16-1	1	3.0069	9.2338	3.9931	12.2622	4.0	12.5	x
MAJ16-1	1	3.0069	9.3215	3.9931	12.3788	4.0	12.5	x
MAJ16-1	1	3.0174	9.1519	4.0070	12.1536	4.0	12.0	x
MAJ16-1	1	3.0174	9.0451	4.0070	12.0117	4.0	12.0	x
MAJ16-1	1	3.0174	8.0245	4.0070	10.6564	4.0	10.5	x
MAJ16-1	1	2.9697	10.3514	3.9437	13.7465	4.0	13.5	x
MAJ16-1	1	2.9697	8.9513	3.9437	11.8871	4.0	12.0	x
MAJ16-1	1	2.9697	9.0306	3.9437	11.9925	4.0	12.0	x
MAJ16-1	1	2.9725	7.4960	3.9475	9.9545	4.0	10.0	x
MAJ16-1	1	2.9725	9.0353	3.9475	11.9987	4.0	12.0	x
MAJ16-1	1	2.9725	9.1056	3.9475	12.0920	4.0	12.0	x
MAJ16-2	1	2.9653	10.7410	3.9530	14.3186	4.0	14.5	x
MAJ16-2	1	2.9653	10.3249	3.9530	13.7639	4.0	14.0	x
MAJ16-2	1	2.9653	8.8638	3.9530	11.8162	4.0	12.0	x
MAJ16-2	1	3.0941	9.2507	4.1246	12.3319	4.0	12.5	x
MAJ16-2	1	3.0941	11.0688	4.1246	14.7556	4.0	15.0	x
MAJ16-2	1	3.0941	9.0858	4.1246	12.1121	4.0	12.0	x
MAJ16-2	1	2.9619	8.9050	3.9485	11.8710	4.0	12.0	x
MAJ16-2	1	2.9619	8.7882	3.9485	11.7153	4.0	11.5	x
MAJ16-2	1	2.9619	7.4563	3.9485	9.9398	4.0	10.0	x

MAJ16-2	1	2.9911	12.0203	3.9874	16.0241	4.0	16.0	x
MAJ16-2	1	2.9911	9.0508	3.9874	12.0655	4.0	12.0	x
MAJ16-2	1	2.9911	8.9598	3.9874	11.9441	4.0	12.0	x
MAJ16-2	1	3.0598	not recorded	4.0789	NA	4.0	NA	x
MAJ16-2	1	3.0598	9.2176	4.0789	12.2878	4.0	12.5	x
MAJ16-2	1	3.0598	9.1050	4.0789	12.1377	4.0	12.0	x
MAJ16-2	1	3.0036	8.8930	4.0040	11.8550	4.0	12.0	x
MAJ16-2	1	3.0036	10.3308	4.0040	13.7718	4.0	14.0	x
MAJ16-2	1	3.0036	7.4860	4.0040	9.9794	4.0	10.0	x
MAJ16-2	1	2.9252	8.7915	3.8996	11.7197	4.0	11.5	x
MAJ16-2	1	2.9252	8.7727	3.8996	11.6947	4.0	11.5	x
MAJ16-2	1	2.9252	8.9051	3.8996	11.8712	4.0	12.0	x
MAJ16-2	1	3.0036	9.0164	4.0040	12.0195	4.0	12.0	x
MAJ16-2	1	3.0036	not recorded	4.0040	NA	4.0	NA	x
MAJ16-2	1	3.0036	9.0600	4.0040	12.0776	4.0	12.0	x
RaznH16-3	1	2.9557	9.0425	3.9687	12.1414	4.0	12.0	x
RaznH16-3	1	2.9557	14.5127	3.9687	19.4865	4.0	19.5	x
RaznH16-3	1	2.9557	8.8184	3.9687	11.8406	4.0	12.0	x
RaznH16-3	1	2.9934	12.1683	4.0193	16.3386	4.0	16.5	x
RaznH16-3	1	2.9934	8.9447	4.0193	12.0103	4.0	12.0	x
RaznH16-3	1	2.9934	4.5196	4.0193	6.0686	4.0	6.0	x
RaznH16-3	1	2.9806	8.9642	4.0021	12.0364	4.0	12.0	x
RaznH16-3	1	2.9806	9.0107	4.0021	12.0989	4.0	12.0	x
RaznH16-3	1	2.9806	8.9541	4.0021	12.0228	4.0	12.0	x
RaznH16-3	1	2.9835	9.1604	4.0059	12.2998	4.0	12.5	x
RaznH16-3	1	2.9835	9.0314	4.0059	12.1266	4.0	12.0	x
RaznH16-3	1	2.9835	10.5216	4.0059	14.1275	4.0	14.0	x
RaznH16-3	1	2.9821	9.0457	4.0041	12.1457	4.0	12.0	x
RaznH16-3	1	2.9821	8.9199	4.0041	11.9768	4.0	12.0	x
RaznH16-3	1	2.9821	11.9416	4.0041	16.0342	4.0	16.0	x
PodSosnom16-	1	2.9884	9.0714	4.0050	12.1575	4.0	12.0	x
PodSosnom16-	1	2.9884	8.3072	4.0050	11.1333	4.0	11.0	x
PodSosnom16-	1	1.4885	6.0510	1.9949	8.1095	2.0	8.0	x

PodSosnom16-	1	2.9809	9.0555	3.9950	12.1362	4.0	12.0	x
PodSosnom16-	1	2.9809	8.8944	3.9950	11.9202	4.0	12.0	x
PodSosnom16-	1	2.9811	10.3340	4.0248	13.9524	4.0	14.0	x
PodSosnom16-	1	2.9811	10.5326	4.0248	14.2205	4.0	14.0	x
PodSosnom16-	1	2.9811	9.0652	4.0248	12.2392	4.0	12.0	x
PodSosnom16-	1	2.9255	8.9580	3.9499	12.0945	4.0	12.0	x
PodSosnom16-	1	2.9255	10.6223	3.9499	14.3416	4.0	14.5	x
PodSosnom16-	1	2.9255	7.4706	3.9499	10.0863	4.0	10.0	x
PodSosnom16-	1	2.9814	7.5351	4.0253	10.1735	4.0	10.0	x
PodSosnom16-	1	2.9814	not recorded	4.0253	NA	4.0	NA	x
VYHON16-6POI	1	3.0064	8.9668	4.0383	12.0446	4.0	12.0	x
VYHON16-6POI	1	3.0064	9.0846	4.0383	12.2028	4.0	12.0	x
VYHON16-6POI	1	3.0064	9.0583	4.0383	12.1675	4.0	12.0	x
VYHON16-6POI	1	4.4635	7.4439	5.9956	9.9989	6.0	10.0	x
VYHON16-6POI	1	2.9765	9.0103	3.9982	12.1030	4.0	12.0	x
VYHON16-6POI	1	2.9765	8.9024	3.9982	11.9581	4.0	12.0	x
VYHON16-6POI	1	2.9542	9.1132	3.9681	12.2412	4.0	12.0	x
VYHON16-6POI	1	2.9542	7.5860	3.9681	10.1898	4.0	10.0	x
VYHON16-6POI	1	2.9542	12.1312	3.9681	16.2951	4.0	16.5	x
VYHON16-6POI	1	2.9745	9.1475	3.9954	12.2873	4.0	12.5	x
VYHON2	1	3.0287	9.2156	4.0670	12.3749	4.0	12.5	x
VYHON2	1	3.0287	9.1321	4.0670	12.2628	4.0	12.5	x
VYHON2	1	3.0287	7.6741	4.0670	10.3050	4.0	10.5	x
VYHON2	1	2.9506	4.3999	3.9621	5.9083	4.0	6.0	x
VYHON2	1	2.9506	8.9279	3.9621	11.9887	4.0	12.0	x
VYHON2	1	2.9506	8.9494	3.9621	12.0174	4.0	12.0	x
VYHON2	1	2.9571	8.9589	3.9709	12.0303	4.0	12.0	x
VYHON2	1	2.9571	9.1775	3.9709	12.3238	4.0	12.5	x
VYHON16-15	1	3.0154	9.1248	4.0609	12.2886	4.0	12.5	x
VYHON16-15	1	3.0154	9.1885	4.0609	12.3745	4.0	12.5	x
VYHON16-15	1	3.0154	9.2879	4.0609	12.5083	4.0	12.5	x
VYHON16-15	1	2.9612	9.0530	3.9879	12.1920	4.0	12.0	x
VYHON16-15	1	4.5415	7.6081	6.1162	10.2461	6.0	10.0	x

VYHON16-15	1	2.9612	9.0782	3.9879	12.2260	4.0	12.0	x
VYHON16-15	1	1.4598	6.1141	1.9660	8.2341	2.0	8.0	x
VYHON16-15	1	2.9584	4.5393	3.9842	6.1132	4.0	6.0	x
VYHON16-15	1	2.9584	9.0022	3.9842	12.1235	4.0	12.0	x
VYHON16-15	1	2.9944	8.8079	4.0327	11.8619	4.0	12.0	x
VYHON16-15	1	2.9944	7.5710	4.0327	10.1961	4.0	10.0	x
VYHON16-15	1	2.9944	9.0666	4.0327	12.2103	4.0	12.0	x
VYHON16-15	1	1.4262	6.2762	1.9207	8.4524	2.0	8.5	x
VYHON16-15	1	2.9213	7.4002	3.9343	9.9661	4.0	10.0	x
VYHON16-15	1	1.4262	7.2532	1.9207	9.7681	2.0	9.5	x
VYHON16-15	1	1.3796	6.0051	1.8579	8.0873	2.0	8.0	x
VYHON16-15	1	6.0247	not recorded	8.1137	NA	8.0	NA	x
